# Linking life history to landscape for threatened species conservation in a multiuse region

**DOI:** 10.1111/cobi.13989

**Published:** 2022-11-16

**Authors:** Robyn E. Shaw, Peter B. Spencer, Lesley A. Gibson, Judy A. Dunlop, Janine E. Kinloch, Karel Mokany, Margaret Byrne, Craig Moritz, Harriet Davie, Kenny J. Travouillon, Kym M. Ottewell

**Affiliations:** ^1^ Environmental & Conservation Sciences Murdoch University Perth Western Australia Australia; ^2^ Biodiversity and Conservation Science Department of Biodiversity, Conservation and Attractions Perth Western Australia Australia; ^3^ Division of Ecology and Evolution, Research School of Biology The Australian National University, Australian Capital Territory Canberra Australia; ^4^ WA Feral Cat Working Group Perth Western Australia Australia; ^5^ CSIRO Canberra Australian Capital Territory Australia; ^6^ Roy Hill Iron Ore Pty Ltd Perth Western Australia Australia; ^7^ Collection and Research Western Australian Museum Perth Western Australia Australia

**Keywords:** arid, connectivity, conservation planning, landscape ecology, landscape genetics, mammals, northern quoll, species distribution modeling, árido, conectividad, cuol del norte, ecología del paisaje, genética del paisaje, mamíferos, modelado de distribución de especies, planeación de la conservación

## Abstract

Landscape‐scale conservation that considers metapopulation dynamics will be essential for preventing declines of species facing multiple threats to their survival. Toward this end, we developed a novel approach that combines occurrence records, spatial–environmental data, and genetic information to model habitat, connectivity, and patterns of genetic structure and link spatial attributes to underlying ecological mechanisms. Using the threatened northern quoll (*Dasyurus hallucatus*) as a case study, we applied this approach to address the need for conservation decision‐making tools that promote resilient metapopulations of this threatened species in the Pilbara, Western Australia, a multiuse landscape that is a hotspot for biodiversity and mining. Habitat and connectivity were predicted by different landscape characteristics. Whereas habitat suitability was overwhelmingly driven by terrain ruggedness, dispersal was facilitated by proximity to watercourses. Although there is limited evidence for major physical barriers in the Pilbara, areas with high silt and clay content (i.e., alluvial and hardpan plains) showed high resistance to dispersal. Climate subtlety shaped distributions and patterns of genetic turnover, suggesting the potential for local adaptation. By understanding these spatial–environmental associations and linking them to life‐history and metapopulation dynamics, we highlight opportunities to provide targeted species management. To support this, we have created habitat, connectivity, and genetic uniqueness maps for conservation decision‐making in the region. These tools have the potential to provide a more holistic approach to conservation in multiuse landscapes globally.

## Resumen

La conservación a nivel del paisaje que incluye las dinámicas metapoblacionales será esencial para prevenir la declinación de especies con múltiples amenazas a su supervivencia. Enfocados en este fin, desarrollamos una estrategia novedosa que combina los registros presenciales, datos espacio‐ambientales e información genética para modelar la conectividad de hábitat y los patrones de estructura genética y conectar los atributos espaciales con los mecanismos ecológicos subyacentes. Usamos al cuol del norte (*Dasyurus hallucatus*) como estudio de caso para aplicar esta estrategia y abordar la necesidad de herramientas de decisión en la conservación que promuevan metapoblaciones resilientes de esta especie en la Pilbara de Australia Occidental, un paisaje multiusos que es un punto caliente para la biodiversidad y la minería. Diferentes características del paisaje pronosticaron la conectividad y el hábitat. Mientras que la escabrosidad del terreno causó enormemente la idoneidad del hábitat, la dispersión estuvo propiciada por la proximidad a los cauces. Aunque hay evidencias limitadas de barreras físicas importantes en la Pilbara, las áreas con un contenido elevado de limo y arcilla (es decir, planicies aluviales y de alio) mostraron una gran resistencia a la dispersión. La matización climática determinó la distribución y los patrones del recambio genético, lo que sugiere un potencial para la adaptación local. Si entendemos estas asociaciones espacio‐ambientales y las conectamos con las dinámicas metapoblacionales y de historia de vida, podemos resaltar las oportunidades para proporcionar un manejo focalizado de la especie. Para respaldar esto hemos creado mapas de hábitat, conectividad y singularidad genética para las decisiones de conservación en la región. Estas herramientas tienen el potencial de proporcionar una estrategia más holística para la conservación en los paisajes multiusos de todo el mundo.

## INTRODUCTION

Incorporating complex genetic information into real‐world conservation planning represents a key challenge for global efforts, leading to growing concern that fundamental evolutionary concepts are not being adequately captured in management strategies (Cook & Sgrò, 2017). Although the importance of genetic diversity for species persistence is generally well understood, there is frequently a lack of guidance about how to apply this knowledge (Taft et al., 2020). Collaborative partnerships among researchers, conservation agencies, and practitioners can facilitate the use of genetics in policy and practice through the development of decision‐support tools (Rosauer et al., 2016; Taft et al., 2020).

Species distribution models (SDMs) are an example of how complex modeling has been incorporated into conservation through user‐friendly decision‐support tools (i.e., maps). These models have helped inform survey design (Aizpurua et al., 2015), identify habitat for reintroductions (Westwood et al., 2020), and make predictions about the impacts of climate change on vulnerable species (Razgour et al., 2018). Although they have improved knowledge of species’ habitat requirements, their use in conservation has sometimes been criticized (Addison et al., 2013; Morales et al., 2017). This is partly because SDMs are only as powerful as the biological knowledge, where broad‐scale environmental correlations can be linked to the underlying demography and life history (Merow et al., 2014). Process‐based approaches, such as mechanistic SDMs, are bridging this gap, but due to a lack of data, they remain out of reach for examination of most species (Evans et al., 2016). Combining SDMs with landscape connectivity modeling represents a pragmatic approach to disentangle the complex ways populations interact with their environment, providing a valuable and complementary source of information on species’ ecology.

The field of landscape genetics brings together landscape ecology, spatial statistics, and population genetics to quantify how genetic variation is distributed in space (Manel & Holderegger, 2013). Gene flow is used to describe whether an organism moved, survived, and reproduced, reflecting the functional connectivity of a landscape. Landscape genetic studies have revealed physical barriers to dispersal (isolation by barrier [IBB]) (Vignieri, 2005), how landscape attributes influence realized dispersal (isolation by resistance [IBR]) (McRae, 2006), and how local adaptation to the environment drives patterns of genetic turnover (isolation by environment [IBE]) (Fitzpatrick & Keller, 2015; Wang & Bradburd, 2014). By linking these analyses with measures of genetic diversity, it is possible to identify refugia, dispersal pathways, and areas with increased inbreeding and genetic drift due to isolation or fragmentation (Wang et al., 2017). Crucially, results can be viewed spatially because genetic data are analyzed with spatial–environmental variables, which increases the potential for uptake by managers. Such decision‐support tools are of particularly high importance in multiuse landscapes where spatial prioritization of actions is key.

The culturally significant Pilbara region of Western Australia is a biodiversity, mining, agricultural, and tourism hotspot (Government of Western Australia, 2017; McKenzie et al., 2009; Pepper et al., 2013). The region harbors many endemic and threatened species, including the northern quoll (*Dasyurus hallucatus*), a declining dasyurid marsupial listed as endangered by the IUCN, and Australian federal and state legislation. Northern quolls are vulnerable to land clearing, changed fire regimes, grazing, predation by feral cats (*Felis catus*), and poisoning by the invasive cane toad (*Rhinella marina*) (Moore, Dunlop, et al., 2021) (Appendix S1). The absence of cane toads and the Pilbara's high topographic complexity, which provides protection from predators, make it one of the last strongholds for the species (Moore et al., 2019). With competing priorities for land use in this region, understanding the relationship between landscape characteristics and ecological processes will be critical for supporting resilient populations. Given the wealth of research on northern quolls (Moore, Dunlop, et al., 2021) (Appendix S1), this species is an ideal candidate for developing landscape‐scale spatial‐genetic tools to guide conservation strategies.

**FIGURE 1:**
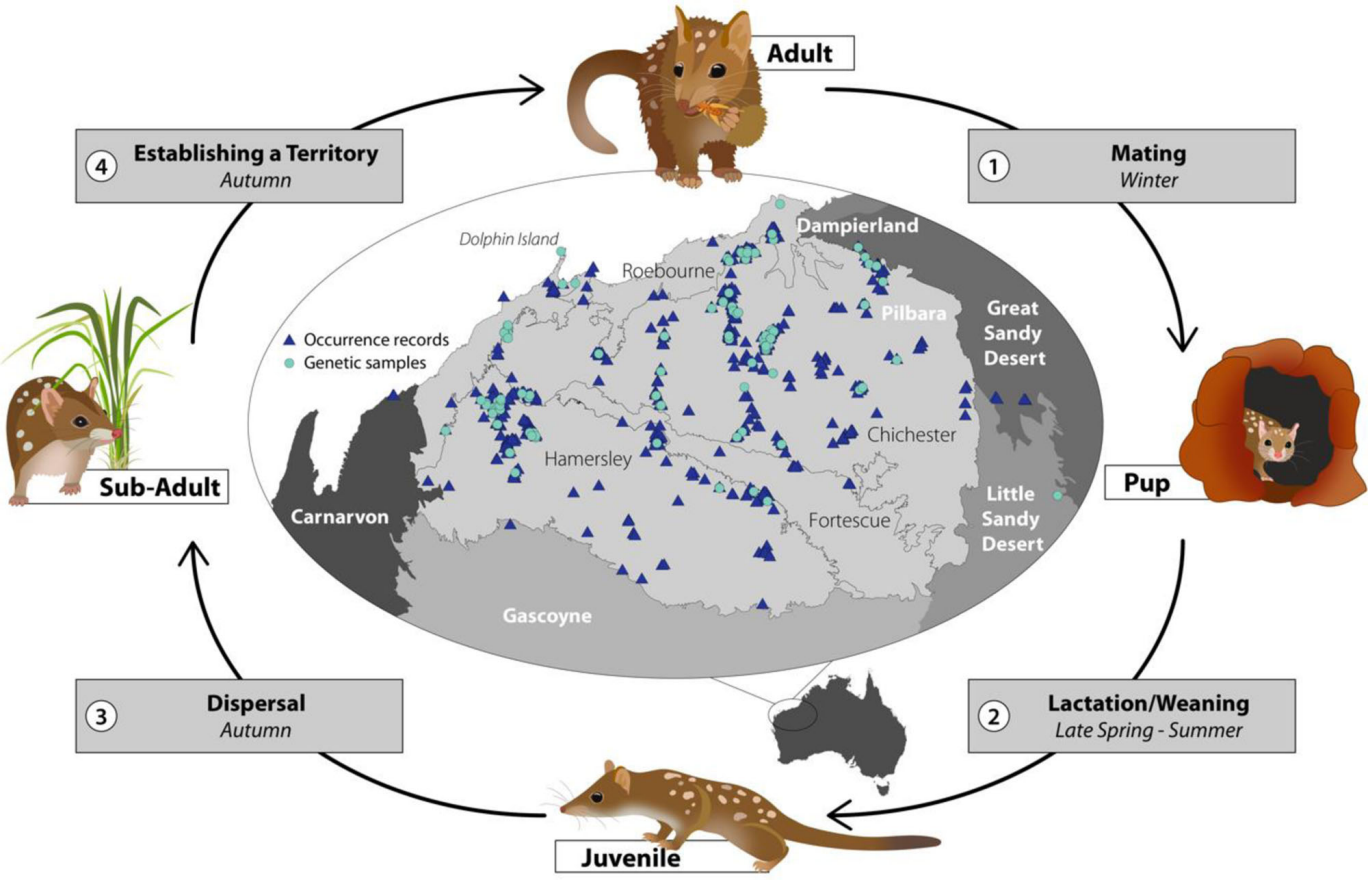
Pilbara region with northern quoll genetic sample and record locations and the species’ life cycle (white lettering, Interim Biogeographic Regionalisation for Australia [IBRA] regions; black lettering, IBRA subregions; numbers, referred to in Figure 2)

**FIGURE 2:**
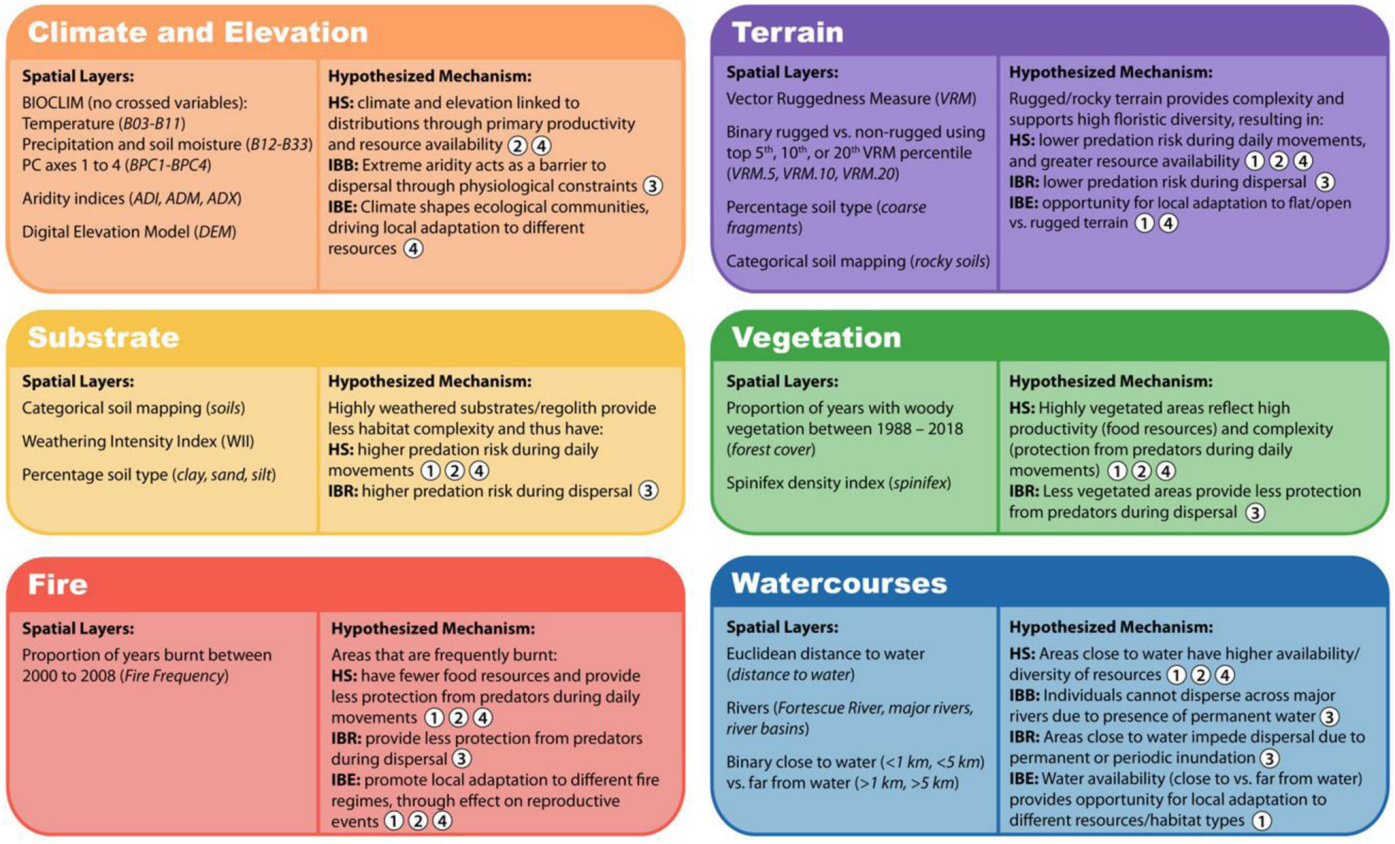
Spatial layers used in species distribution modeling and landscape genetic analyses, the hypothesized mechanism driving spatial‐environmental associations, and how these link to the life cycle and demography of the northern quoll (Figure 1) (hypotheses: HS, habitat suitability; IBB, isolation by barrier; IBR, isolation by resistance; IBE, isolation by environment; numbers, life cycle stages shown in Figure 1)

We used species distribution modeling and landscape genetics to identify spatial–environmental associations between northern quoll occurrence and patterns of genetic diversity and structure across the Pilbara. We built on previous SDMs (Molloy et al., 2017; Moore et al., 2019) by explicitly linking spatial hypotheses to life history (Figures 1 & 2). We combined these findings with landscape genetic analyses to identify critical habitat (i.e., areas critical to the survival of a listed threatened species under Australia's Environment Protection and Biodiversity Conservation Act) (Australian Government, 2000), including habitat used to meet essential life cycle requirements (e.g., foraging and breeding), habitat corridors between these sites, and habitat necessary for maintaining genetic diversity. We tested specific hypotheses (Figure 2) to address 4 key questions. Which environmental attributes (climate, elevation, substrate, terrain, fire, vegetation, or watercourses) describe critical habitat, and what are the ecological mechanisms driving these associations? Do physical barriers (IBB—watercourses or aridity), the quality of the landscape (IBR—substrate, terrain, fire, watercourses, or vegetation), or both restrict dispersal, or is movement limited only by dispersal capacity (isolation by distance [IBD])? Can areas with high or low genetic diversity be identified, and do these correspond to areas that are highly connected or isolated? Do environmental factors (IBE [climate, terrain, fire regime, or water availability]) drive genetic differences that could result in local adaptation?

We developed an analytical framework for incorporating ecological and evolutionary processes into conservation decision‐making that is relevant and scalable to other multiuse landscapes where anthropogenic disturbances are pervasive (e.g., peri‐urban interface [Fusco et al., 2021]). Given the complexity of meeting the land‐use needs of diverse stakeholders in these areas, a methodology that facilitates spatial prioritization of habitat critical for metapopulation persistence will help practitioners focus conservation attention on areas that build resilience of target species.

## METHODS

### Study location and species

The Pilbara biogeographic region in northwestern Australia covers ∼179,000 km^2^, encompassing the Chichester, Fortescue, Hamersley, and Roebourne Interim Biogeographic Regionalisation for Australia (IBRA) subregions (Figure 1). The Chichester is dominated by granite and basalt plains with Acacia shrub steppe, whereas there are alluvial plains in the Fortescue, sedimentary ranges with Mulga woodland and hummock grasses in the Hamersley, and coastal plains in Roebourne (McKenzie et al., 2009). The Hamersley Range and areas near the coast experience a hot semiarid tropical climate, and the rest of the region has a hot arid climate.

The Pilbara supports a genetically and geographically discrete population of northern quolls (Hohnen et al., 2016), known as dheebooga, thindooga, an'doola, wayinwarra, wiminji, and ngawungawu across language groups in the region (Abbott, 2013). Northern quolls are the largest marsupial predator in northern Australia, although their varied diet consists of plants, invertebrates, and small vertebrates (Dunlop et al., 2017).

### Environmental variables

We developed hypotheses regarding important environmental variables and the mechanisms that drive broad‐scale spatial associations over the life cycle of the northern quoll (Figures 1 & 2) through a literature review (Appendix S1). We obtained or derived spatial layers to represent key variables identified during this process (Figure 2; Appendices S2 & S3). These included BIOCLIM variables (and principal components, excluding composite variables combining rainfall and temperature), aridity indices, substrate, terrain ruggedness, elevation, watercourses, vegetation, and fire (acronyms for spatial layers are defined in Figure 2). All rasters were aggregated to a 1‐km^2^ resolution, except those used for testing IBR (described below).

### Species distribution modeling

Northern quoll occurrence records were downloaded on 6 September 2020 from the Atlas of Living Australia, with ALA4R (Newman et al., 2019) in R 3.6.3 (R Core Team, 2020), and from the Western Australian NatureMap database (DBCA, 2019) (Appendix S4). We filtered records from the Pilbara and surrounding areas and excluded pre‐2000 records to represent contemporary distribution. Record cleaning followed Appendix S4, resulting in 598 spatially unique records (Figure 1). To avoid geographic sampling bias resulting in environmental biases, we carried out spatial thinning of occurrence records to 1 km and sampled 10,000 background points with a probability function that reflected survey effort (Anderson & Raza, 2010; Phillips & Dudík, 2008; Phillips et al., 2009) (details in Appendix S4)

We used MaxEnt 3.4.1 (Phillips et al., 2006, 2017) implemented in SDMtune (Vignali et al., 2020), which uses the principle of maximum entropy to estimate a species’ geographic distribution relative to the environment at occurrence locations (Phillips et al., 2017). To avoid overfitting and enable model validation on independent data sets (Hastie et al., 2009; Phillips & Dudík, 2008), 598 occurrence records were randomly split into 3 subsets (60% for model training, 20% for hyperparameter tuning, and 20% for evaluation and testing). Using a masked geographically structured approach (Radosavljevic & Anderson, 2014), the training data set was split further into 4 cross‐validation folds to account for spatial nonindependence with the checkerboard2 method in ENMeval (Muscarella et al., 2014) (Appendix S4). We evaluated model performance with area under the receiver operating characteristic curve (AUC) (Fielding & Bell, 1997). This approach has been criticized for not identifying the most biologically meaningful models (Warren et al., 2020). To address this, we included variables in our initial model only if we had a priori biological hypotheses for how they influence habitat suitability (Figure 2) and used further steps to reduce collinearity among predictors and model complexity (Appendix S4). This included species‐specific hyperparameter tuning, which is shown to improve model performance by identifying optimal model complexity (Radosavljevic & Anderson, 2014). We mapped the predicted relative probability of northern quoll occurrence by applying cloglog transformation to the raw output of the final model (Phillips et al., 2017).

### Genetic samples

Ear biopsy samples and DNA were sourced from academics, the Western Australian Museum, and DBCA and were collected from 2009 to 2017 (Appendix S5). Tissue samples were collected under relevant ethics guidelines for each institution (approval details in Appendix S5). We extracted DNA with proteinase K digestion, protein salting out, and ethanol precipitation (Miller et al., 1988). We sent ∼350 ng of DNA for 282 samples to Diversity Arrays Technology for single‐nucleotide polymorphism (SNP) genotyping (DArTseq), a proprietary reduced representation method for library preparation and next‐generation sequencing (Kilian et al., 2012) (Appendix S5).

We used a custom R script to filter SNP loci in which visualization was used to determine filtering thresholds. Due to low sequencing yield, 27 individuals were removed. We then filtered on a threshold of 5% missing data per SNP locus, an average total read depth from 20 to 200, 95% reproducibility (based on DArT replicates), and a minimum minor allele frequency of 0.02. We used BLAST (Appendix S5) to remove loci located on sex chromosomes. We then calculated pairwise genotypic correlations (sliding window 500,000 bps) in the R package SNPRelate. We removed SNPs with a correlation ≥0.5 (Zheng et al., 2012). For analyses based on Hardy–Weinberg equilibrium (HWE), we filtered SNPs that significantly deviated from HWE assumptions (with Bonferroni correction) in dartr (Gruber et al., 2018). We also calculated Wang's (2002) pairwise relatedness estimate with the R package related (Pew et al., 2015) and removed 1 of the pair if relatedness was ≥0.25, to avoid biasing population genetic analyses (Wang, 2018).

### Landscape genetics

Panmixia and IBD were tested explicitly or as null models within hypotheses of IBB, IBR, and IBE. Unless otherwise stated, we used the SNP data set without HWE filtering because most analyses made no assumption of HWE.

### Isolation by barrier

We investigated genetic structure across the Pilbara by first thinning the data set to 1 individual per location (194 individuals) to balance sampling. Genetic differences among individuals were visualized using principal coordinate analysis (PCoA) in dartr (Gruber et al., 2018). Next, we used tess3r (Caye & Francois, 2016) to estimate individual ancestry coefficients and determine the number of genetic clusters (*K*) in the Pilbara (Appendix S6). We performed 100 independent runs for *K* = 1–7 with default parameters, withholding 10% of the data for cross‐validation. Optimal *K* was determined by the cross‐entropy criterion, and the run with the lowest root mean squared error was presented.

Admixed individuals (defined by admixture coefficient proportions <0.7) were not included in population genetic summary statistics. Summary diversity statistics were calculated for each genetic cluster in GenAlEx (Peakall & Smouse, 2012) after filtering SNPs for HWE. We estimated genetic diversity across the total data set (filtered for HWE) with the R function GENHET (Coulon, 2010), which calculates the proportion of heterozygous loci for each individual (PHt) (Aparicio et al., 2006). We determined areas of high and low genetic diversity by calculating the mean PHt values across individuals in a 15‐km^2^ area; this distance delineates the genetic neighborhood (Spencer et al., 2013). We identified areas that were 1.5 SDs below (coldspots) or above (hotspots) the mean. Finally, we investigated IBD in populations with Mantel tests implemented in GenAlEx; significance was assessed across 999 permutations (Smouse et al., 1986).

As an extension of IBB analyses, we used generalized dissimilarity modeling (GDM) (Ferrier et al., 2007) in the R package gdm (Fitzpatrick et al., 2020) to determine whether genetic clusters corresponded to particular landscape characteristics acting as physical barriers to dispersal (Fitzpatrick & Keller, 2015). The GDM fitted nonlinear response curves among environmental and genetic variables with I‐spline basis functions (Ferrier et al., 2007). We calculated the mean pairwise Bray–Curtis distance across individuals within a 1‐km^2^ area (to match the pixel size of rasters) in the ecodist package (Goslee & Urban, 2007), to represent genetic dissimilarity. We generated univariate GDMs for hypotheses relating to IBB to identify physical barriers to dispersal (watercourses and aridity) (Figure 2) by coding these as a binary variable. We did not consider univariate models that explained <5% of the deviance.

### Isolation by resistance

We fitted mixed effects models with a maximum likelihood population effects parameterization (Clarke et al., 2002) to determine whether landscape characteristics (substrate, terrain, fire, watercourses, or vegetation) (Figure 2) affect gene flow. We aggregated rasters representing landscape characteristics to 5‐km^2^ resolution because northern quolls can move several kilometers in a night (Oakwood, 2002) (Appendix S2). We then calculated the mean Euclidean genetic distance for individuals in each 5‐km^2^ pixel (excluding Dolphin Island individuals) with ecodist (Goslee & Urban, 2007) because Shirk et al. (2017) found that this metric maximizes accuracy.

Following Peterman et al. (2014), a genetic algorithm was employed to select optimal resistance values (implemented in the package ResistanceGA, details in Appendix S7) (Peterman 2018). We performed optimization on single and composite surfaces (on a maximum of 4 combined surfaces), calculating pairwise effective distances with commute distance (equivalent to CIRCUITSCAPE resistance distance) (Klein & Randić, 1993; McRae et al., 2008), and compared these with model selection. Models were ranked by Akaike's information criterion corrected for small sample size (AIC_c_) (Akaike, 1974). Models within 2 AIC_c_ of the top model were considered equivalent (Burnham & Anderson, 2002). Distance only (IBD) and random (panmixia) models were included in the model selection as alternate or null hypotheses.

We assessed model fit by visually inspecting residuals. To determine whether results were influenced by temporal and spatial distribution of samples, we refitted the model across 1000 bootstrap iterations of randomly subset individuals (including 75% of the total data set) to calculate the percentage of iterations in which each surface was the top‐ranked model (equivalent to model weight, giving an indication of uncertainty) (Burnham & Anderson, 2002). Finally, we used the top‐ranked resistance surface to predict functional connectivity across the Pilbara by creating a cumulative current density map in CIRCUITSCAPE (Anantharaman et al., 2020; McRae et al., 2008). Following Koen et al. (2014), we demarcated an area that extended the Pilbara study region by 20% and randomly placed 100 nodes around the perimeter of this area as source and destination sites for modeling connectivity, which reduced bias associated with edge effects.

### Isolation by environment

We used GDMs to determine whether gene flow was attenuated by environmental factors (IBE). We generated univariate GDMs for a range of environmental variables, testing hypotheses relating to local adaptation (Figure 2), and excluded variables if the deviance explained was <5%. To address multicollinearity, we excluded correlated variables (Spearman's |*r*_s_| > 0.7, variance inflation factor >5), keeping the variable that explained the greatest model deviance. Next, we ran a multivariate GDM, which included geographic distance (IBD). To avoid overfitting, we used permutation‐based backward elimination to remove nonsignificant variables that contributed least to explained deviance (100 permutations) (Ferrier et al., 2007). This process was repeated until all variables made significant (*p* < 0.05) contributions to the explained model deviance. Variable importance in the final, reduced‐variable GDM was determined with 500 permutations.

We used variation partitioning (Borcard et al., 1992) to determine the unique and shared contribution of each variable to the explained model deviance. We visualized multidimensional genetic patterns with the spline functions derived from the final GDM to transform environmental predictors. Transformed predictors were reduced to 3 principal components and mapped, such that areas with high genetic similarity have similar colors.

## RESULTS

### Species distribution modeling

**FIGURE 3:**
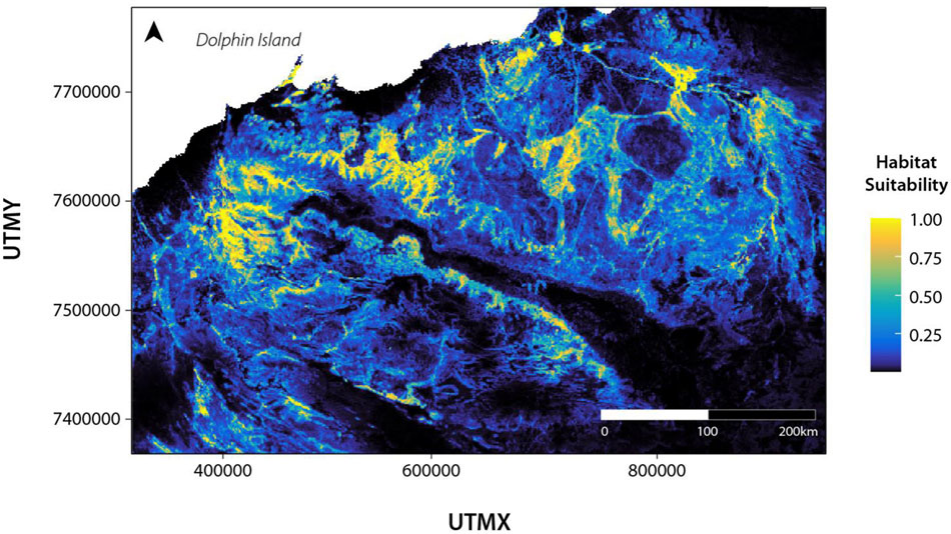
Species distribution model projected across the Pilbara region showing the predicted probability of northern quoll occurrence. A map including occurrence records locations is in Appendix S8.

The MaxEnt SDM (Figure 3; Appendix S8) performed well, with high AUC values for the training (AUC = 0.893) and testing (AUC = 0.874) data sets (Appendix S9). Similar AUC values suggest the model had relatively high accuracy predicting outside the training data set. A total of 12 variables remained in the final model after variable reduction. The variables contributing most to explaining northern quoll occurrence (based on permutation importance) were terrain ruggedness (VRM) (38.7%), elevation (15.7%), Euclidean distance to water (14.6%), precipitation of wettest quarter (7.2%), and precipitation seasonality (B15) (6.3%). The remaining variables made minor (<5%) contributions to the model (maximum temperature of warmest period [B05], spinifex, forest cover, weathering intensity [WII], maximum monthly aridity [ADX], sand, and fire frequency) (Appendix S9).

Response curves (Appendix S10) showed northern quoll occurrence increased then plateaued as terrain ruggedness increased and declined as both elevation and distance to water increased. Occurrence peaked as precipitation of the wettest quarter reached approximately 230 mm. The relationship between occurrence and precipitation seasonality was more erratic, with a somewhat bimodal response.

### Landscape genetics

After SNP filtering, our data set comprised 255 individuals genotyped at 3755 SNP loci. We then removed 36 relatives, resulting in a final data set of 219 individuals (75 females, 121 males, and 23 of unknown sex).

### Isolation by barrier

**FIGURE 4:**
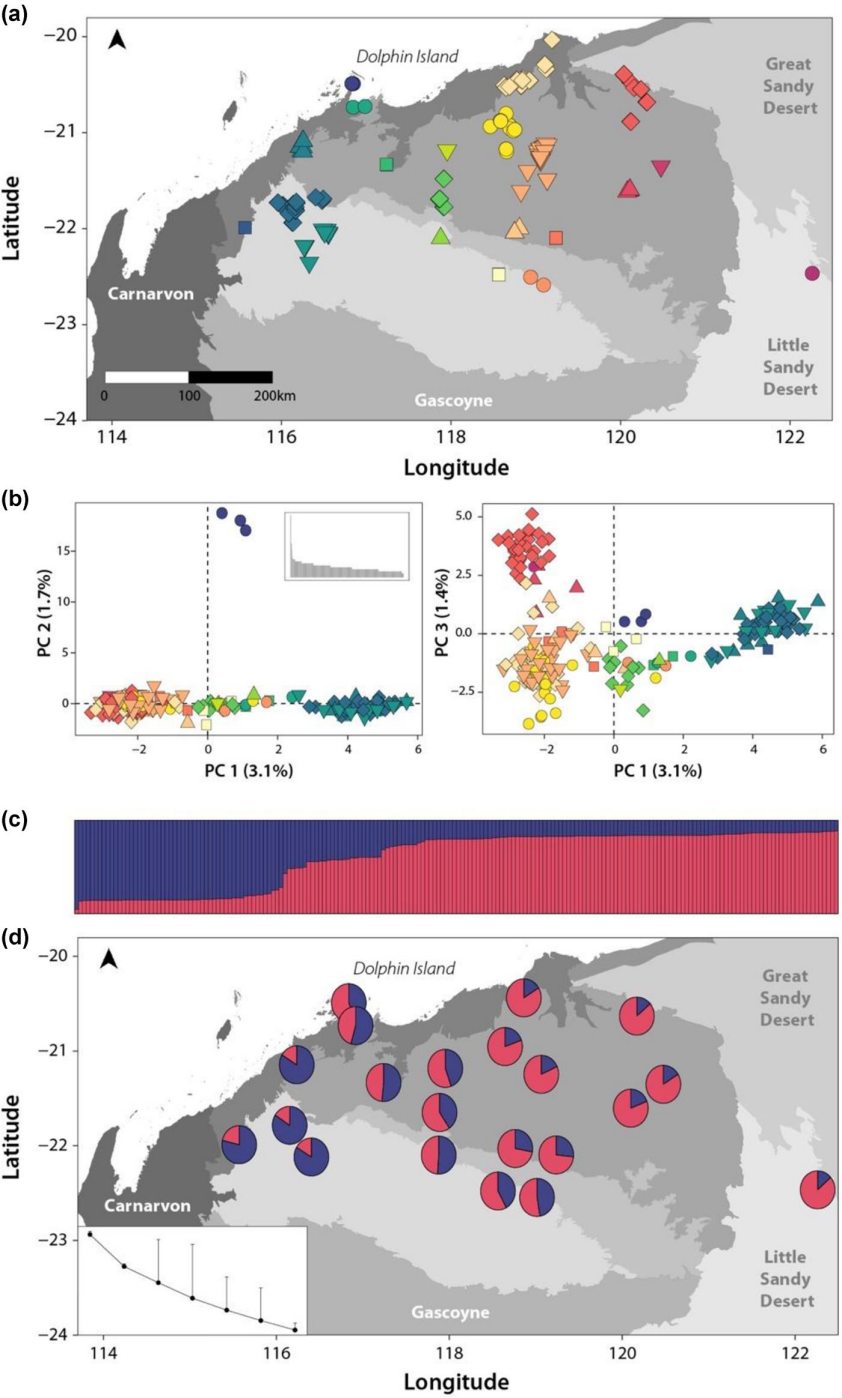
(a) Locations of northern quoll genetic samples in the Pilbara region (matching colors and shapes delineate individual northern quolls grouped in a 15‐km area [i.e., the genetic neighborhood] to provide spatial context for genetic results; gray shading, different IBRA subregions). (b) Comparison of the first 3 principal coordinate axes (PCoA) visualizing genetic differences among individuals (inset, eigenvalues for all PCoA axes). (c) Individual ancestry coefficients for *K* = 2 based on tess3r analysis (purple, cluster 1, southwest; red, cluster 2, northeast). (d) Mean ancestry proportions for *K* = 2 based on tess3r analysis across all individuals in the same genetic neighborhood (inset, cross‐entropy score for *K* = 1−7; error bars, minimum to maximum values)

We found low levels of genetic structure consistent with 2 genetic clusters of northern quolls in the Pilbara, rejecting a null hypothesis of panmixia (Figure 4; Appendix S6). The first 3 axes of the PCoA explained 6.2% of the total variation. Axes 1 and 3 revealed northeastern and southwestern clusters of individuals, with some admixture between these groups. The second axis separated Dolphin Island from mainland individuals (Figure 4). The tess3r analysis (and additional clustering analyses [Appendix S6]) also supported a northeastern–southwestern split, with a broad zone of admixture separating 2 ancestral populations. Univariate GDMs testing IBB hypotheses provided limited evidence that aridity or watercourses acted as barriers to dispersal, explaining <5% of the model deviance.

**FIGURE 5:**
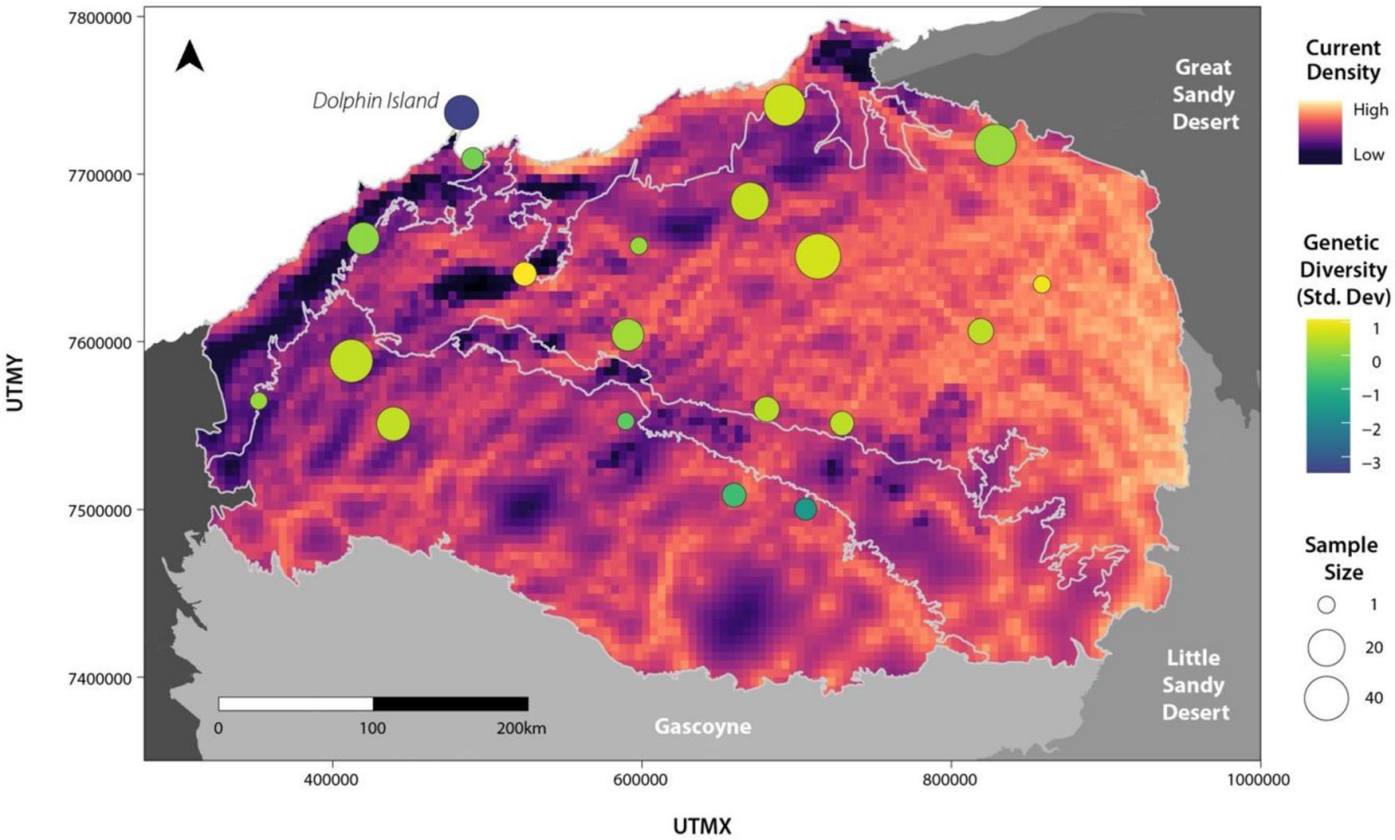
Current density based on the composite resistance surface of distance to water and silt (shows predictions of functional connectivity across the Pilbara landscape) (point color, genetic diversity based on SD of the mean proportion of heterozygous loci for all individuals in a 15‐km area; point size, proportional to sample size; yellow, high levels of genetic diversity relative to the mean; blue, low levels of genetic diversity)

Due to high levels of admixture, 26 individuals were removed from population‐based summary statistics (from the admixture zone, i.e., Dolphin Island and central Pilbara). Heterozygosity estimates were similar between populations (cluster 1: *n* = 53, *H*_o_ = 0.241 [SE 0.003], *H*_e_ = 0.256 [0.003]; cluster 2: *n* = 115, *H*_o_ = 0.244 [0.003], *H*_e_ = 0.259 [0.003]), the inbreeding coefficient was low (*F*_IS_ = 0.050 [0.002]), and genetic differentiation among populations was low (*F*_ST_ = 0.020 [4.309 × 10^–4^]) (Appendix S11). We did not detect any genetic diversity hotspots when the mean PHt was taken across individuals within a 15‐km^2^ area, whereas 2 locations were identified as coldspots (Dolphin Island and 1 Hamersley location) (Figure 5). However, only 2 individuals were present in the Hamersley coldspot. We detected some evidence for IBD, as genetic and geographic distance were positively related, although this relationship was only significant in cluster 2 (cluster 1: *r_xy_* = 0.132, *p* = 0.062; cluster 2: *r_xy_* = 0.266, *p* = 0.001) (Appendix S12).

### Isolation by resistance

According to the model selection (ranked by AIC_c_), the 7 resistance surfaces (and composites) explained more variation in the pairwise genetic data than Euclidian distance (IBD) alone (with the exception of the single *VRM* surface) (Appendix S13). The IBD model outperformed the null model of panmixia (Table 1). There was strong support for the top‐ranked model including a composite surface of distance to water and silt (i.e., substrate) (weight = 0.97, AIC_c_ = 3831.47, ΔAIC_c_ for second‐best model = 8.38, marginal *R* ^2^ = 0.54) (Table 1; Appendix S13). This model ranked first in 95.6% of bootstrap iterations and was stable across 2 replicate runs (Table 1; Appendix S14). Distance to water made a greater contribution to the composite surface than silt (86.75% vs. 13.25%). Specifically, landscape resistance increased as both the distance to water and silt content increased (Figure 5). Due to collinearity, the relationship with silt also meant that resistance increased as clay content increased and sand content decreased. Layer transformations, model estimates, and diagnostic plots are in Appendices S15 and S16.

**TABLE 1:** Model selection* for linear mixed‐effects models of environmental attributes and patterns of genetic structure fit with optimized surfaces (i.e., landscape resistance) as the predictor variable and the Euclidean genetic distance as the response variable and with a population level random effect to account for pairwise comparisons

Surface	Obj.func LL	AIC	AIC_c_	*R* ^2^m	*R* ^2^c	*k*	∆AIC_c_	Weight
Distance to water, silt	−1907.97	3829.94	3831.47	0.54	0.83	7	0	0.97
IBD	−2003.87	4011.75	4011.90	0.51	0.79	2	180.43	0
Panmixia	−3701.47	7404.94	7404.99	0	0.31	1	3573.52	0

* Only the top‐ranked isolation by distance (IBD) and null models are shown for comparison.

Abbreviations: ∆AIC_c_, the difference in AIC_c_ relative to top‐ranked model; AIC, Akaike information criterion; AIC_c_, sample size corrected AIC; *k*, number of parameters; LL, log likelihood; Obj.func, objective function; *R* ^2^c, conditional *R* ^2^; *R* ^2^m, marginal *R* ^2^; weight, AIC_c_ weight.

**TABLE 2:** Model selection results* aggregated over 1000 bootstrap iterations for linear mixed‐effects models of environmental attributes and patterns of genetic structure fit with optimized surfaces (i.e., landscape resistance) as the predictor variable and the Euclidean genetic distance as the response variable and with a population level random effect to account for pairwise comparisons

Surface	Mean LL	Mean RMSE	Mean AIC	Mean AIC_c_	Mean *R* ^2^m	*n*	% top model	Mean rank	Mean weight
Distance to water, silt	−1066.29	0.40	2146.57	2148.73	0.54	956	95.6	1.15	0.92
IBD	−1116.42	0.42	2236.83	2237.04	0.51	0	0	87.94	0

* Only the top‐ranked and isolation by distance (IBD) models are shown for comparison.

Abbreviations: % top model, percentage of iterations where layer of interest identified as top‐ranked model; AIC, Akaike information criterion; AIC_c_, sample size corrected AIC; LL, log likelihood; mean rank, mean ranking across iterations; mean weight, mean AIC_c_ weight across iterations; *n*, number of iterations where layer of interest identified as top‐ranked model; *R* ^2^c, conditional *R* ^2^; *R* ^2^m, marginal *R* ^2^; RMSE, root mean squared error across bootstrap iterations.

### Isolation by environment

**FIGURE 6:**
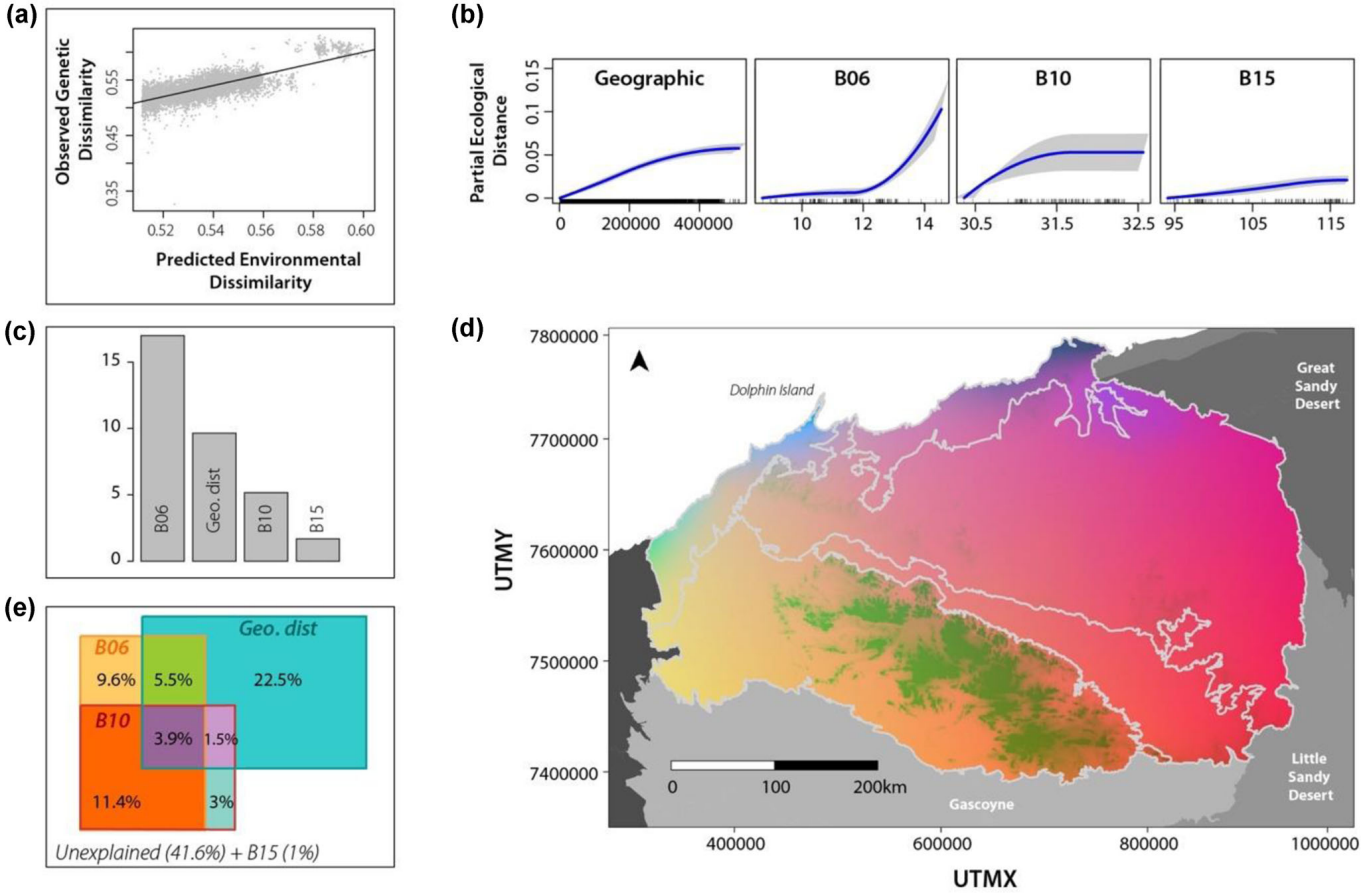
Results of generalized dissimilarity model (GDM) of pairwise genetic distances for northern quolls across the Pilbara: (a) relationship between environmental dissimilarity and genetic dissimilarity, (b) partial contributions of individual variables to variation in genetic distance (B06, minimum temperature during the coldest quarter; B10, mean temperature during the warmest quarter; B15, precipitation seasonality), (c) relative contribution of geographic distance and environmental variables to the multivariate GDM, (d) spatial interpolation of a principal component analysis of GDM‐transformed environmental predictors (the more similar the color the greater the predicted genetic similarity), and (e) variation partitioning identifying the unique and combined contributions of each variable to the explained model deviance (plot boundary, total deviance in genetic turnover; colored boxes, fractions explained by each variable or combination of variables). In panel (e), precipitation seasonality is not included in variation partitioning due to the difficulty of interpreting a 4‐way Venn diagram (percentage explained by this variable alone is included in total deviance).

Univariate GDMs of optimal versus suboptimal habitat (distance to water and terrain ruggedness) explained <5% of the deviance, suggesting local adaptation to these habitat types was not driving genetic turnover in northern quolls. Following variable reduction steps, the final multivariate GDM explained 58.3% of the deviance in genetic turnover in northern quolls across the Pilbara; the relationship between the composite environmental distance and observed genetic distance was linear (Figure 6a). The minimum temperature of the coldest period (B06) was the most important explanatory factor, followed by geographic distance (IBD), mean temperature of the warmest quarter (B10), and precipitation seasonality (B15) (Figure 6b,c). Major gradients in predicted genetic turnover occurred along the coastline (Figure 6d) and within the Hamersley Range. Variation partitioning revealed that geographic distance alone explained 22.5% of the deviance (Figure 6e), and the minimum temperature of the coldest period and the mean temperature of the warmest quarter explained 9.6% and 3% of the deviance in genetic turnover, respectively. An additional 11.4% of the deviance was explained by these variables combined. Precipitation seasonality was not included in variation partitioning due to the low deviance explained by this variable alone (1%) Table 2.

## DISCUSSION

We found that complex, rocky terrain was a crucial component for northern quoll critical habitat and that watercourses facilitated connectivity between habitat patches. There was no evidence for physical barriers to dispersal, although areas with high silt or clay content show higher landscape resistance, and areas with low connectivity tended to show lower genetic diversity. There was a subtle effect of climate on genetic turnover, suggesting local adaptation could arise, though this is unlikely given high levels of gene flow across the Pilbara. The spatial‐genetic maps derived from these findings illuminate dispersal corridors and critical habitat, presenting an opportunity to focus on ground actions, such as targeted threat management, to enhance metapopulation connectivity (Dickson et al., 2019; Klinga et al., 2019). In multiuse landscapes, this information can also inform assessments of potential impacts of future developments and strategies to alleviate, mitigate, or compensate for them. This illustrates the value of incorporating spatial‐genetic data into decision‐making tools and their broad application to threatened species management. In particular, linking life history to genetic findings can have real conservation application (Brüniche‐Olsen et al., 2021).

### Habitat suitability and connectivity

Rocky areas are frequently identified as refugia for vulnerable species due to their thermal, hydric, and pyric characteristics (Reside et al., 2014). Indeed, terrain ruggedness was the best predictor of Pilbara northern quoll distribution, which emphasizes the importance of complex rocky areas in the species’ current core habitat. Evidence across their broader range suggests this is indicative of a recent niche contraction, reflecting the role of habitat complexity in reducing threats (Moore et al., 2019). This may suggest predator avoidance; feral cats frequently use open, flat terrain in the Pilbara (Palmer et al., 2021). Other threats, including habitat degradation by introduced herbivores (Ibbett et al., 2018) and fire (Griffiths & Brook, 2015), are attenuated in rugged and rocky areas as opposed to surrounding grassland. Thus, the impacts of both introduced herbivores and fire are greater on flat, open country than complex rocky areas, enhancing the importance of rocky habitat as a refuge from threats (Moore et al., 2019). In addition, local extinction in quolls is most sensitive to juvenile mortality (Moro et al., 2019), which can be high when mothers leave the den to forage and young are open to predation (Oakwood, 2000). Denning in rugged habitat reduces juvenile predation risk (Cowan et al., 2020), and this habitat type provides protection when juveniles become independent. Thus, rugged terrain drives habitat suitability through increased juvenile survival (and likely increased survival of breeding females), allowing populations to persist through this vulnerable stage.

In contrast with our hypothesis, terrain ruggedness did not facilitate functional connectivity. Hohnen et al. (2016) also found a higher cost to northern quoll movement in rugged terrain in the Kimberley, which they interpreted as a reflection of historical gene flow. However, northern quolls have a short generation time and large dispersal capacity, and we used methods sensitive to subtle patterns of genetic structure (Shirk et al., 2017). Therefore, these results likely reflect contemporary patterns of connectivity, suggesting the differing response to terrain ruggedness (i.e., habitat suitability vs. connectivity) is driven by life history. Although habitat suitability appears to be intrinsically linked to ruggedness by reducing population extinction risk, in contrast, the highly fragmented nature of the rocky habitat in the Pilbara means that quolls have little choice but to move through the matrix of surrounding habitat (Moore, Michael, et al., 2021). Predation risk is higher, but because males are the primary dispersers (Oakwood, 2000) and the species has evolved adaptations (high promiscuity) that compensate for the effects of increased male mortality from annual adult male die‐off (Chan et al., 2020), the impacts on population viability are not as critical. However, management practices that increase survival during dispersal (e.g., reducing wildfire intensity, managing introduced predators) will facilitate metapopulation connectivity.

Distance to water is consistently the top predictor in SDMs for terrestrial animals (Bradie & Leung, 2017), particularly those in arid landscapes (Razgour et al., 2018). This was true for the contemporary distribution of northern quolls (this study; Molloy et al., 2017; Moore et al., 2019), likely due to increased fecundity and survival near watercourses because of a higher abundance and diversity of food (Braithwaite & Griffiths, 1994), as well as the fact that water sources are often found in rocky habitat in the Pilbara. We also found proximity to water facilitated connectivity, likely because dense vegetation along watercourses provides protection from predators during dispersal. Although quolls have some physiological adaptations for dealing with heat stress and water loss (Cooper & Withers, 2010), it appears habitat and microhabitat selection (distance to water and rugged habitat providing cooler dens) are critical for survival, particularly in the Pilbara at the arid extreme of their range. A major concern for the ongoing persistence of northern quolls in the Pilbara is the impending arrival of cane toads to the region (Moore, Dunlop, et al., 2021). The positive relationship between distance to water and northern quoll occurrence suggests a potentially significant overlap in distribution with cane toads (Kearney et al., 2008). As such, efforts to control cane toads are likely to be best targeted to areas associated with water.

### Physical barriers to dispersal and landscape resistance

In general, genetic analyses revealed that northern quolls have high capacity for dispersal with a weak pattern of IBD, a relationship also observed in the Kimberley region (Hohnen et al., 2016). Low genetic structure suggests a lack of major barriers to dispersal within the Pilbara (although surrounding desert likely acts as a major barrier across its broader range; Moore, Dunlop, et al., 2021). A weak northeastern–southwestern split was detected, potentially reflecting historical demography driven by major drainage divides that would have been substantial barriers under wetter climates, a pattern also somewhat detected in geckos (Pepper et al., 2013). This pattern would become weaker as river flows became more intermittent, perhaps explaining why they were not identified as barriers by GDMs. However, the GDMs did detect genetic turnover linked to climate (IBE), showing some patterns consistent with a northeast–southwest split. Therefore, broadscale genetic structure has likely been shaped by both historical and more contemporary processes.

Our results add to the body of work demonstrating the value of individual‐based methods with high sensitivity to contemporary gene flow for understanding the granular drivers of connectivity (Shirk et al., 2017, 2018). We identified higher resistance in areas with high silt or clay content, including alluvial, coastal, and hardpan plains (Figure 5). This pattern is also apparent in northern Australia, where divergent populations of northern quolls are separated by the Carpentaria Gap (a series of claypans that appear to limit dispersal for a range of taxa [Bowman et al., 2010]). Extrapolation of these findings across the landscape revealed high connectivity in the northeast and low connectivity across the Fortescue Marsh, the coastline southwest of Dolphin Island, and in the Hamersley. Some areas with low connectivity also displayed lower than average genetic diversity (e.g., Dolphin Island and east‐Hamersley coldspots), and populations there may be at risk of inbreeding depression and reduced adaptive potential, making them potential targets for conservation intervention in the future (Whiteley et al., 2015).

### Influence of climate on distribution and genetic turnover

Temperature and precipitation are key variables limiting animal distributions and driving adaptation to arid environments (Bradie & Leung, 2017; Razgour et al., 2018). However, climate (maximum summer temperature, mean summer temperature, and rainfall seasonality) was a subtle predictor of habitat suitability and genetic turnover for northern quolls, potentially because rocky habitat buffers quolls from weather extremes. Despite this, a number of genetically unique populations linked to climate gradients were identified (Figure 6), including areas along the coastline buffered to temperature extremes (i.e., higher winter minimums and lower summer averages) but differing in rainfall seasonality (less seasonal in the southwest, more in the northeast). The quolls in central Hammersley were also genetically unique, where temperatures are cool (lower winter minimums and summer averages) and rainfall seasonality is low. The rest of the Pilbara shows a gradient of genetic turnover following high to low rainfall seasonality (northeast to southwest). Patterns were interpolated across poorly sampled areas, and further sampling is required to validate these findings.

### Incorporating landscape genetic and spatial ecology tools in conservation

Combining SDMs with landscape genetics to inform conservation is a recent approach in conservation science that is being used to inform corridor design in fragmented forests (Klinga et al., 2019) and to identify habitat requirements and dispersal behavior in reintroduced populations (Zecherle et al., 2020). Our approach goes further by highlighting the connection between observed patterns and life‐history processes to assist managers in targeting management actions to important components of the northern quoll life cycle. Our results showed different requirements for home range habitat versus connectivity, emphasizing the need for management strategies that protect environmental attributes important for both home ranges and dispersal. This highlights the value of modelling habitat suitability and connectivity separately, especially given that, in some cases, SDMs used directly as resistance surfaces can be poor predictors of connectivity and are outperformed by genetic approaches (Zeller et al., 2018).

We identified landscape features important for targeted conservation management to support northern quolls in the Pilbara. First, it is important to protect large areas of critical rocky habitat, especially female denning habitat, although feral cat control or habitat restoration of surrounding areas could help expand their range (see Palmer et al., 2021). This habitat coincides with areas likely to be mined in the Pilbara, particularly the banded iron formations (Cramer et al., 2016). Although northern quolls will use purpose‐built artificial refuges (Cowan et al., 2020), key habitat patches that facilitate metapopulation connectivity need to be safeguarded and further research is required to understand how to restore habitat after mining (Cowan et al., 2021). Second, drainage lines facilitated connectivity, whereas silt and clay pans impeded dispersal. Silt and clay pans likely have higher predation risk (e.g., feral cats and aerial predators), putting quolls at a disadvantage during dispersal. Effective management of threats to northern quolls, such as feral cats, extensive fire, and overgrazing in and surrounding topographically complex rocky habitat and along watercourses, will help maximize population connectivity and facilitate gene flow, particularly for areas with low genetic diversity (e.g., eastern Hammersley).

The Pilbara is a vast, remote, multiuse region with only 6% protected within the conservation reserve system (Government of Western Australia, 2017). Thus, effective conservation strategies require a landscape‐scale approach, involving cooperation across multiple stakeholders. The spatial products we generated helped identify important landscape features for this species. However, to truly facilitate landscape‐scale conservation, a crucial next step will be interpretation of findings in conjunction with practitioners to inform conservation actions.

### OPEN RESEARCH BADGES

This article has earned Open Data and Open Materials badges. Data and materials are available at https://github.com/RobynSh/NorthernQuolls_LifeHistory2Landscape and https://doi.org/10.5281/zenodo.7005247.

## Supporting information

Additional supporting information may be found in the online version of the article at the publisher's website.
