# Supplementary material for: Linking life history to landscape for threatened species conservation in a multiuse region
Source: Conserv Biol. 2022 Nov 16;37(1):e13989. doi: 10.1111/cobi.13989 (PMC10100189; doi:10.1111/cobi.13989)
Supplement: Supplementary file 1 — Additional supporting information may be found in the online version of the article at the publisher's website. [file COBI-37-0-s001.docx]

**Appendix A**

**Appendix S1. Northern quoll literature review**

Literature review of the environmental and ecological factors influencing how northern quolls use the landscape, and the underlying mechanisms. Findings summarised across the five major regional groupings: KIM = the Kimberley region of Western Australia; LSD = Little Sandy Desert; NT = the Northern Territory; PIL = the Pilbara region of Western Australia; QLD = Queensland.

| **Evidence** | **Mechanism** |
| --- | --- |
| **Climate: Precipitation** | |
| ***Annual precipitation*** – often included in Species Distribution Models (SDMs), but varies in its permutation importance across regions (low importance in the PIL, but high importance in the NT; Moore et al. 2019; von Takach et al. 2020). In the KIM, northern quolls are found in high abundance in areas with intermediate wet season rainfall (Radford et al. 2020), high rainfall rocky savannas (Radford et al. 2014), and landscape genetic models suggest that high rainfall facilitates dispersal (gene flow; Hohnen et al. 2016).  ***Precipitation seasonality*** – identified as an important variable across the northern quoll’s range (Moore et al. 2019).  ***Precipitation of warmest and coldest quarter*** – positively related to northern quoll presence in the PIL (Molloy et al. 2017). | Seasonality and annual rainfall have been linked to survival. For example, one study in the NT found higher survival in the wet season that steadily decreased throughout the dry season (Griffiths & Brook 2015). However, this is likely an indirect link, driven by primary productivity, which in turn influences invertebrate and herbivore populations, and availability of free water, all of which contribute to population growth for mammals (Heiniger et al. 2020). Specifically, plant material and invertebrates (as well as the other small mammals/reptiles that feed on them), make up key components of the northern quoll’s diet (Pollock 1999; Radford 2012; Dunlop et al. 2017). On one NT island, lower rainfall coincided with reduced body weight (and possibly lower juvenile survival), suggesting reduced food availability, while higher wet season rainfall coincided with the greatest female recruitment (possibly due to greater numbers of breeding females or greater juvenile survival; Heiniger et al. 2020). While dietary niche breadth was not correlated with any biogeophysical variables in the PIL (Dunlop et al. 2017), the local availability of food likely fluctuates with climate.  Thus, higher rainfall is linked to greater food availability and higher body condition. This is particularly beneficial for survival for females (Rew-Duffy et al. 2020), as energy requirements likely increase during lactation (Old & Stannard 2020). Juvenile survival is also higher during favourable conditions, which likely influences the spatial distribution of quolls as local extinction risk is most sensitive to changes in juvenile mortality (Peacock & Abbott 2014; Cremona et al. 2017; Moro et al. 2019). |
| **Climate: Temperature** | |
| ***Maximum temperature of warmest month*** – included in northern quoll SDM for the NT, and contemporary samples show a reduction in the realised niche, meaning they have contracted to areas with lower maximum temperatures (compared to pre-2000 samples; von Takach et al. 2020).  ***Minimum temperature of coolest month*** – included in northern quoll SDM for the NT, and contemporary samples show an increase in the realised niche, meaning they now use areas with higher minimum temperatures (compared to pre-2000 samples; von Takach et al. 2020).  ***Mean temperature of driest quarter*** – positively related to northern quoll presence in the PIL (Molloy et al. 2017). | Northern quolls show several adaptations for reducing heat stress. Compared to other quoll species, they have lower body temperatures, the ability to go into torpor, considerable thermolability (which reduces heat gain and the need for evaporative cooling, reducing the energetic costs of thermoregulation; Cooper & Withers 2010). Furthermore, one PIL study showed that den microclimates are buffered against minimum and maximum temperature extremes, as well as fluctuations in humidity (Cowan et al. 2020). This suggests that individuals likely avoid lethal air temperatures, particularly because northern quolls are nocturnal (Hernandez-Santin et al. 2016; Diete et al. 2017; Cowan et al. 2020). Therefore, temperature likely influences northern quoll distributions indirectly, through primary productivity and food availability (as above). |
| **Habitat: Vegetation** | |
| ***General –*** in the KIM, northern quolls occur in most habitats from the coast to inland, and are able to cross mangrove-lined channels (Palmer et al. 2016). Pre-2000 surveying found that northern quolls occupied a wide range of habitats in QLD including vine thickets, forest, woodland, riverine woodland and adjacent to mangroves (Pollock 1999). In the PIL, vegetation (broadly classified into 10 systems based on plains, valleys, plateaus, ranges and hills) had a positive relationship with quoll presence (Molloy et al. 2017). Finally, in the NT northern quolls were more likely to occur where vegetation heterogeneity was low (number of vegetation patches within 1 km; Price et al. 2005).  ***Cover –*** pre-2000, northern quolls in the NT showed no preference for areas with dense understory and were associated with structurally simple, open forest types (Friend & Taylor 1985), while contemporary samples show an increase in the realised niche, meaning they now use areas with greater vegetation cover (von Takach et al. 2020). Likewise, in the PIL more northern quolls were detected at natural den sites that had less bare ground and more spinifex and grass/herb cover compared to artificial den sites (Cowan et al. 2020).  ***Spinifex –*** in the PIL, northern quolls had no response, or a negative response to spinifex habitats (Hernandez-Santin et al. 2016) and rarely utilised hummock grasslands and low lying open sloping plains and floodplains (Henderson 2015). However, northern quolls frequently visited spinifex habitat when associated with shrub steppe and rocky habitat (Henderson 2015).  ***Savanna –*** on KIM islands, northern quolls preferred soil savanna (Gibson & McKenzie 2012).  ***Woodland –*** in a pre-2000 KIM survey, northern quolls were mostly found in woodland (Bradley et al. 1987). However, in the NT, territories in forest or woodland were less likely to be occupied during periods of low population density (Oakwood 2002). In the PIL, northern quoll frequently visited low, sparse spinifex-Eucalypt woodland (Henderson 2015).  ***Rainforest –*** post-2000, northern quolls were associated with rainforest habitat in the KIM (in combination with rocky terrain; Turpin 2015).  ***Riparian –*** positively associated with riparian habitats in some PIL locations (Hernandez-Santin et al. 2016). | Early (pre-2000) studies suggest that northern quolls showed habitat flexibility due to their varied diet (Friend & Taylor 1985). However, more recent studies suggest a greater preference for complex habitat (i.e. greater vegetation cover, including rainforest, woodland, and savanna/hummock grasses associated with complex rocky habitat). This trend may be due to compounding and interacting threats including predation, fire and grazing. For example, in the PIL feral cats and quolls exhibited inverse relationships in their habitat associations in almost all cases (suggesting possible predator avoidance), with feral cats frequently using flat, open habitats (﻿spinifex grasslands, low-density shrubs, non-riparian), while quolls avoided them (Hernandez-Santin et al. 2016). Furthermore, feral cat visitation (and potentially predation) was significantly lower at natural dens versus artificial dens, which had significantly less bare ground (Cowan et al. 2020). Juvenile mortality was highest when left in dens while the mother went to forage (Oakwood 2000), suggesting that increased vegetative cover around dens may have direct consequences for survival. Given that local extinction risk and population survival is most sensitive to juvenile mortality, this vegetation-survival interaction may have broad scale effects on the spatial pattern of northern quolls in the landscape (Cremona et al. 2017; Moro et al. 2019). In this way, grazing by feral herbivores and fire may exacerbate quoll predation by feral cats, as vegetation is often entirely removed, by grazing and trampling, and feral cats are known to frequent areas that are recently burnt (Braithwaite & Griffiths 1994; Hernandez-Santin et al. 2016).  Certain types of vegetation have also been linked to increased growth and survival, by providing higher quality food resources. For example, in the NT, northern quolls were most vulnerable in savanna habitat, where both sexes are more short lived (Braithwaite & Griffiths 1994). Alternatively, the presence of high quality, rocky woodland habitat contributed to high deterministic growth rate (Griffiths et al. 2017). |
| **Habitat: Watercourses** | |
| ***Distance to water –*** a range-wide SDM showed that a reduced distance to water partly describes the northern quoll’s contemporary range, compared to pre-2000 records (permutation importance was similar across regions) (Moore et al. 2019). In the PIL, individuals were frequently located 40 – 540 m from free water (Henderson 2015), and northern quolls showed a positive relationship with distance to water (Molloy et al. 2017), which holds up to 1000 m (becoming negative at 1001-2000 m; Hernandez-Santin et al. 2016) . At the interior extreme of their range (LSD), scats were mostly found within 100 m of water, and waterholes likely act as refuges in otherwise arid environments (Turpin & Bamford 2015). Similarly, in QLD individuals were frequently observed in areas close to permanent water, with over 57% of records within 200 m of permanent freshwater (Pollock 1999). However, in the NT, territories near creeks were less likely to be occupied during periods of low population density, suggesting this was less preferred habitat (Oakwood 2002). | Distance to water has been linked to higher fecundity, better body condition and predator avoidance. In the NT, one study found more breeding individuals, with increased body weight closer to a waterhole (Braithwaite & Griffiths 1994). This is unlikely due to direct water needs (which they likely acquire through food), but rather the availability and diversity of food, which is often most abundant near creek lines (Braithwaite & Griffiths 1994). In the PIL, there were more rugged features closer to rivers (complex habitat providing protection from predators), and both dingoes and feral cats (both known predators of northern quolls) had a negative relationship with water up to 1000 m, though this was likely an anomaly of their modelling approach (Hernandez-Santin et al. 2016). However, the impact of cattle is likely amplified near watercourses, as vegetation is often entirely removed by grazing and trampling within half a kilometre of water (Braithwaite & Griffiths 1994). |
| **Habitat: Substrate/Geology** | |
| ***Sandstone –*** sandstone escarpment is prime northern quoll habitat (Braithwaite & Griffiths 1994). In the KIM, quolls were only detected in sandstone habitat in some locations (Olds et al. 2016, 2017) and this habitat supported the largest, most dense populations (Bradley et al. 1987; Schmitt et al. 1989).  ***Rocky/boulders –*** in some areas in the northern KIM, quolls were only detected in boulder country (Olds et al. 2016, 2017), and large, high density populations occurred on rocky savannas in high rainfall areas (Radford et al. 2014). Furthermore, fractured, rocky terrain (in association with rainforest) provided important habitat (Turpin 2015), with northern quolls sheltering exclusively in rock crevices and caves on KIM islands (Gibson & McKenzie 2012). In the NT, territories in rocky habitat were always occupied (Oakwood 2002). Pilbara northern quolls were positively associated with rocky habitats (Hernandez-Santin et al. 2016) and dens with more embedded rock cover (rock formation fixed to the ground, e.g. inselbergs) had higher detections than artificial dens (with no embedded rock; Cowan et al. 2020). Habitat frequently used by northern quolls in another PIL study included rocky slopes, riverine rocky areas and rocky crests of hills (Henderson 2015). At the arid extreme of their range (LSD) northern quolls are found in rocky gorges, and complex, rocky habitat containing deep rocky crevices or caves (Turpin & Bamford 2015). In QLD, northern quolls were often found near rocky hills, along boulder-strewn slopes (Pollock 1999).  ***Silt, clay, alluvial plains –*** in the PIL, northern quolls rarely utilised low lying sloping open plains and floodplains, especially when in association with silt and clay (Henderson 2015). However, in QLD they were not restricted to rocky areas and were also found in alluvium and plains (Pollock 1999).  ***Granite –*** in QLD, northern quolls were frequently found near granite tors/slabs (Pollock 1999).  ***Metamorphosed rock –*** the habitat most frequently visited by northern quolls in one PIL study was dominated by metamorphosed rock (Henderson 2015). | Sandstone, granite, metamorphosed rock, and rocky/boulder strewn areas in general tend to support the most dense and healthy populations of northern quolls, with animals in rocky country in good condition, having greater longevity, and high deterministic growth rate (Braithwaite & Griffiths 1994; Griffiths et al. 2017). For example, females with rocky habitat within their home range lived longer, and only those with >60% of their dens in rocky habitat survived to a second breeding period in the NT (Oakwood 2000).  This is likely due to both the complexity and diversity of these habitats. Sandstone has a relatively cool and dry microenvironment, and supports a diverse flora (Bradley et al. 1987), which likely provides high availability and diversity of food. The structural complexity of these habitats offers protection from predators. Compared to key predators (feral cats and dogs), quolls are inferior in terrestrial environments but have a stability advantage at higher speeds on narrow supports that are common in rocky habitats (Clemente et al. 2019). In fact, one study found that feral cats were negatively associated with rocky habitats while quolls showed a positive association (Hernandez-Santin et al. 2016). |
| **Habitat: Topography** | |
| ***Elevation –*** elevation was included in a range-wide SDM, but had low variable importance across regions (Moore et al. 2019). In an SDM of the NT, elevation was ranked as the third most important variable, and a decrease in the realised niche for elevation indicates that northern quolls have contracted to areas of lower elevation in the last two decades (von Takach et al. 2020). Elevation also showed a positive relationship with northern quoll presence in a SDM of the PIL (Molloy et al. 2017). However, radio tracking in the PIL suggests that elevation is not necessarily an important factor for habitat selection (Henderson 2015), and QLD northern quolls were recorded across a range of elevations from sea-level to 800 m (median = 60 m, but most at or below 100 m; Pollock 1999).  ***Ruggedness –*** the permutation importance of topographic ruggedness in SDMs varied across regions, but was ranked as the most important variable in the PIL (Moore et al. 2019), showing a positive relationship with northern quoll presence (Molloy et al. 2017). Topographic ruggedness was ranked as the second most important variable in a SDM of the NT (von Takach et al. 2020). While, one radio tracking study in the PIL suggests that terrain ruggedness is not necessarily an important factor for habitat selection (Henderson 2015), ongoing research is showing selection for rocky habitat (unpublished). In contrast, KIM landscape genetics models reveal a higher cost to movement in rugged terrain, suggesting northern quolls may have commonly moved across open habitats in the recent past (Hohnen et al. 2016).  ***Slope –*** terrain slope had a positive relationship with northern quoll presence in SDM of the PIL (Molloy et al. 2017). However, radio tracking in the PIL suggests that slope may not be an important factor for habitat selection (Henderson 2015). | There are no obvious direct links between elevation and northern quoll life history or physiology. However, climate variables and elevation are often closely correlated. Methods used to quantify ruggedness are generally derived from slope and as such, the two are also strongly correlated (Sappington et al. 2007). Thus, slope is unlikely to be an important variable on its own. Terrain ruggedness is used to represent rocky areas. This habitat supports the most dense and healthy populations of northern quoll through increased food resources and protection from predators (see Substrate/Geology above). Finally, areas of high elevation and/or terrain ruggedness are unlikely to be heavily grazed (difficult to access/traverse by introduced herbivores) and may not be as frequently/intensely burnt as hummock and savanna grassland. |
| **Threats: Fire** | |
| ***Fire Frequency (FF) –*** in the KIM, trap success was not related to pre-study FF (Radford 2012). In the NT, the decline of northern quolls may be related to FF (Woinarski et al. 2010), although it had low permutation importance in an SDM of the Top End (von Takach et al. 2020). Stage-structured matrix models predict that an increase in FF of once every 2 years (regardless of extent) cause declines in population size in the NT, while reducing this to once in 5 years reduced extinction risk (Griffiths et al. 2015).  ***Time since last burnt –*** in woodland habitats in the KIM, the greatest abundance was associated with high old growth extent and large patch size (Radford et al. 2020). In the NT, trap success was significantly higher when the area had been largely unburnt for at least 3–5 years, compared to more frequent and pervasive fire in the preceding years (Ibbett et al. 2018). In the PIL, northern quoll were negatively associated with ﻿recently burnt habitats (Hernandez-Santin et al. 2016).  ***Early dry season burning (EDSB)/patch mosaic burning (PMB) –*** in the KIM, abundance increased in sandstone country with patchy EDSB, with the extent of EDSB and interaction between late dry season fire extent and number of late dry season burnt patches important (Radford et al. 2020). Stage-structured matrix models suggest that implementing patch mosaic burning reduced extinction risk in the NT, compared to a regime of fire every two years (Griffiths et al. 2015). | There is evidence of both direct and indirect impact of fire on northern quoll populations. In the NT, female mortality increased after fire, and the number of young leaving the pouch per female decreased (despite an increase in the number of resident females). This suggested fire resulted in delayed breeding (Begg 1981). Another study found no clear effect of fire on survival, but that a late fire treatment resulted in a 20% decrease in recruitment, possibly due to the timing and intensity of fires driving significant mortality of young while in the pouch or den (Griffiths & Brook 2015). Recently burnt areas have been shown to be preferred by feral cats, so fire may result in an increase in predation risk (Hernandez-Santin et al. 2016). However, the interaction between feral cats and fire is still debated in the PIL. |
| **Threats: Infrastructure** | |
| ***Agriculture/mining /urbanisation –*** in QLD, northern quolls were found in urban areas, within sugar cane farms and scats were found on concrete pipes and machinery compacted soils (Pollock 1999). Interactions with mining camps were frequently recorded in the PIL (Henderson 2015).  ***Rail –*** PIL northern quolls were not observed using rail underpasses and there was no evidence they crossed rail lines (Henderson 2015), though more recent work suggests this may not be the case (unpublished).  ***Roads –*** in the PIL, northern quolls frequently recorded making road crossings (Henderson 2015). | ***Agriculture/mining /urban –*** northern quoll are attracted to human food refuse (Pollock 1999). These environments may provide food, but there is no evidence that there is a positive relationship between density/occurrence and urban landscapes, agriculture or mining.  ***Rail –*** rail lines may act as a landscape barrier, with underpasses increasing predation risk by feral cats and dingoes (Henderson 2015).  ***Roads –*** collisions with motor vehicles are a known cause of death (Oakwood 2000). |
| **Species Interactions** | |
| ***Cane toads –*** in the KIM, northern quolls were not recorded in places where cane toads were in high abundance (Turpin 2015). In QLD, there is strong anecdotal evidence of drastic declines after cane toad colonisation. Furthermore, there is moderate spatial overlap in northern quoll and toad habitat (Burnett 1997). However, at one site in the NT there was a substantial decline (88–95%) immediately preceding the arrival of cane toads, so the decline is unlikely to be related to toads alone (Ibbett et al. 2018).  ***Feral cats –*** historical records ﻿highlight the capacity of feral cats to have reduced the distribution and abundance of northern quolls (Peacock & Abbott 2014). In the NT, the high density of northern quolls on one island was attributed in part to the absence of feral cats (Heiniger et al. 2020).  ***Canids –*** dingoes are the top predator in the KIM (Radford et al. 2014). However, they are at low densities in the PIL (Hernandez-Santin et al. 2016), possibly due to ongoing baiting.  ***Snakes –*** black-headed pythons, mulga snakes and olive pythons are known to have caused northern quoll mortality (Pollock 1999; Oakwood 2000).  ***Introduced herbivores –*** cattle are highly likely to have a negative impact on northern quoll presence (Braithwaite & Griffiths 1994). However, in the NT there was a substantial decline at one location (88–95%) where feral stock were largely absent, so the decline is unlikely to be wholly related to introduced herbivores (Ibbett et al. 2018).  ***Parasites –*** parasitism of northern quoll is common (including by lice, *Boopia uncinate*, and ticks, *Haemaphysalis humerosa*; Old & Stannard 2020). | ***Cane toads –*** northern quolls are highly susceptible to mortality through poisoning by cane toad toxin, ingested during predation events (O’Donnell et al. 2010; Ujvari et al. 2013; Jolly et al. 2018). In toad infested areas, northern quolls show no interest in predating toads, whereas northern quolls will often attack cane toads in areas toads have yet to colonise (Kelly & Phillips 2017). Furthermore, in the NT the absence of cane toads contributed to high deterministic growth rate (Griffiths et al. 2017).  ***Feral cats –*** feral cats predate upon northern quolls, causing mortality (Pollock 1999; Oakwood 2000; O’Donnell et al. 2010).  ***Dingoes –*** Dingo predation is a major source of mortality in the NT (Oakwood 2000; Cremona et al. 2017; Jolly et al. 2018). In QLD, domestic farm dogs were responsible for 36% deaths at one site (Oakwood 2000), and are known predators in the NT (Cremona et al. 2017).  ***Snakes –*** snakes are known predators of northern quoll (Pollock 1999; Oakwood 2000).  ***Introduced herbivores –*** introduced herbivores are linked to northern quoll declines through their role in habitat degradation (Ibbett et al. 2018), with grazing and trampling by cattle often entirely removing the vegetation (Braithwaite & Griffiths 1994).  ***Parasites –*** parasitism driven mortality has been suggested, but appears unlikely to be a major factor driving population declines (Oakwood & Spratt 2000). |

**Appendix S2. Raster layers**

|  | **Description and Processing** | | **Plot** |
| --- | --- | --- | --- |
| **General** | All rasters were reprojected to UTM50S, cropped to the smallest raster extent (the light grey area), then resampled to the same pixel size and origin. For the majority of spatial analyses, rasters were aggregated (by the mean value across aggregated pixels) to a 1 km resolution. For Isolation-By-Resistance modelling, rasters were further aggregated to a 5 km resolution. Raster processing was carried out using the R package *raster* (Hijmans 2020). | | 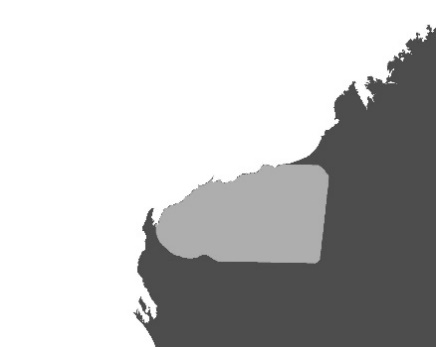  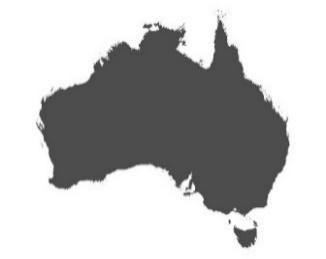 |
|  | **Raster layer** | **Description and Processing** | **Plot** |
| **Climate: Variation** | BPC1 | Principal Component Axis 1 of the BIOCLIM variables (Harwood 2019), using B01 to B33 (excluding crossed variables; i.e. variables that combine temperature and precipitation), generated using the R package *RStoolbox* (Leutner et al. 2019). PC1 explained 96% of the variance in climate across the Pilbara, and the BIOCLIM variables that contributed most to this surface were B12 (annual precipitation = 0.726) and B16 (precipitation of wettest quarter = 0.671). | 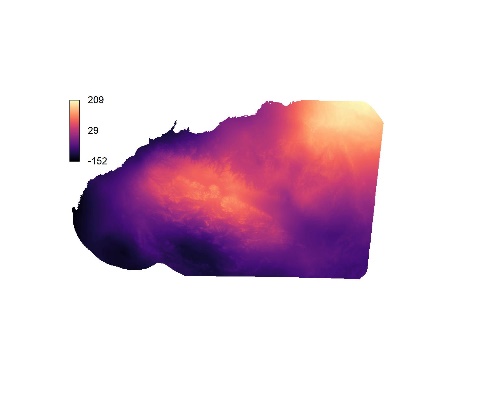 |
|  | BPC2 | Principal Component Axis 2 of the BIOCLIM variables (Harwood 2019), using B01 to B33 (excluding crossed variables; i.e. variables that combine temperature and precipitation), generated using the R package *RStoolbox* (Leutner et al. 2019). PC2 explained 3% of the variance in climate across the Pilbara, and the BIOCLIM variables that contributed most to this surface were B12 (annual precipitation = -0.444), B15 (precipitation seasonality = 0.475), B16 (precipitation of wettest quarter = 0.305), B17 (precipitation of driest quarter = -0.141) and B31 (moisture index seasonality = 0.659). | 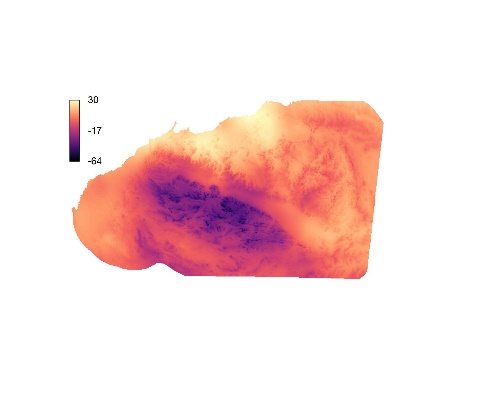 |
|  | BPC3 | Principal Component Axis 3 of the BIOCLIM variables (Harwood 2019), using B01 to B33 (excluding crossed variables; i.e. variables that combine temperature and precipitation), generated using the R package *RStoolbox* (Leutner et al. 2019). PC3 explained 0.6% of the variance in climate across the Pilbara, and the BIOCLIM variables that contributed most to this surface were B06 (minimum temperature of coolest period = 0.220), B07 (temperature annual range = -0.251), B11 (mean temperature of coolest quarter = 0.160), B12 (annual precipitation = 0.335), B13 (precipitation of wettest period = 0.202), B15 (precipitation seasonality = -0.36), B16 (precipitation of wettest quarter = -0.377), B17 (precipitation of driest quarter = -0.377) and B31 (moisture index seasonality = 0.529). | 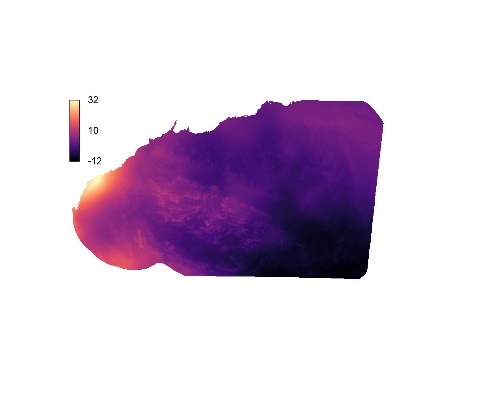 |
|  | BPC4 | Principal Component Axis 4 of the BIOCLIM variables (Harwood 2019), using B01 to B33 (excluding crossed variables; i.e. variables that combine temperature and precipitation), generated using the R package *RStoolbox* (Leutner et al. 2019). PC4 explained 0.2% of the variance in climate across the Pilbara, and the BIOCLIM variables that contributed most to this surface were B01 (annual mean temperature = 0.104), B06 (minimum temperature of coolest period = 0.191), B07 (temperature annual range = -0.172), B11 (mean temperature of coolest quarter = 0.171), B12 (annual precipitation = -0.348), B13 (precipitation of wettest period -0.251), B15 (precipitation seasonality = -0.360), B16 (precipitation of wettest quarter = 0.490), B17 (precipitation of driest quarter = -0.467) and B31 (moisture index seasonality = -0.339). | 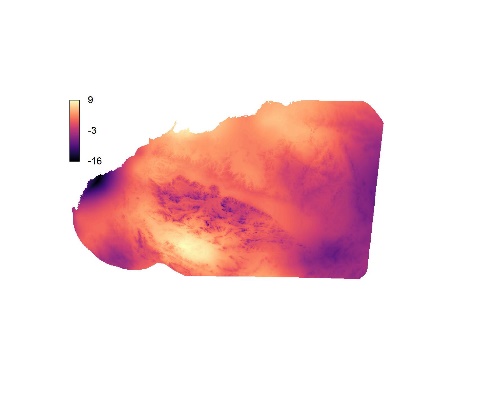 |
| **Climate: Aridity** | ADI (Harwood et al. 2016) | Minimum of monthly aridity index (monthly precipitation/ monthly potential evaporation) – 30 year average centred on 1990 (Xu & Hutchinson 2011). | 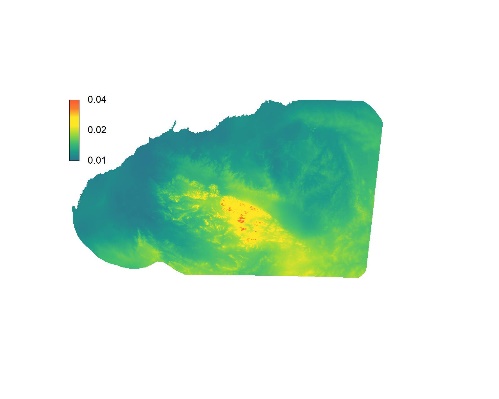 |
|  | ADM (Harwood et al. 2016) | Mean annual aridity index (annual precipitation/ annual potential evaporation) – 30 year average centred on 1990 (Xu & Hutchinson 2011). | 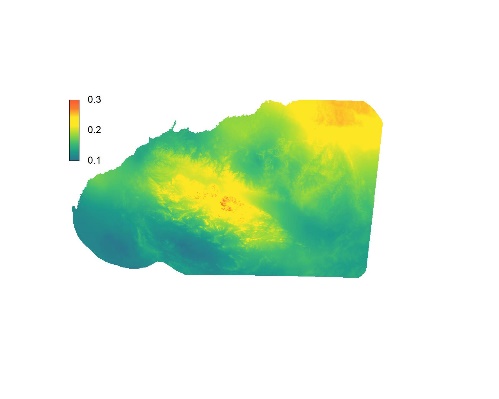 |
|  | ADX (Harwood et al. 2016) | Maximum of monthly aridity index (monthly precipitation/ monthly potential evaporation) – 30 year average centred on 1990 (Xu & Hutchinson 2011). | 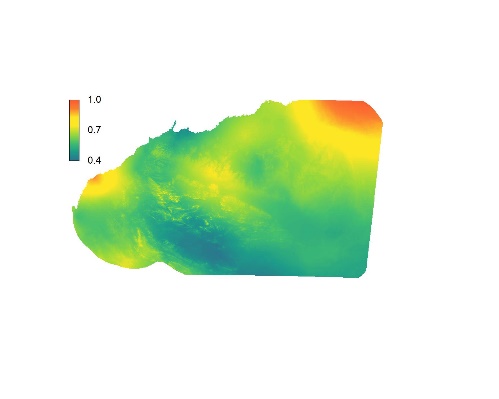 |
| **Climate: Temperature** | B03 (Harwood 2019) | Isothermality – 30 year average centred on 1990. Diurnal temperature range/ temperature annual range (Xu & Hutchinson 2011). | 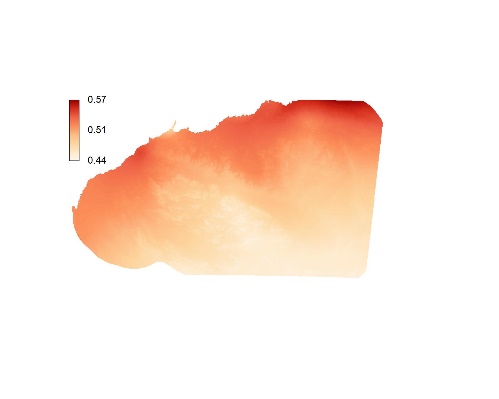 |
|  | B04 (Harwood 2019) | Temperature seasonality (coefficient of variation) – 30 year average centred on 1990. The standard deviation of the weekly  mean temperatures expressed as a percentage of the mean of those temperatures (Xu & Hutchinson 2011). | 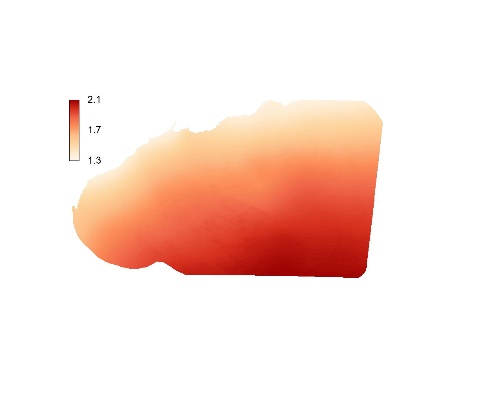 |
|  | B05 (Harwood 2019) | Maximum temperature of the warmest period – 30 year average centred on 1990. The highest maximum temperature in all weeks of the year (Xu & Hutchinson 2011). | 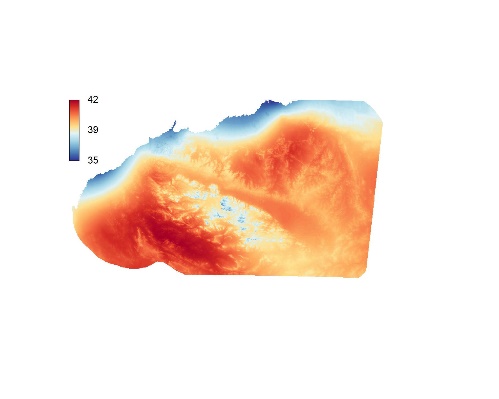 |
|  | B06 (Harwood 2019) | Minimum temperature of the coldest period – 30 year average centred on 1990. The lowest minimum temperature in all weeks of the year (Xu & Hutchinson 2011). | 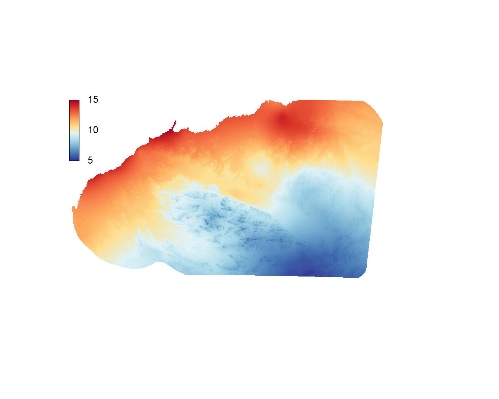 |
|  | B07 (Harwood 2019) | Temperature annual range – 30 year average centred on 1990. The difference between the maximum temperature of the warmest period and the minimum temperature of the coldest period (Xu & Hutchinson 2011). | 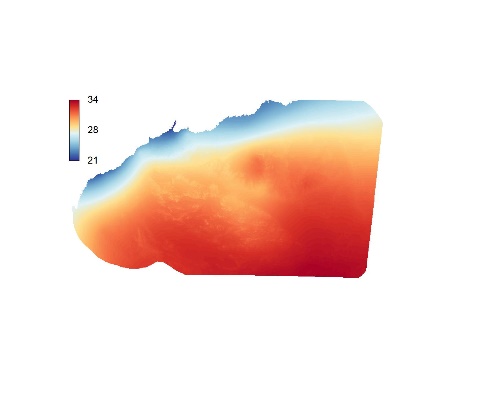 |
|  | B10 (Harwood 2019) | Mean temperature of warmest quarter – 30 year average centred on 1990. The mean temperature of the warmest quarter of the year (Xu & Hutchinson 2011). | 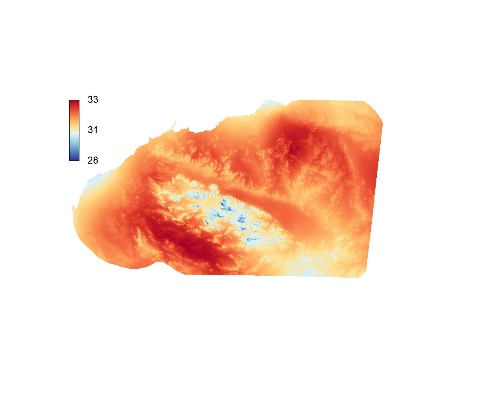 |
|  | B11 (Harwood 2019) | Mean temperature of coldest quarter – 30 year average centred on 1990. The mean temperature of the coldest quarter of the year (Xu & Hutchinson 2011). | 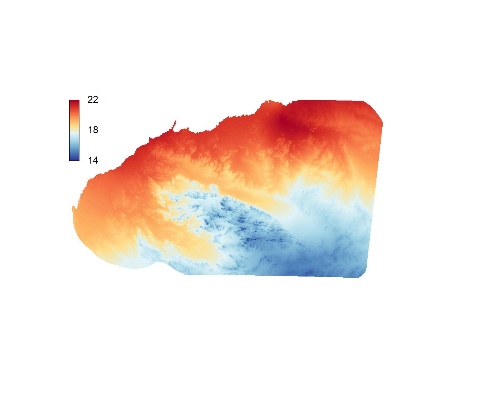 |
| **Climate: Precipitation/Moisture** | B12 (Harwood 2019) | Annual precipitation – 30 year average centred on 1990. The sum of all 12 monthly precipitation estimates (Xu & Hutchinson 2011). | 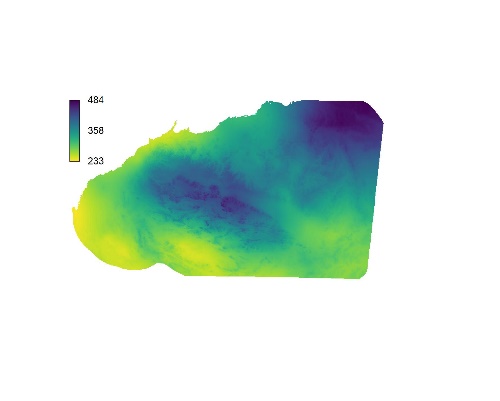 |
|  | B13 (Harwood 2019) | Precipitation of wettest period – 30 year average centred on 1990. The precipitation of the wettest month (Xu & Hutchinson 2011). | 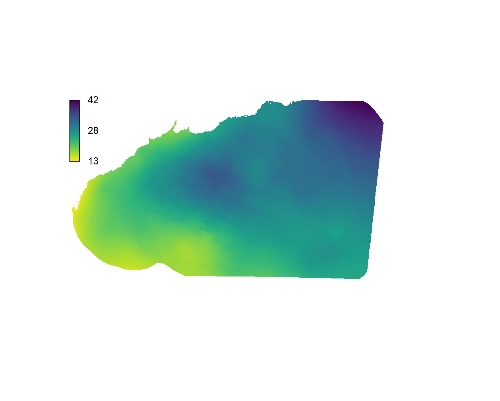 |
|  | B14 (Harwood 2019) | Precipitation of driest period – 30 year average centred on 1990. The precipitation of the driest month (Xu & Hutchinson 2011). | 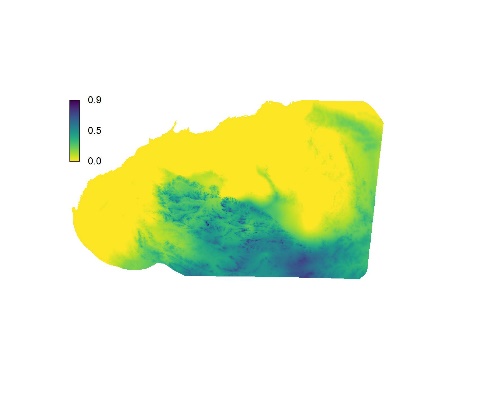 |
|  | B15 (Harwood 2019) | Precipitation seasonality (coefficient of variation) – 30 year average centred on 1990. The standard deviation of the weekly  precipitation estimates expressed as a percentage of the mean of those estimates (Xu & Hutchinson 2011). | 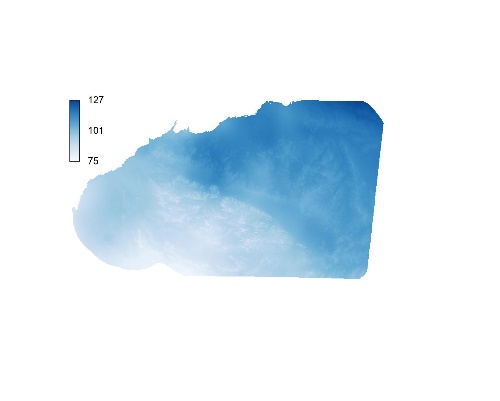 |
|  | B16 (Harwood 2019) | Precipitation of wettest quarter – 30 year average centred on 1990. The total precipitation over the wettest quarter of the year (Xu & Hutchinson 2011). | 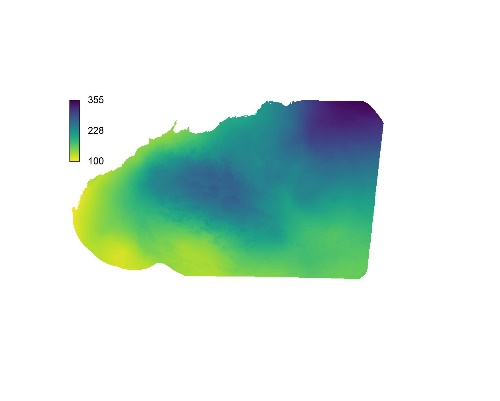 |
|  | B17 (Harwood 2019) | Precipitation of driest quarter – 30 year average centred on 1990. The total precipitation over the driest quarter of the year (Xu & Hutchinson 2011). | 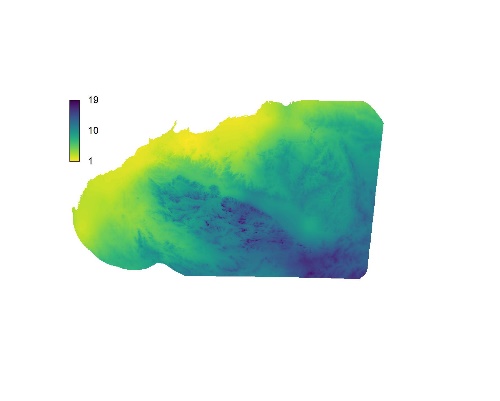 |
|  | B29 (Harwood 2019) | Highest period moisture index (relative soil moisture) – 30 year average centred on 1990. The maximum moisture index value for all weeks of the year (Xu & Hutchinson 2011). | 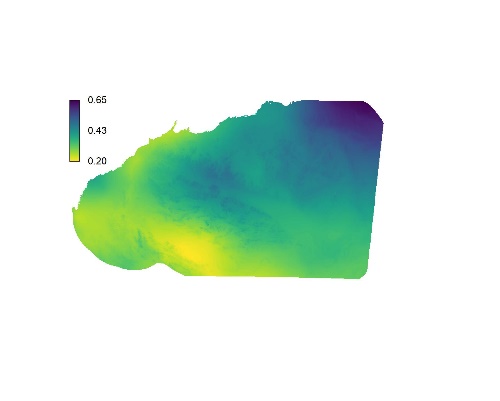 |
|  | B30 (Harwood 2019) | Minimum moisture index value (relative soil moisture) for all weeks – 30 year average centred on 1990. The minimum moisture index value for all weeks of the year (Xu & Hutchinson 2011). | 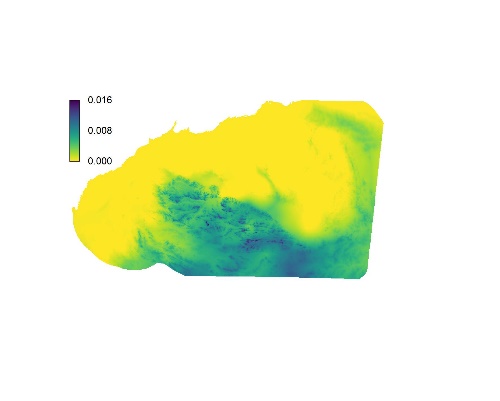 |
|  | B31 (Harwood 2019) | Moisture index (relative soil moisture) seasonality (coefficient of variation) – 30 year average centred on 1990. The standard deviation of the weekly moisture index values expressed as a percentage of the mean of those values (Xu & Hutchinson 2011). | 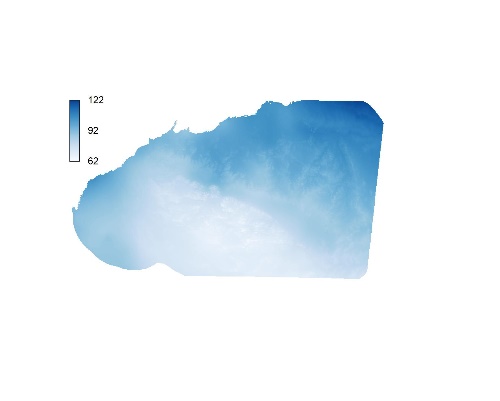 |
|  | B32 (Harwood 2019) | Mean moisture index (relative soil moisture) of wettest quarter – 30 year average centred on 1990. The average moisture index over the quarter of the year with the highest average moisture index value (Xu & Hutchinson 2011). | 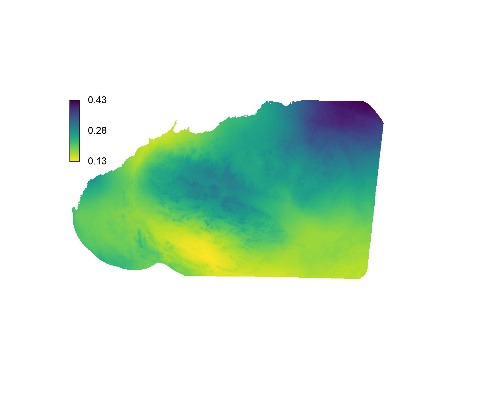 |
|  | B33 (Harwood 2019) | Mean moisture index (relative soil moisture) of driest quarter – 30 year average centred on 1990. The average moisture index over the quarter of the year with the lowest average moisture index value (Xu & Hutchinson 2011). | 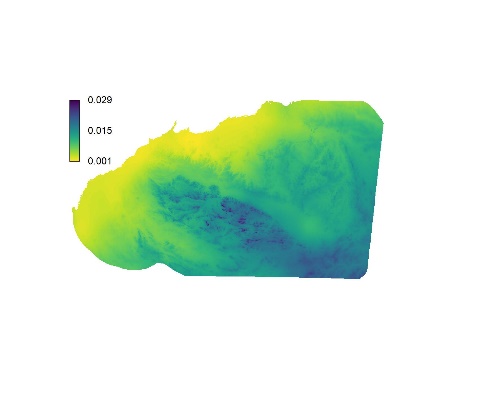 |
| **Substrate/Terrain** | Clay (Holmes et al. 2014) | Layers representing the estimated value (%) of each soil type between 0-5 cm, 5-15 cm, 15-30 cm and 30-60 cm were downloaded using the R package *slga* (O’Brien 2020). The mean percentage was then calculated across raster layers, under the assumption that a depth of 0 – 60 cm would have the greatest influence on animal movement and the ground level vegetation that provides protection from predation and food. | 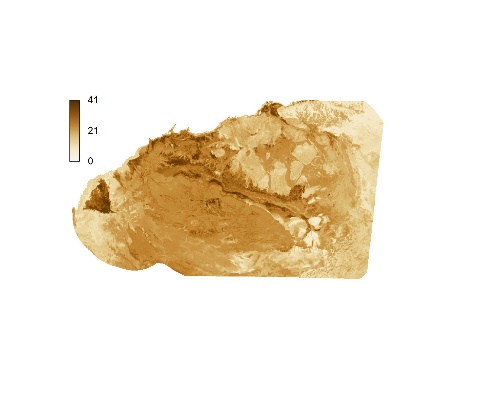 |
|  | Coarse Fragments (Holmes et al. 2014) |  | 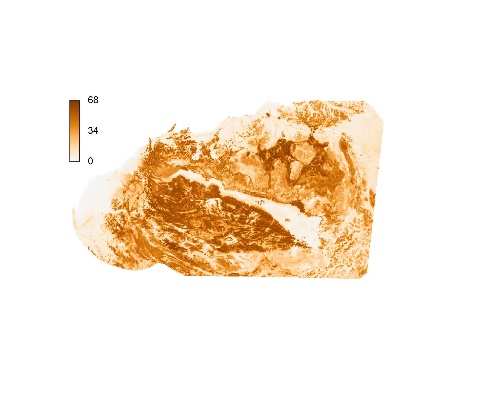 |
|  | Sand (Holmes et al. 2014) |  | 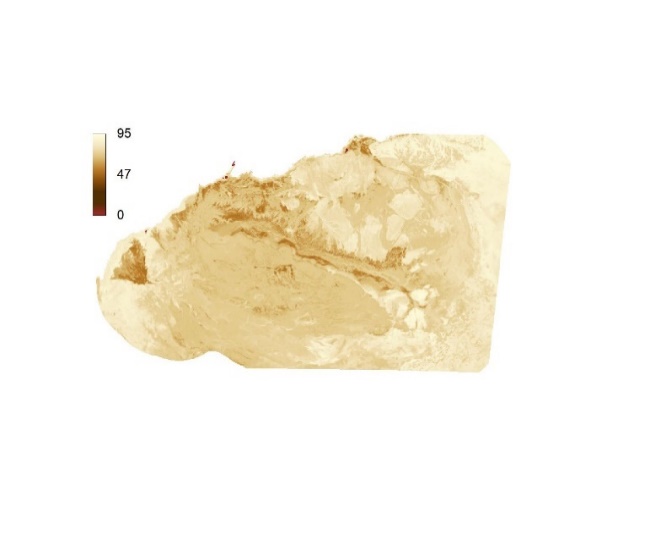 |
|  | Silt (Holmes et al. 2014) |  | 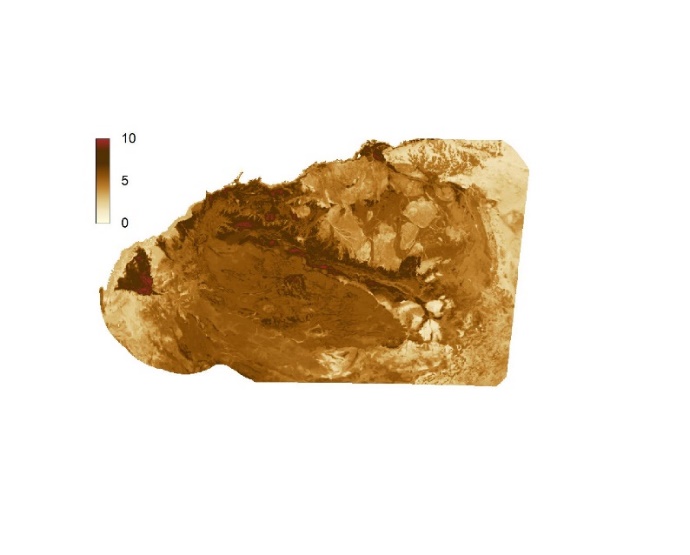 |
|  | Soils (Department of Agriculture and Food WA 2003) | Detailed mapping of Western Australia’s characteristic soils in the Rangelands and Arid interior, categorised into broad soil descriptions based on the proportional allocation of soil groups to mapping units. The 18 soil types were aggregated to five categories, representing soil types most likely to influence quoll movement/occurrence. These included: clay, loam, rocks/gravels, sand, and soils associated with water/high salinity habitat. | 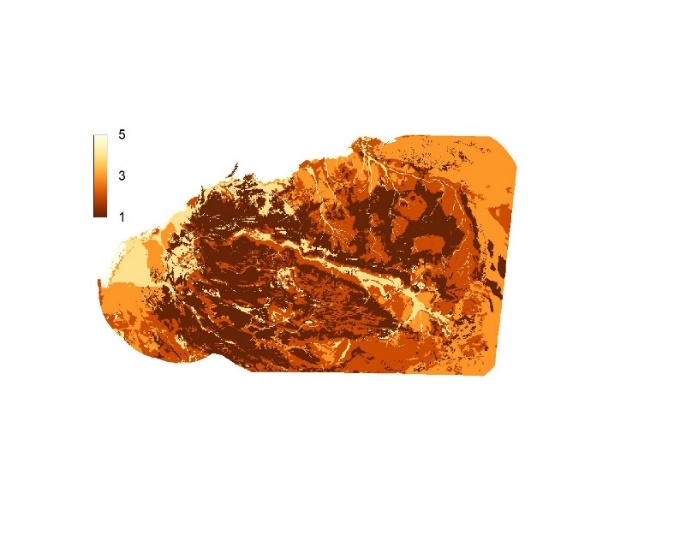 |
|  | Rocky soils | The soil layer (Department of Agriculture and Food WA 2003) was further aggregated to two categories; rocky soils versus non-rocky soils, to represent the preferred habitat of northern quolls. | 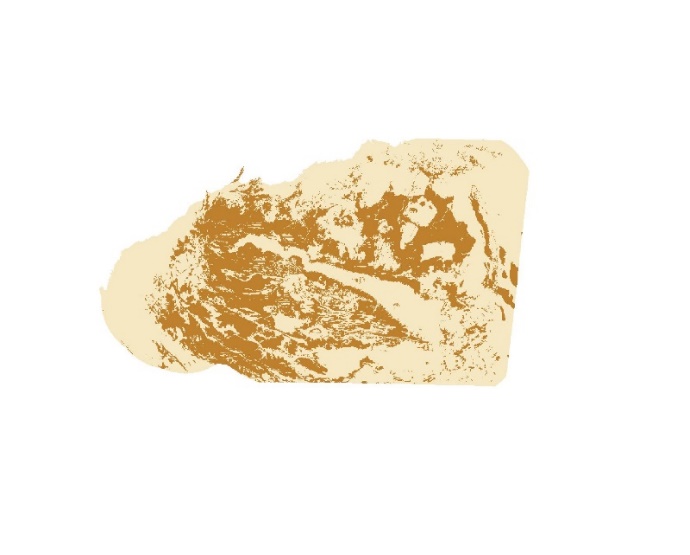 |
|  | WII (Wilford 2012; Wilford & Roberts 2019) | Weathering Intensity Index (WII). Developed using regression models based on airborne gamma-ray spectrometry imagery and the Shuttle Radar Topography Mission (SRTM) elevation data. This index can provide an indication of regolith properties, with 1 representing unweathered bedrock (where outcrops are common), through to 6 representing intensely weathered areas (where clays and sands are common). | 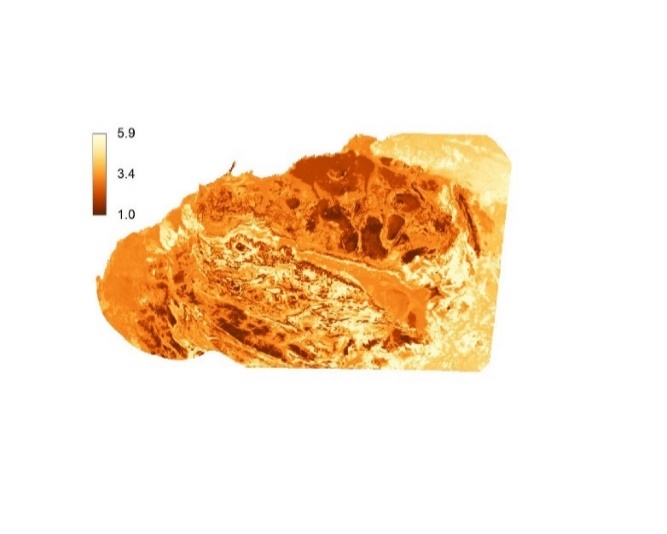 |
|  | VRM | A Vector Ruggedness Measure (VRM) was derived from the DEM (Gallant et al. 2011) using the R package *spatialEco* (Evans 2020). VRM was chosen to represent terrain ruggedness, as it is less correlated with slope than other methods (Sappington et al. 2007; allowing us to test terrain complexity, rather than slope). | 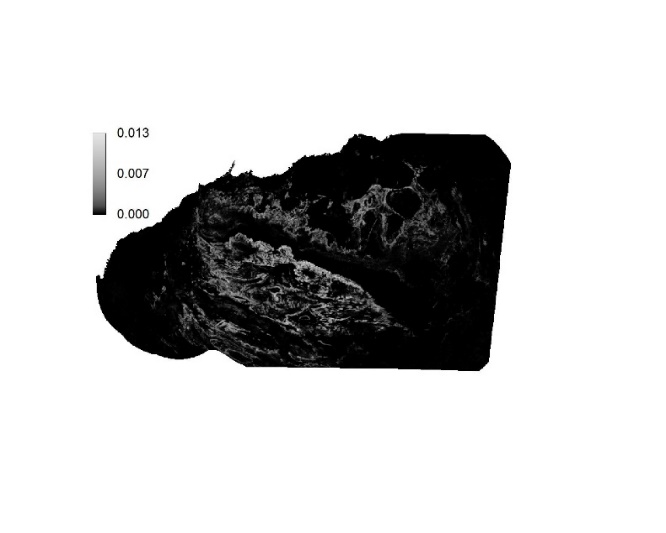 |
|  | VRM.5 | The VRM layer was reclassified to represent “rugged” (the top 5^th^ percentile of ruggedness values in the landscape) versus “flat” terrain (all other values). | 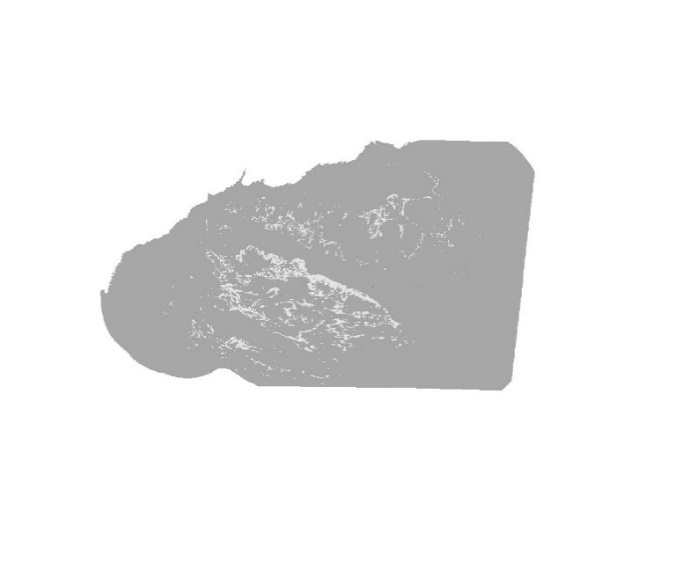 |
|  | VRM.10 | The VRM layer was reclassified to represent “rugged” (the top 10^th^ percentile of ruggedness values in the landscape) versus “flat” terrain (all other values). | 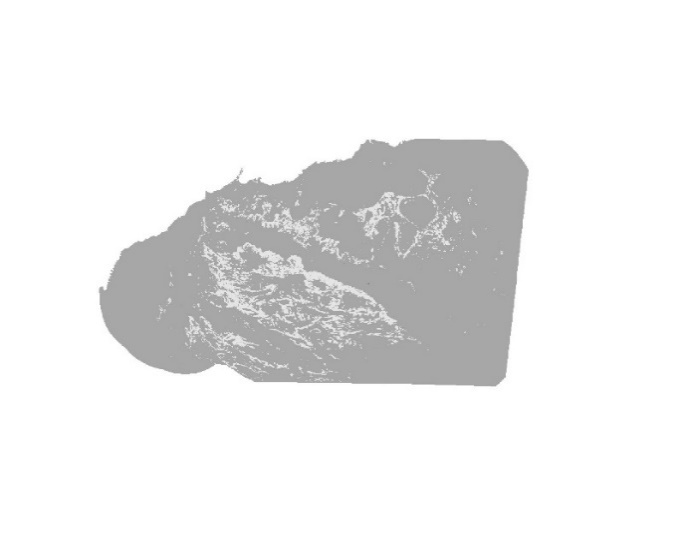 |
|  | VRM.20 | The VRM layer was reclassified to represent “rugged” (the top 20^th^ percentile of ruggedness values in the landscape) versus “flat” terrain (all other values). | 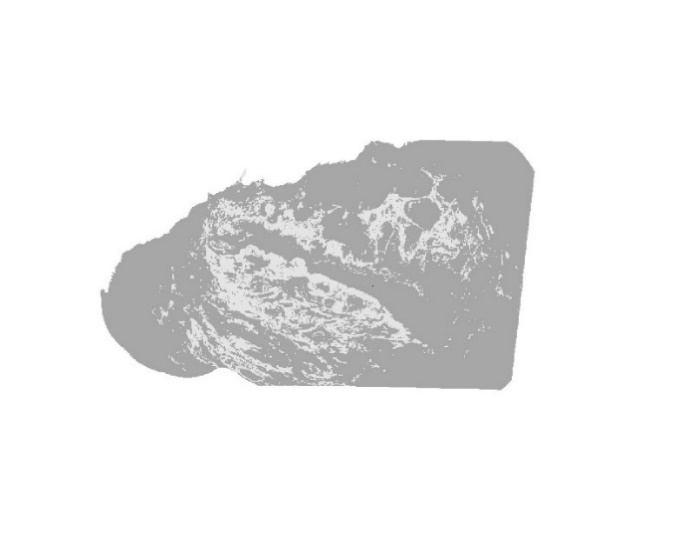 |
| **Elevation** | DEM (Gallant et al. 2011) | Bare-earth Digital Elevation Model, adaptively smoothed to reduce random noise typically associated with the SRTM data in low relief areas. | 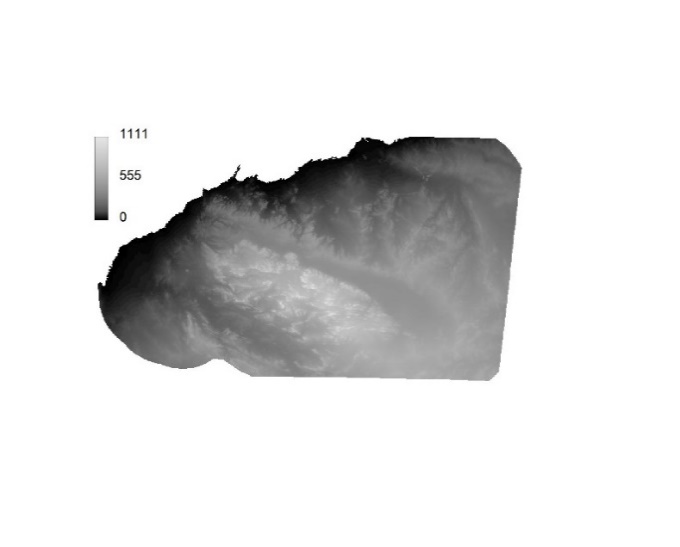 |
| **Watercourses** | Distance to Water | Euclidean distance (m) to natural perennial water (i.e. excluding artificial water points, and inland flats subject to inundation or flooding), derived from Landgate TGDB Hydrology (Landgate 2012, 2017, 2019) in ArcGIS (ESRI 2018). | 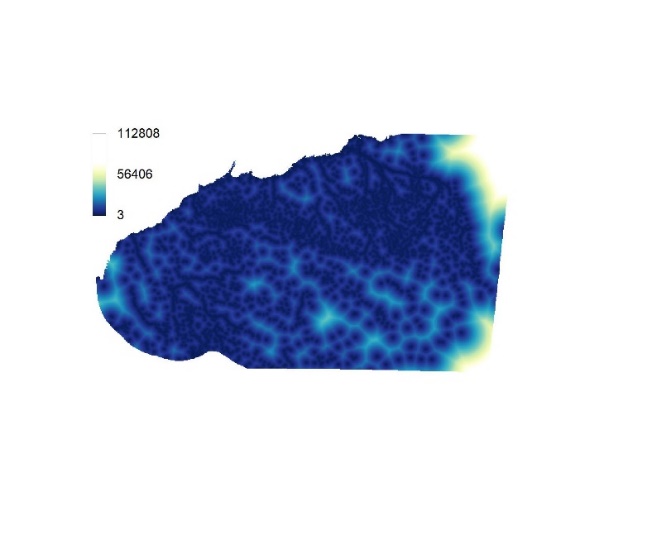 |
|  | Binary 1 km from Water | The Euclidean distance (m) to natural perennial water layer was reclassified to represent “close to water” (areas within 1 km of water), versus “far from water”. | 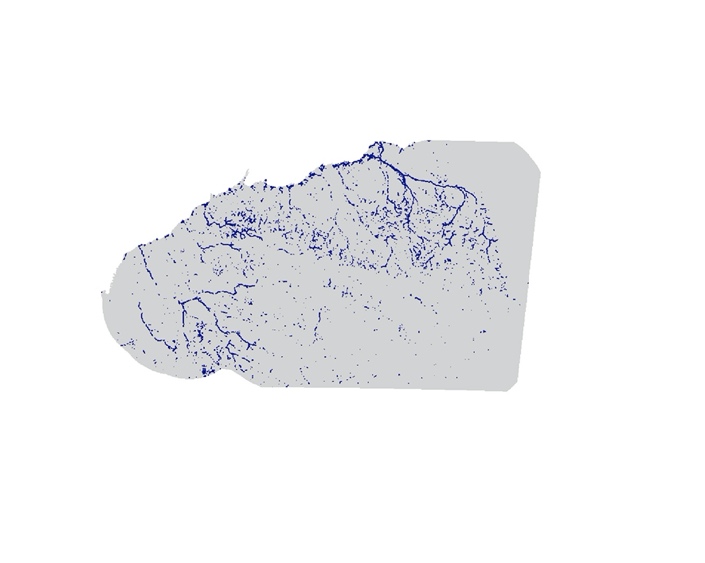 |
|  | Binary 5 km from Water | The Euclidean distance (m) to natural perennial water layer was reclassified to represent “close to water” (areas within 5 km of water), versus “far from water”. | 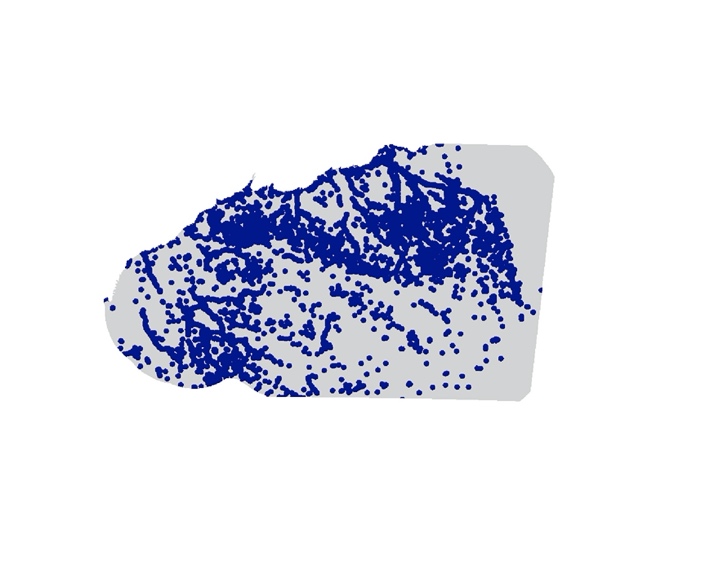 |
|  | Fortescue | The Fortescue river (as a potential landscape barrier). The study extent was split into two categories (north/south) in QGIS (QGIS Development Team 2021), using the Landgate TGDB Hydrology (Landgate 2012, 2017, 2019) derived rasters to define the position of the Fortescue river. | 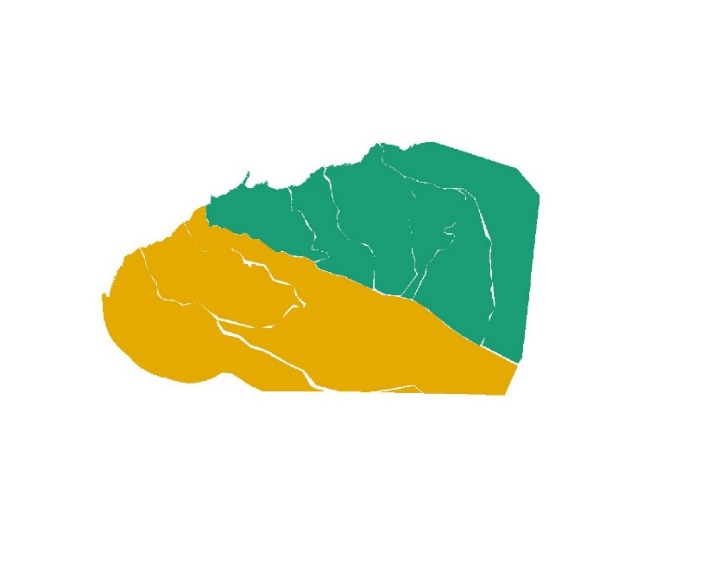 |
|  | Major Rivers | The Fortescue river and the Yule river (as potential landscape barriers). The study extent was split into three categories (south of the Fortescue, north of the Fortescue and east of the Yule, north of the Fortescue and west of the Yule) in QGIS (QGIS Development Team 2021), using the Landgate TGDB Hydrology (Landgate 2012, 2017, 2019) derived rasters to define the position of the Fortescue and Yule rivers. | 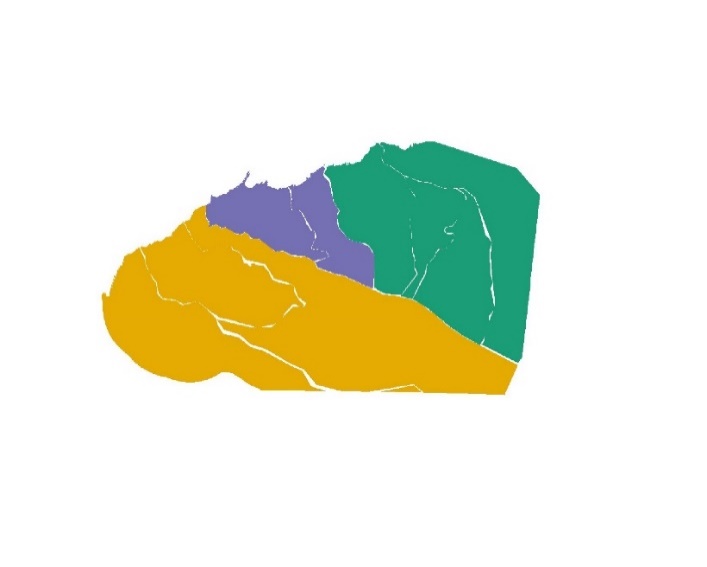 |
|  | River Basins (Geoscience Australia 1997) | Major hydrological basins. Pilbara basin boundaries, as defined by the Australian Water Resources Management Committee. | 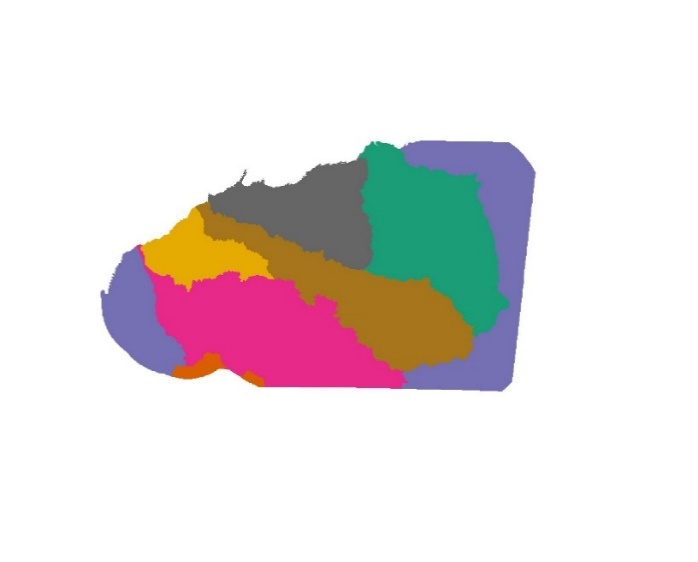 |
| **Vegetation** | Forest Cover | Persistent forest cover. Derived using Land Monitor State-wide products (woody vegetation; Furby et al. 2007; Furby 2018). Frequency data from 23 annual woody layers between 1988 – 2018, including forest cover (>20% canopy cover with expected height at maturity >2m) and sparse woody vegetation cover (5-20% canopy cover). | 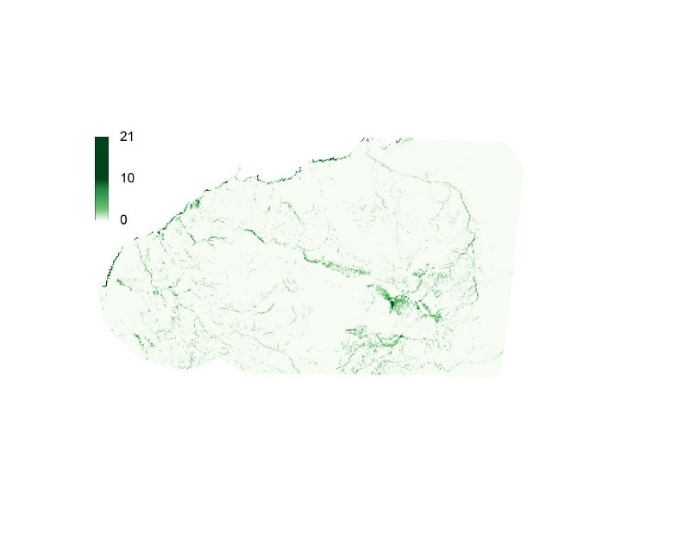 |
|  | Spinifex | Spinifex density index (Rampant et al. 2019) derived using Landsat NBART (Li et al. 2012). A composite of decision rules were applied to determine the likeliest locations of spinifex dominated grasslands in the Pilbara region. | 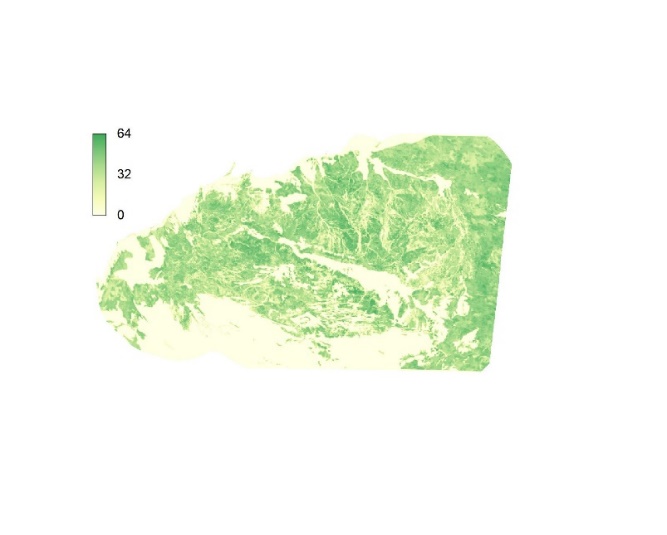 |
| **Threats** | Fire | Fire frequency between 2000 – 2008. Annual fire scar mapping was produced using equivalent methods to NAFI (NAFI 2019). | 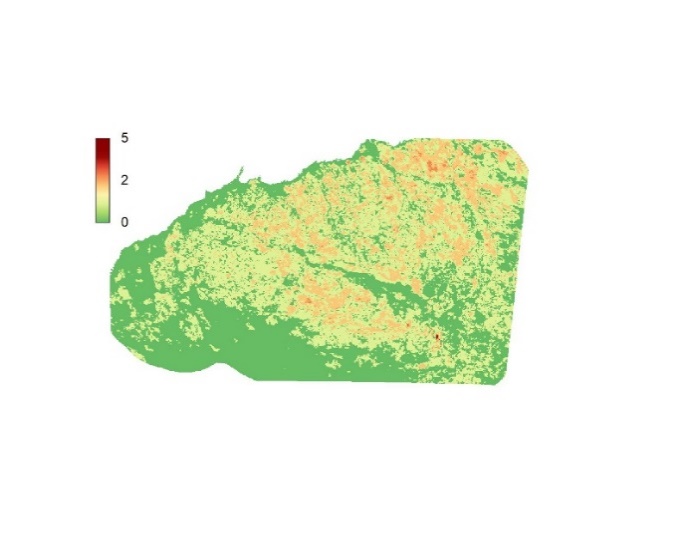 |
|  | Grazing Intensity | **EXCLUDED**  We derived a grazing intensity layer, using pastoral potential upweighted by a factor of 2 if areas were within 1 km of water, and declining to a factor of 1 at 2 km from water (to capture amplified grazing around natural and anthropogenic water sources). Unfortunately, this layer was heavily skewed by missing grazing information from the Little/Great Sandy Desert and had to be excluded. However, within the Pilbara, this layer was strongly correlated with terrain ruggedness, suggesting that rugged areas are less heavily grazed | 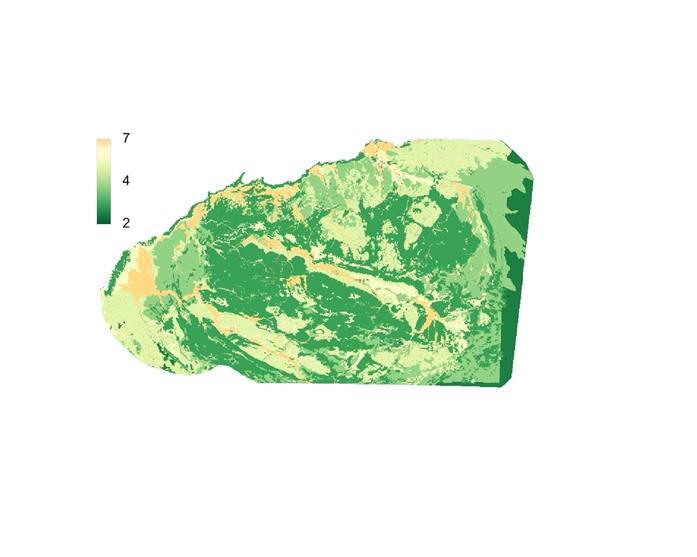 |

**Appendix S3. Correlations between raster layers**

Correlation matrix (Spearman’s r_s_) between environmental rasters tested. Note that only rasters with Spearman’s | r_s_ | ≥0.7 with at least one other raster are presented for ease of visualisation.


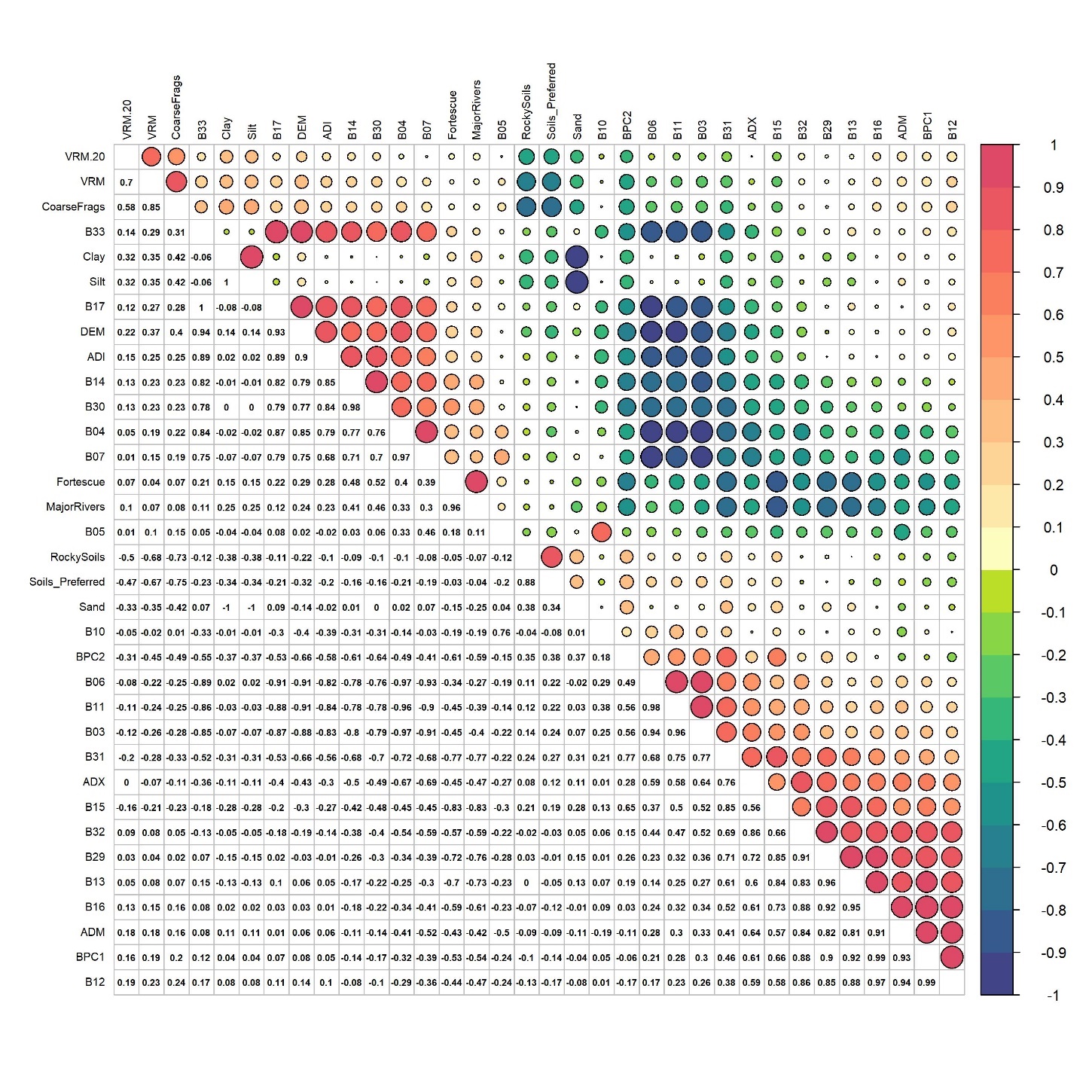


**Appendix S4. Northern quoll Species Distribution Model.**

Raw northern quoll occurrence records were downloaded on the 6^th^ of September 2020 using *ALA4R* (Newman et al. 2019) and the Western Australian Department of Biodiversity, Conservation and Attraction’s (DBCA) NatureMap database (DBCA 2019), and cleaned by excluding records if:

- They fell outside of the Pilbara and surrounding Interim Biogeographic Regionalisation for Australia (IBRA) regions
- Were collected prior to 2000
- Had missing coordinate information
- Had missing or erroneous sample year information
- Had a coordinate accuracy > 1km (those with missing information were validated or excluded) to match the resolution of our spatial data
- Were duplicates (according to museum catalogue numbers)
- Had duplicate coordinates, i.e. we performed spatial thinning (Anderson & Raza 2010) to one record per location even if they were not true duplicates (note that most ALA records were removed at this point)
- Had low species ID certainty (i.e. identified to family or genus only)
- Were based on observation/citizen science (since there were a large number of records, we chose to use those from direct captures to ensure only high-quality records were included)

The records were obtained from the following data providers:

| **Database** | **Data provider** | **Raw record count** | **Clean record count** |
| --- | --- | --- | --- |
| ALA4R | BioCollect - An open cloud-based data collection solution for biodiversity | 8 | 0 |
| ALA4R | Citizen Science - ALA Website | 17 | 0 |
| ALA4R | NatureMap | 144 | 0 |
| ALA4R | Northern Territory Department of Environment and Natural Resources | 7756 | 0 |
| ALA4R | OZCAM (Online Zoological Collections of Australian Museums) Provider | 854 | 1 |
| ALA4R | Questagame | 4 | 0 |
| ALA4R | NA | 754 | 0 |
| NatureMap | BERK_MAMMALS | 31 | 0 |
| NatureMap | COWANSURVEYS | 2 | 1 |
| NatureMap | FAUNASURVEY | 2675 | 1187 |
| NatureMap | ISLAND_MAMMALS | 19 | 0 |
| NatureMap | KI_MAMMALS | 102 | 0 |
| NatureMap | KLCI_FAUNA | 1453 | 0 |
| NatureMap | KLCI_OPPORTUNISTIC | 23 | 0 |
| NatureMap | PILB_ISLAND_SPECIES | 163 | 0 |
| NatureMap | PILBTFAUNA | 1342 | 776 |
| NatureMap | PRS_MAMMALS | 1 | 1 |
| NatureMap | TFAUNA | 540 | 167 |
| NatureMap | WAM_MAMMALS | 285 | 5 |
| NatureMap | WL_REG17 | 1536 | 300 |

Next, post-2000 occurrence records for all Critical Weight Range (CWR) non-volant mammals in the Pilbara were extracted in February 2021 from the NatureMap database (DBCA 2019). Records were cleaned following the procedure described for the northern quoll. To account for sampling effort (Phillips et al. 2009), we followed Molloy et al. (2017) and von Takach et al. (2020) to generate a bias layer by producing a two-dimensional Gaussian kernel density grid (using the default normal reference bandwidth calculation) of CWR occurrence records in the R package MASS (Venables & Ripley 2002). We used this probability function to generate 10,000 background points, ensuring more points were drawn from geographic areas with the greatest survey effort.


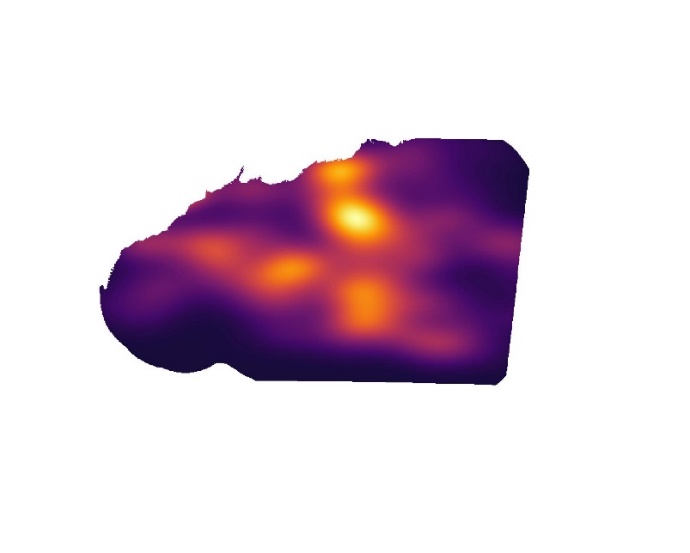


*Critical Weight Range (CWR) mammal sampling bias layer*

Next, we randomly split presence records into three subsets for model training (60%), hyperparameter tuning (20%) and testing (20%), to minimise overfitting and ensure datasets were independent (Phillips & Dudík 2008; Hastie et al. 2009). The training data were then split into four folds based on a checkerboard pattern, to account for spatial non-independence (Radosavljevic & Anderson 2014).


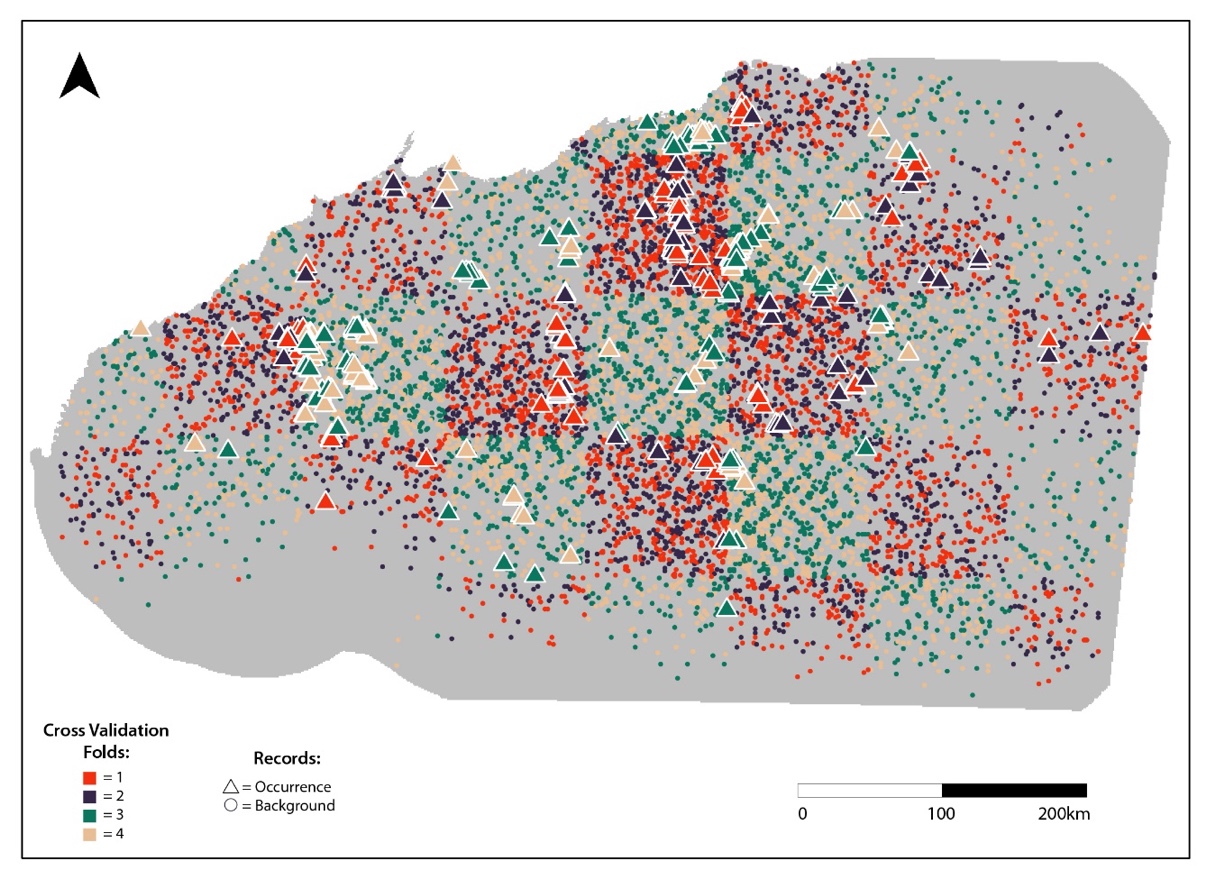


*Training presence and background locations, coloured by cross validation fold*

To generate our MaxEnt model, we followed methods outlined in Vignali et al. (2021) and trained an initial model using default parameters and all variables. Next, to reduce collinearity amongst predictors, a jackknife test removed correlated variables (Spearman’s |rs| > 0.7), keeping those with the highest permutation importance. We then tuned hyperparameters by searching for the best combination resulting in increased model performance across the following variables: feature class (lq, lh, lqp, lqh, lph, lqph, [l= linear, q= quadratic, p= product, h= hinge]), regularization multiplier (0.2 to 5 in increments of 0.2) and number of iterations (300, 500 and 700). With the tuned model, we used the jackknife approach to carry out an additional variable reduction step, removing variables with low permutation importance (<2%) if this did not cause model performance to drop. The final model was trained using the optimised hyperparameters and environmental variables across the training and tuning datasets, while holding back the testing data to for evaluation.

**Appendix S5. Tissue sample information for samples included in final genetic dataset**

Tissue samples were collected over eight years, with the map below demonstrating the spatial-temporal distribution of samples. Outlier sample years clustered with the main sampling years in our genetic clustering analyses, suggesting that genetic results reflect patterns built up over a longer time frame than the eight-year collection window.
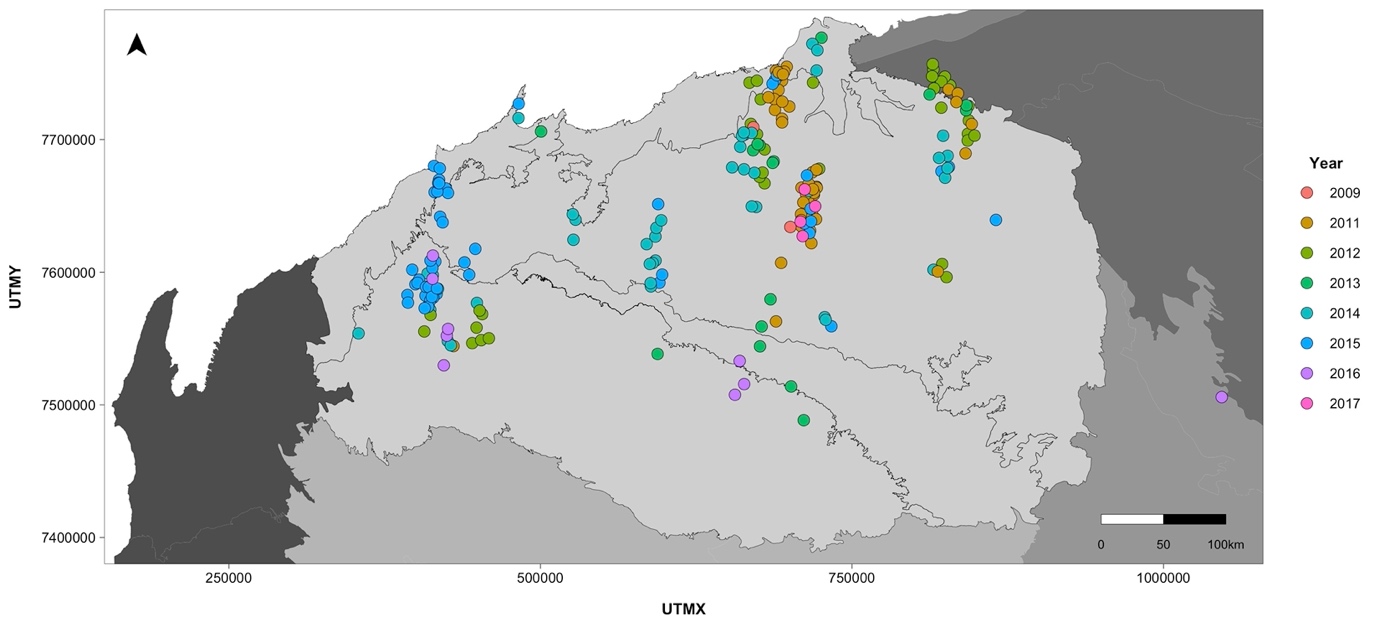


Tissue samples that were sent to Diversity Arrays Technology Pty Ltd for library preparation and next generation sequencing underwent enzyme digestion (PstI and SphI), adapter ligation, PCR amplification, and were sequenced on an Illumina Hiseq2500 at 2.5 million reads per sample. De novo read assembly, quality control and SNP calling were carried out through DArTseqTM proprietary analytical pipelines. Sequences containing SNPs were BLASTed against the Tasmanian devil (*Sarcophilus harrisii*) reference genome (Murchison et al. 2012).

The tissue samples included in the final dataset after filtering and removing related individuals are listed in the table below, where ID = unique ID used in this study, Original ID = ID provided in original database (contact: XXX), WAM ID = ID from the Western Australian Museum catalogue, IBRA = regions defined by the Interim Biogeographic Regionalisation for Australia, Collector = information on sample collector and affiliation. All tissue samples were collected under the DBCA’s Regulation 17 Licence and the relevant ethics guidelines for each institution, including (but not limited to) DEC AEC2009/30, DEC AEC2012/37, DEC AEC2011/14, DEC AEC 2014/19, DBCA AEC 2017/23, and DBCA AEC 2020/21D.

| **ID** | **Original ID** | **WAM ID** | **Sex** | **Year** | **Location** | **IBRA** | **Lat** | **Lon** | **Collector** |
| --- | --- | --- | --- | --- | --- | --- | --- | --- | --- |
| Dh_16.421 | 16-421 | TM0097 | NA | 2009 | Quarry Site 1a | Chichester | -20.5291 | 118.6494 | V. Cartledge and T. Rasmussen, via Judy Dunlop, DBCA |
| Dh_16.426 | 16-426 | TM0100 | NA | 2009 | Quarry Site 1b | Chichester | -21.3994 | 118.9042 | V. Cartledge and T. Rasmussen, via Judy Dunlop, DBCA |
| Dh_11.413 | 11-413 | TM389 | F | 2011 | Abydos Turner Yandee | Chichester | -21.2873 | 119.0561 | WAM sample from Damien Cancilla |
| Dh_12.073 | 12-073 | NA | F | 2011 | Abydos Turner Yandee | Chichester | -21.1653444 | 119.1096944 | NA |
| Dh_12.079 | 12-079 | NA | F | 2011 | Abydos Turner Yandee | Chichester | -21.1544694 | 119.0852972 | NA |
| Dh_12.082 | 12-082 | NA | F | 2011 | Abydos Turner Yandee | Chichester | -21.146625 | 119.0947278 | NA |
| Dh_12.084 | 12-084 | NA | F | 2011 | Abydos Turner Yandee | Chichester | -21.1358861 | 119.1231556 | NA |
| Dh_12.087 | 12-087 | NA | F | 2011 | Abydos Turner Yandee | Chichester | -21.1277083 | 119.1379972 | NA |
| Dh_11.397 | 11-397 | TM396 | M | 2011 | Abydos Turner Yandee | Chichester | -21.1942 | 119.0259 | WAM sample from Damien Cancilla |
| Dh_11.400 | 11-400 | TM399 | M | 2011 | Abydos Turner Yandee | Chichester | -21.1656 | 119.054 | WAM sample from Damien Cancilla |
| Dh_11.401 | 11-401 | TM400 | M | 2011 | Abydos Turner Yandee | Chichester | -21.1742 | 119.0538 | WAM sample from Damien Cancilla |
| Dh_11.402 | 11-402 | TM401 | M | 2011 | Abydos Turner Yandee | Chichester | -21.1656 | 119.1061 | WAM sample from Damien Cancilla |
| Dh_11.405 | 11-405 | TM381 | M | 2011 | Abydos Turner Yandee | Chichester | -21.2124 | 119.0382 | WAM sample from Damien Cancilla |
| Dh_11.409 | 11-409 | TM385 | M | 2011 | Abydos Turner Yandee | Chichester | -21.2499 | 119.0518 | WAM sample from Damien Cancilla |
| Dh_11.411 | 11-411 | TM387 | M | 2011 | Abydos Turner Yandee | Chichester | -21.2285 | 119.0376 | WAM sample from Damien Cancilla |
| Dh_11.412 | 11-412 | TM388 | M | 2011 | Abydos Turner Yandee | Chichester | -21.2729 | 119.054 | WAM sample from Damien Cancilla |
| Dh_12.071 | 12-071 | NA | M | 2011 | Abydos Turner Yandee | Chichester | -21.1653444 | 119.1096944 | NA |
| Dh_12.072 | 12-072 | NA | M | 2011 | Abydos Turner Yandee | Chichester | -21.1653444 | 119.1096944 | NA |
| Dh_12.074 | 12-074 | NA | M | 2011 | Abydos Turner Yandee | Chichester | -21.1653444 | 119.1096944 | NA |
| Dh_12.075 | 12-075 | NA | M | 2011 | Abydos Turner Yandee | Chichester | -21.1653444 | 119.1096944 | NA |
| Dh_12.076 | 12-076 | NA | M | 2011 | Abydos Turner Yandee | Chichester | -21.1653444 | 119.1096944 | NA |
| Dh_12.078 | 12-078 | NA | M | 2011 | Abydos Turner Yandee | Chichester | -21.1653444 | 119.1096944 | NA |
| Dh_12.080 | 12-080 | NA | M | 2011 | Abydos Turner Yandee | Chichester | -21.1544694 | 119.0852972 | NA |
| Dh_2012.077 | 2012-077 | NA | M | 2011 | Abydos Turner Yandee | Chichester | -21.1653444 | 119.1096944 | NA |
| Dh_12.285 | 12-285 | NA | M | 2011 | Callawa | Chichester | -20.6822222 | 120.3105556 | Annette Cook and Julia Lees, DBCA |
| Dh_12.292 | 12-292 | NA | M | 2011 | Callawa | Chichester | -20.6816667 | 120.3116667 | Annette Cook and Julia Lees, DBCA |
| Dh_12.277 | 12-277 | NA | M | 2011 | Cattle Nimingarra Shay | Chichester | -20.5327778 | 120.1594444 | Annette Cook and Julia Lees, DBCA |
| Dh_12.279 | 12-279 | NA | M | 2011 | Cattle Nimingarra Shay | Chichester | -20.5497222 | 120.2408333 | Annette Cook and Julia Lees, DBCA |
| Dh_12.284 | 12-284 | NA | M | 2011 | Cattle Nimingarra Shay | Chichester | -20.5486111 | 120.2402778 | Annette Cook and Julia Lees, DBCA |
| Dh_2012.088 | 2012-088 | NA | F | 2011 | McPhee Creek Mine | Chichester | -21.5930556 | 120.1103056 | NA |
| Dh_11.292 | 11-292 | NA | F | 2011 | Poondano | Chichester | -20.50292001 | 118.8258183 | Chris Jackson, Rapallo Environ. |
| Dh_11.306 | 11-306 | NA | F | 2011 | Poondano | Chichester | -20.45723332 | 118.9309811 | Chris Jackson, Rapallo Environ. |
| Dh_11.307 | 11-307 | NA | F | 2011 | Poondano | Chichester | -20.45468951 | 118.8289203 | Chris Jackson, Rapallo Environ. |
| Dh_11.309 | 11-309 | NA | F | 2011 | Poondano | Chichester | -20.50227942 | 118.8256632 | Chris Jackson, Rapallo Environ. |
| Dh_11.313 | 11-313 | NA | F | 2011 | Poondano | Chichester | -20.48227683 | 118.8310139 | Chris Jackson, Rapallo Environ. |
| Dh_11.314 | 11-314 | NA | F | 2011 | Poondano | Chichester | -20.50821965 | 118.8267037 | Chris Jackson, Rapallo Environ. |
| Dh_11.329 | 11-329 | NA | F | 2011 | Poondano | Chichester | -20.45539576 | 118.8375417 | Chris Jackson, Rapallo Environ. |
| Dh_11.330 | 11-330 | NA | F | 2011 | Poondano | Chichester | -20.45393632 | 118.7790742 | Chris Jackson, Rapallo Environ. |
| Dh_11.333 | 11-333 | NA | F | 2011 | Poondano | Chichester | -20.45497358 | 118.841708 | Chris Jackson, Rapallo Environ. |
| Dh_11.337 | 11-337 | NA | F | 2011 | Poondano | Chichester | -20.50116416 | 118.825765 | Chris Jackson, Rapallo Environ. |
| Dh_11.285 | 11-285 | NA | M | 2011 | Poondano | Chichester | -20.43762213 | 118.8431876 | Chris Jackson, Rapallo Environ. |
| Dh_11.310 | 11-310 | NA | M | 2011 | Poondano | Chichester | -20.5129243 | 118.826508 | Chris Jackson, Rapallo Environ. |
| Dh_11.326 | 11-326 | NA | M | 2011 | Poondano | Chichester | -20.4551211 | 118.9242276 | Chris Jackson, Rapallo Environ. |
| Dh_11.335 | 11-335 | NA | M | 2011 | Poondano | Chichester | -20.45519438 | 118.8260471 | Chris Jackson, Rapallo Environ. |
| Dh_11.338 | 11-338 | NA | M | 2011 | Poondano | Chichester | -20.45524565 | 118.8327215 | Chris Jackson, Rapallo Environ. |
| Dh_12.260 | 12-260 | NA | F | 2011 | Quarry Site 1a | Chichester | -20.5208333 | 118.6891667 | NA |
| Dh_12.296 | 12-296 | NA | M | 2011 | Quarry Site 2c | Chichester | -21.4877778 | 119.1338889 | Annette Cook and Julia Lees, DBCA |
| Dh_12.297 | 12-297 | NA | M | 2011 | Quarry Site 2c | Chichester | -21.4877778 | 119.1336111 | Annette Cook and Julia Lees, DBCA |
| Dh_12.344 | 12-344 | TM589 | M | 2011 | Red Hill Hammersley South | Hamersley | -22.3538889 | 116.3277778 | NA |
| Dh_12.066 | 12-066 | NA | M | 2011 | Tom Price East | Chichester | -21.99548 | 118.8069 | NA |
| Dh_11.404 | 11-404 | TM349 | M | 2011 | Turner River | Chichester | -21.6094 | 118.8253 | WAM sample from Damien Cancilla |
| Dh_12.325 | 12-325 | TM563 | M | 2012 | Abydos Turner Yandee | Chichester | -21.1127222 | 119.1127222 | NA |
| Dh_12.385 | 12-385 | NA | M | 2012 | Callawa | Chichester | -20.6810278 | 120.3066111 | Annette Cook and Julia Lees, DBCA |
| Dh_12.387 | 12-387 | NA | M | 2012 | Callawa | Chichester | -20.6813611 | 120.3073611 | Annette Cook and Julia Lees, DBCA |
| Dh_12.390 | 12-390 | NA | M | 2012 | Callawa | Chichester | -20.6812778 | 120.3085556 | Annette Cook and Julia Lees, DBCA |
| Dh_12.391 | 12-391 | NA | M | 2012 | Callawa | Chichester | -20.6811111 | 120.3066667 | Annette Cook and Julia Lees, DBCA |
| Dh_12.393 | 12-393 | NA | M | 2012 | Callawa | Chichester | -20.6808056 | 120.3085 | Annette Cook and Julia Lees, DBCA |
| Dh_12.395 | 12-395 | NA | F | 2012 | Cattle Nimingarra Shay | McLarty | -20.4738611 | 120.1156389 | Annette Cook and Julia Lees, DBCA |
| Dh_12.394 | 12-394 | NA | M | 2012 | Cattle Nimingarra Shay | McLarty | -20.4729167 | 120.1153056 | Annette Cook and Julia Lees, DBCA |
| Dh_16.441 | 16-441 | TM0901 | NA | 2012 | De grey Station | Roebourne | -20.3388 | 119.1238 | Atlas Iron, via Judy Dunlop, DBCA |
| Dh_14.003 | 14-003 | NA | F | 2012 | Dolphin Island | Roebourne | -20.49007 | 116.84913 | NA |
| Dh_14.006 | 14-006 | NA | F | 2012 | Dolphin Island | Roebourne | -20.49007 | 116.84913 | NA |
| Dh_14.001 | 14-001 | NA | M | 2012 | Dolphin Island | Roebourne | -20.49007 | 116.84913 | NA |
| Dh_14.002 | 14-002 | NA | M | 2012 | Dolphin Island | Roebourne | -20.49007 | 116.84913 | NA |
| Dh_14.004 | 14-004 | NA | M | 2012 | Dolphin Island | Roebourne | -20.49007 | 116.84913 | NA |
| Dh_14.005 | 14-005 | NA | M | 2012 | Dolphin Island | Roebourne | -20.49007 | 116.84913 | NA |
| Dh_12.339 | 12-339 | TM577 | F | 2012 | McPhee Creek Mine | Chichester | -21.5964444 | 120.1111389 | NA |
| Dh_12.331 | 12-331 | TM569 | M | 2012 | McPhee Creek Mine | Chichester | -21.6074722 | 120.0985833 | NA |
| Dh_12.396 | 12-396 | NA | F | 2012 | Nimingarra | McLarty | -20.3910278 | 120.0384444 | Annette Cook and Julia Lees, DBCA |
| Dh_12.397 | 12-397 | NA | M | 2012 | Nimingarra | McLarty | -20.39375 | 120.0359444 | Annette Cook and Julia Lees, DBCA |
| Dh_12.399 | 12-399 | NA | M | 2012 | Nimingarra | McLarty | -20.3939722 | 120.0365278 | Annette Cook and Julia Lees, DBCA |
| Dh_12.400 | 12-400 | NA | M | 2012 | Nimingarra | McLarty | -20.3934722 | 120.0385278 | Annette Cook and Julia Lees, DBCA |
| Dh_12.401 | 12-401 | NA | M | 2012 | Nimingarra | McLarty | -20.3930833 | 120.0385833 | Annette Cook and Julia Lees, DBCA |
| Dh_12.402 | 12-402 | NA | M | 2012 | Nimingarra | McLarty | -20.3926667 | 120.0380278 | Annette Cook and Julia Lees, DBCA |
| Dh_16.263 | 16-263 | NA | F | 2012 | Pannawonica Red Hill | Hamersley | -22.039309 | 116.553333 | Ryan Ellis, Phoenix Environmental, via Judy Dunlop, DBCA |
| Dh_16.257 | 16-257 | NA | M | 2012 | Pannawonica Red Hill | Hamersley | -22.011 | 116.532 | Ryan Ellis, Phoenix Environmental, via Judy Dunlop, DBCA |
| Dh_16.260 | 16-260 | NA | M | 2012 | Pannawonica Red Hill | Hamersley | -22.01012 | 116.48648 | Ryan Ellis, Phoenix Environmental, via Judy Dunlop, DBCA |
| Dh_16.261 | 16-261 | NA | M | 2012 | Pannawonica Red Hill | Hamersley | -22.052558 | 116.553632 | Ryan Ellis, Phoenix Environmental, via Judy Dunlop, DBCA |
| Dh_16.262 | 16-262 | NA | M | 2012 | Pannawonica Red Hill | Hamersley | -22.052558 | 116.553632 | Ryan Ellis, Phoenix Environmental, via Judy Dunlop, DBCA |
| Dh_16.266 | 16-266 | NA | M | 2012 | Pannawonica Red Hill | Hamersley | -22.037695 | 116.509284 | Ryan Ellis, Phoenix Environmental, via Judy Dunlop, DBCA |
| Dh_16.352 | 16-352 | NA | F | 2012 | Quarry Site 1a | Chichester | -20.50552 | 118.628566 | George Watson, BHP BIO, via Annette Cook and Judy Dunlop, DBCA |
| Dh_16.353 | 16-353 | NA | M | 2012 | Quarry Site 1a | Chichester | -20.5296 | 118.649695 | George Watson, BHP BIO, via Annette Cook and Judy Dunlop, DBCA |
| Dh_16.355 | 16-355 | NA | M | 2012 | Quarry Site 1a | Chichester | -20.5296 | 118.649695 | George Watson, BHP BIO, via Annette Cook and Judy Dunlop, DBCA |
| Dh_12.404 | 12-404 | NA | F | 2012 | Quarry Site 2a | Chichester | -20.9200556 | 118.6858611 | Annette Cook and Julia Lees, DBCA |
| Dh_12.403 | 12-403 | NA | M | 2012 | Quarry Site 2a | Chichester | -20.9193333 | 118.6853056 | Annette Cook and Julia Lees, DBCA |
| Dh_12.406 | 12-406 | NA | M | 2012 | Quarry Site 2a | Chichester | -20.9201389 | 118.6841944 | Annette Cook and Julia Lees, DBCA |
| Dh_12.407 | 12-407 | NA | M | 2012 | Quarry Site 2a | Chichester | -20.9194444 | 118.6831944 | Annette Cook and Julia Lees, DBCA |
| Dh_12.410 | 12-410 | NA | M | 2012 | Quarry Site 2a | Chichester | -20.9574444 | 118.6961111 | Annette Cook and Julia Lees, DBCA |
| Dh_16.554 | 16-554 | TM0826 | NA | 2012 | Red Hill Hammersley North | Hamersley | -21.93667 | 116.12767 | C. Jackson, via Judy Dunlop, DBCA |
| Dh_16.555 | 16-555 | TM0835 | NA | 2012 | Red Hill Hammersley North | Hamersley | -21.93767 | 116.13733 | C. Jackson, via Judy Dunlop, DBCA |
| Dh_16.203 | 16-203 | NA | F | 2013 | Callawa | Chichester | -20.68071 | 120.30811 | Judy Dunlop, DBCA |
| Dh_16.200 | 16-200 | NA | M | 2013 | Callawa | Chichester | -20.68144 | 120.30688 | Judy Dunlop, DBCA |
| Dh_14.010 | 14-010 | NA | F | 2013 | Dolphin Island | Roebourne | -20.49007 | 116.84913 | NA |
| Dh_14.012 | 14-012 | NA | F | 2013 | Dolphin Island | Roebourne | -20.49007 | 116.84913 | NA |
| Dh_14.013 | 14-013 | NA | F | 2013 | Dolphin Island | Roebourne | -20.49007 | 116.84913 | NA |
| Dh_14.007 | 14-007 | NA | M | 2013 | Dolphin Island | Roebourne | -20.49007 | 116.84913 | NA |
| Dh_14.009 | 14-009 | NA | M | 2013 | Dolphin Island | Roebourne | -20.49007 | 116.84913 | NA |
| Dh_14.011 | 14-011 | NA | M | 2013 | Dolphin Island | Roebourne | -20.49007 | 116.84913 | NA |
| Dh_16.450 | 16-450 | TM0910 | NA | 2013 | Indee Quarry 2 | Chichester | -20.80153 | 118.64848 | S. A. Thompson, via Judy Dunlop, DBCA |
| Dh_16.453 | 16-453 | TM0913 | NA | 2013 | Indee Quarry 2 | Chichester | -20.80153 | 118.64848 | S. A. Thompson, via Judy Dunlop, DBCA |
| Dh_16.440 | 16-440 | TM0900 | NA | 2013 | Karratha Chichester | Chichester | -20.7272 | 116.98541 | S. A. Thompson, via Judy Dunlop, DBCA |
| Dh_16.480 | 16-480 | TM0866 | NA | 2013 | Gudai-Darri East | Hamersley | -22.5869 | 119.0911 | C. Cole and N. Watson, via Judy Dunlop, DBCA |
| Dh_16.479 | 16-479 | TM0865 | NA | 2013 | Gudai-Darri West | Hamersley | -22.5051 | 118.9388 | M. Greenham and S. Schmidt, via Judy Dunlop, DBCA |
| Dh_16.207 | 16-207 | NA | M | 2013 | Nimingarra | McLarty | -20.39181 | 120.03847 | Judy Dunlop, DBCA |
| Dh_16.435 | 16-435 | TM0904 | NA | 2013 | Quarry Site 2a | Chichester | -20.96974 | 118.75231 | S. A. Thompson, via Judy Dunlop, DBCA |
| Dh_16.443 | 16-443 | TM0903 | NA | 2013 | Quarry Site 2a | Chichester | -20.9816 | 118.73626 | S. A. Thompson, via Judy Dunlop, DBCA |
| Dh_16.447 | 16-447 | TM0907 | NA | 2013 | Quarry Site 2a | Chichester | -20.9692 | 118.75178 | S. A. Thompson, via Judy Dunlop, DBCA |
| Dh_16.445 | 16-445 | TM0914 | NA | 2013 | Quarry Site 2b | Roebourne | -20.03331 | 119.19054 | S. A. Thompson, via Judy Dunlop, DBCA |
| Dh_16.496 | 16-496 | TM0884 | NA | 2013 | Tom Price East | Chichester | -22.04139 | 118.74722 | B. Greatwich and L. Smith, via Judy Dunlop, DBCA |
| Dh_16.497 | 16-497 | TM0885 | NA | 2013 | Tom Price East | Chichester | -22.04083 | 118.74889 | B. Greatwich and L. Smith, via Judy Dunlop, DBCA |
| Dh_16.498 | 16-498 | TM0886 | NA | 2013 | Tom Price East | Chichester | -22.04056 | 118.74917 | B. Greatwich and L. Smith, via Judy Dunlop, DBCA |
| Dh_16.493 | 16-493 | TM0881 | NA | 2013 | Tom Price West | Hamersley | -22.09889 | 117.8775 | A. Heidrich and C. Knuckey, via Judy Dunlop, DBCA |
| Dh_16.467 | 16-467 | TM0931 | NA | 2014 | Abydos Turner Yandee | Chichester | -21.14889 | 119.10806 | B. Wingfield, via Judy Dunlop, DBCA |
| Dh_16.270 | 16-270 | NA | F | 2014 | Cane River CP | Hamersley | -21.98904304 | 115.56981 | Judy Dunlop, DBCA |
| Dh_16.241 | 16-241 | NA | F | 2014 | Coolawanyah Station North | Chichester | -21.481725 | 117.914938 | Ryan Ellis, Phoenix Environmental, via Judy Dunlop, DBCA |
| Dh_16.239 | 16-239 | NA | M | 2014 | Coolawanyah Station North | Chichester | -21.478777 | 117.914488 | Ryan Ellis, Phoenix Environmental, via Judy Dunlop, DBCA |
| Dh_16.242 | 16-242 | NA | M | 2014 | Coolawanyah Station North | Chichester | -21.482287 | 117.915391 | Ryan Ellis, Phoenix Environmental, via Judy Dunlop, DBCA |
| Dh_16.249 | 16-249 | NA | F | 2014 | Coolawanyah Station South | Chichester | -21.691895 | 117.860076 | NA |
| Dh_16.251 | 16-251 | NA | F | 2014 | Coolawanyah Station South | Chichester | -21.69021 | 117.863437 | Ryan Ellis, Phoenix Environmental, via Judy Dunlop, DBCA |
| Dh_16.254 | 16-254 | NA | F | 2014 | Coolawanyah Station South | Chichester | -21.689725 | 117.865248 | Ryan Ellis, Phoenix Environmental, via Judy Dunlop, DBCA |
| Dh_16.253 | 16-253 | NA | M | 2014 | Coolawanyah Station South | Chichester | -21.688592 | 117.868643 | Ryan Ellis, Phoenix Environmental, via Judy Dunlop, DBCA |
| Dh_16.255 | 16-255 | NA | M | 2014 | Coolawanyah Station South | Chichester | -21.690379 | 117.868146 | NA |
| Dh_16.256 | 16-256 | NA | M | 2014 | Coolawanyah Station South | Chichester | -21.694179 | 117.85919 | Ryan Ellis, Phoenix Environmental, via Judy Dunlop, DBCA |
| Dh_16.320 | 16-320 | NA | F | 2014 | De grey Station | Roebourne | -20.29253502 | 119.112099 | Judy Dunlop, DBCA |
| Dh_16.319 | 16-319 | NA | M | 2014 | De grey Station | Roebourne | -20.29013604 | 119.105868 | Judy Dunlop, DBCA |
| Dh_16.321 | 16-321 | NA | M | 2014 | De grey Station | Roebourne | -20.29067097 | 119.10634 | Judy Dunlop, DBCA |
| Dh_16.311 | 16-311 | NA | M | 2014 | Dolphin Island | Roebourne | -20.48865203 | 116.831265 | Judy Dunlop, DBCA |
| Dh_16.338 | 16-338 | NA | F | 2014 | Indee Quarry 2 | Chichester | -20.87521099 | 118.58141 | Judy Dunlop, DBCA |
| Dh_16.343 | 16-343 | NA | F | 2014 | Indee Quarry 2 | Chichester | -20.8759061 | 118.5873215 | Judy Dunlop, DBCA |
| Dh_16.347 | 16-347 | NA | F | 2014 | Indee Quarry 2 | Chichester | -20.88096902 | 118.586539 | NA |
| Dh_16.333 | 16-333 | NA | M | 2014 | Indee Quarry 2 | Chichester | -20.88096902 | 118.585841 | Judy Dunlop, DBCA |
| Dh_16.337 | 16-337 | NA | M | 2014 | Indee Quarry 2 | Chichester | -20.87569202 | 118.584378 | Judy Dunlop, DBCA |
| Dh_16.342 | 16-342 | NA | M | 2014 | Indee Quarry 2 | Chichester | -20.87649602 | 118.587414 | Judy Dunlop, DBCA |
| Dh_16.274 | 16-274 | NA | F | 2014 | Mallina Station | Chichester | -21.18300201 | 117.953743 | NA |
| Dh_16.281 | 16-281 | NA | F | 2014 | McPhee Creek Mine | Chichester | -21.615736 | 120.076817 | C. Knuckey, via Judy Dunlop, DBCA |
| Dh_16.285 | 16-285 | NA | F | 2014 | Millstream Python | Chichester | -21.33392103 | 117.240584 | Judy Dunlop, DBCA |
| Dh_16.286 | 16-286 | NA | M | 2014 | Millstream Python | Chichester | -21.33058185 | 117.2393083 | Judy Dunlop, DBCA |
| Dh_16.287 | 16-287 | NA | M | 2014 | Millstream Python | Chichester | -21.33392103 | 117.240584 | Judy Dunlop, DBCA |
| Dh_16.279 | 16-279 | NA | F | 2014 | Mt Dove Mine | Chichester | -20.935597 | 118.463889 | NA |
| Dh_16.229 | 16-229 | NA | M | 2014 | Pannawonica Red Hill | Hamersley | -22.013848 | 116.533176 | Ryan Ellis, Phoenix Environmental, via Judy Dunlop, DBCA |
| Dh_16.236 | 16-236 | NA | F | 2014 | Pannawonica Yarraloola | Hamersley | -21.797203 | 116.159628 | Ryan Ellis, Phoenix Environmental, via Judy Dunlop, DBCA |
| Dh_16.230 | 16-230 | NA | M | 2014 | Pannawonica Yarraloola | Hamersley | -21.79902 | 116.159946 | Ryan Ellis, Phoenix Environmental, via Judy Dunlop, DBCA |
| Dh_16.233 | 16-233 | NA | M | 2014 | Pannawonica Yarraloola | Hamersley | -21.797111 | 116.159415 | Ryan Ellis, Phoenix Environmental, via Judy Dunlop, DBCA |
| Dh_16.231 | 16-231 | NA | NA | 2014 | Pannawonica Yarraloola | Hamersley | -21.786242 | 116.134639 | Ryan Ellis, Phoenix Environmental, via Judy Dunlop, DBCA |
| Dh_16.103 | 16-103 | NA | F | 2014 | Quoll knoll | Chichester | -22.09662 | 119.23706 | NA |
| Dh_16.100 | 16-100 | NA | M | 2014 | Quoll knoll | Chichester | -22.09745 | 119.23656 | Brent Johnson and Hannah Anderson, DBCA |
| Dh_16.267 | 16-267 | NA | F | 2014 | Red Hill Hammersley Mid | Hamersley | -22.18236602 | 116.27203 | Judy Dunlop, DBCA |
| Dh_16.268 | 16-268 | NA | F | 2014 | Red Hill Hammersley Mid | Hamersley | -22.18081403 | 116.273275 | Judy Dunlop, DBCA |
| Dh_16.465 | 16-465 | TM0938 | NA | 2014 | Wodgina | Chichester | -21.16889 | 118.64972 | C. Knuckey, via Judy Dunlop, DBCA |
| Dh_16.468 | 16-468 | TM0932 | NA | 2014 | Wodgina | Chichester | -21.19778 | 118.655 | C. Knuckey, via Judy Dunlop, DBCA |
| Dh_16.473 | 16-473 | TM0937 | NA | 2014 | Wodgina | Chichester | -21.17111 | 118.65083 | C. Knuckey, via Judy Dunlop, DBCA |
| Dh_16.324 | 16-324 | NA | F | 2014 | Yarrie Station | Chichester | -20.88387301 | 120.118 | NA |
| Dh_16.322 | 16-322 | NA | M | 2014 | Yarrie Station | Chichester | -20.88284204 | 120.118579 | Judy Dunlop, DBCA |
| Dh_16.323 | 16-323 | NA | M | 2014 | Yarrie Station | Chichester | -20.88316499 | 120.118278 | Judy Dunlop, DBCA |
| Dh_16.325 | 16-325 | NA | M | 2014 | Yarrie Station | Chichester | -20.88384703 | 120.118497 | Judy Dunlop, DBCA |
| Dh_16.326 | 16-326 | NA | M | 2014 | Yarrie Station | Chichester | -20.88535602 | 120.120658 | Judy Dunlop, DBCA |
| Dh_16.327 | 16-327 | NA | M | 2014 | Yarrie Station | Chichester | -20.88452102 | 120.118883 | Judy Dunlop, DBCA |
| Dh_16.859 | 16-859 | NA | F | 2015 | Abydos Turner Yandee | Chichester | -21.2504563 | 119.0518658 | Astrid and Damien, Ecoscape |
| Dh_16.861 | 16-861 | NA | F | 2015 | Abydos Turner Yandee | Chichester | -21.25799218 | 119.0552458 | Astrid and Damien, Ecoscape |
| Dh_16.863 | 16-863 | NA | F | 2015 | Abydos Turner Yandee | Chichester | -21.24463569 | 119.0824378 | Astrid and Damien, Ecoscape |
| Dh_16.860 | 16-860 | NA | M | 2015 | Abydos Turner Yandee | Chichester | -21.25822331 | 119.0555477 | Astrid and Damien, Ecoscape |
| Dh_16.866 | 16-866 | NA | M | 2015 | Abydos Turner Yandee | Chichester | -21.18800902 | 119.1037714 | Astrid and Damien, Ecoscape |
| Dh_16.093 | 16-093 | NA | M | 2015 | Dolphin Island | Roebourne | -20.48935804 | 116.830616 | NA |
| Dh_16.183 | 16-183 | NA | M | 2015 | Karratha Roeburn | Roebourne | -20.734817 | 116.846368 | Judy Dunlop, DBCA |
| Dh_16.075 | 16-075 | NA | M | 2015 | Meentheena CP | Chichester | -21.35132899 | 120.475953 | Judy Dunlop, DBCA |
| Dh_16.088 | 16-088 | NA | F | 2015 | Mt Florance Station | Chichester | -21.77477896 | 117.911578 | Judy Dunlop, DBCA |
| Dh_16.092 | 16-092 | NA | F | 2015 | Mt Florance Station | Chichester | -21.77271802 | 117.920635 | Judy Dunlop, DBCA |
| Dh_17.056 | 17-056 | NA | F | 2015 | Pannawonica | Hamersley | -21.67344322 | 116.4036452 | John Trainor, Astron Environmental |
| Dh_17.053 | 17-053 | NA | M | 2015 | Pannawonica | Chichester | -21.70945392 | 116.4783567 | John Trainor, Astron Environmental |
| Dh_17.055 | 17-055 | NA | M | 2015 | Pannawonica | Chichester | -21.68847944 | 116.4912503 | John Trainor, Astron Environmental |
| Dh_16.105 | 16-105 | NA | F | 2015 | Pannawonica Yarraloola | Hamersley | -21.84405 | 116.086406 | Mark Cowan & Hannah Anderson, DBCA |
| Dh_16.112 | 16-112 | NA | F | 2015 | Pannawonica Yarraloola | Hamersley | -21.75297981 | 116.0165271 | Mark Cowan & Hannah Anderson, DBCA |
| Dh_16.120 | 16-120 | NA | F | 2015 | Pannawonica Yarraloola | Hamersley | -21.80924996 | 116.1648298 | Mark Cowan & Hannah Anderson, DBCA |
| Dh_16.123 | 16-123 | NA | F | 2015 | Pannawonica Yarraloola | Hamersley | -21.79496099 | 116.0986509 | Mark Cowan & Hannah Anderson, DBCA |
| Dh_16.125 | 16-125 | NA | F | 2015 | Pannawonica Yarraloola | Hamersley | -21.79461197 | 116.0925219 | Mark Cowan & Hannah Anderson, DBCA |
| Dh_16.128 | 16-128 | NA | F | 2015 | Pannawonica Yarraloola | Hamersley | -21.76917675 | 116.0154788 | Mark Cowan & Hannah Anderson, DBCA |
| Dh_16.134 | 16-134 | NA | F | 2015 | Pannawonica Yarraloola | Hamersley | -21.7287199 | 115.9585818 | Mark Cowan & Hannah Anderson, DBCA |
| Dh_16.135 | 16-135 | NA | F | 2015 | Pannawonica Yarraloola | Hamersley | -21.74159895 | 116.1405129 | Mark Cowan & Hannah Anderson, DBCA |
| Dh_16.139 | 16-139 | NA | F | 2015 | Pannawonica Yarraloola | Hamersley | -21.809825 | 116.168154 | Mark Cowan & Hannah Anderson, DBCA |
| Dh_16.107 | 16-107 | NA | M | 2015 | Pannawonica Yarraloola | Hamersley | -21.78422 | 116.156425 | Mark Cowan & Hannah Anderson, DBCA |
| Dh_16.111 | 16-111 | NA | M | 2015 | Pannawonica Yarraloola | Hamersley | -21.78438999 | 116.1496599 | Mark Cowan & Hannah Anderson, DBCA |
| Dh_16.114 | 16-114 | NA | M | 2015 | Pannawonica Yarraloola | Hamersley | -21.77173994 | 116.1592249 | Mark Cowan & Hannah Anderson, DBCA |
| Dh_16.117 | 16-117 | NA | M | 2015 | Pannawonica Yarraloola | Hamersley | -21.72860742 | 115.957545 | Mark Cowan & Hannah Anderson, DBCA |
| Dh_16.118 | 16-118 | NA | M | 2015 | Pannawonica Yarraloola | Hamersley | -21.80915399 | 116.165388 | Mark Cowan & Hannah Anderson, DBCA |
| Dh_16.121 | 16-121 | NA | M | 2015 | Pannawonica Yarraloola | Hamersley | -21.81292199 | 116.1662879 | Mark Cowan & Hannah Anderson, DBCA |
| Dh_16.129 | 16-129 | NA | M | 2015 | Pannawonica Yarraloola | Hamersley | -21.78337997 | 116.156684 | Mark Cowan & Hannah Anderson, DBCA |
| Dh_16.132 | 16-132 | NA | M | 2015 | Pannawonica Yarraloola | Hamersley | -21.82006597 | 116.108757 | Mark Cowan & Hannah Anderson, DBCA |
| Dh_16.137 | 16-137 | NA | M | 2015 | Pannawonica Yarraloola | Hamersley | -21.842698 | 116.084481 | Mark Cowan & Hannah Anderson, DBCA |
| Dh_16.138 | 16-138 | NA | M | 2015 | Pannawonica Yarraloola | Hamersley | -21.77895558 | 116.1451459 | Mark Cowan & Hannah Anderson, DBCA |
| Dh_16.140 | 16-140 | NA | M | 2015 | Pannawonica Yarraloola | Hamersley | -21.77439792 | 116.1643412 | Mark Cowan & Hannah Anderson, DBCA |
| Dh_16.084 | 16-084 | NA | F | 2015 | Poondano | Chichester | -20.448503 | 118.7738377 | Judy Dunlop, DBCA |
| Dh_16.080 | 16-080 | NA | M | 2015 | Poondano | Chichester | -20.45580503 | 118.816961 | Judy Dunlop, DBCA |
| Dh_16.087 | 16-087 | NA | M | 2015 | Poondano | Chichester | -20.497562 | 118.8240509 | Judy Dunlop, DBCA |
| Dh_16.098 | 16-098 | NA | M | 2015 | Quoll knoll | Chichester | -22.0966 | 119.2371 | Judy Dunlop, DBCA |
| Dh_16.149 | 16-149 | NA | F | 2015 | Red Hill Chichester | Chichester | -21.19321 | 116.23553 | John Angus & Sean Garretson, DBCA |
| Dh_16.158 | 16-158 | NA | F | 2015 | Red Hill Chichester | Chichester | -21.19684 | 116.25483 | John Angus & Sean Garretson, DBCA |
| Dh_16.159 | 16-159 | NA | F | 2015 | Red Hill Chichester | Roebourne | -21.08626 | 116.25597 | John Angus & Sean Garretson, DBCA |
| Dh_16.143 | 16-143 | NA | M | 2015 | Red Hill Chichester | Roebourne | -21.14653 | 116.21612 | John Angus & Sean Garretson, DBCA |
| Dh_16.145 | 16-145 | NA | M | 2015 | Red Hill Chichester | Roebourne | -21.14635 | 116.21632 | John Angus & Sean Garretson, DBCA |
| Dh_16.146 | 16-146 | NA | M | 2015 | Red Hill Chichester | Roebourne | -21.08828 | 116.25264 | John Angus & Sean Garretson, DBCA |
| Dh_16.148 | 16-148 | NA | M | 2015 | Red Hill Chichester | Roebourne | -21.15287 | 116.25345 | John Angus & Sean Garretson, DBCA |
| Dh_16.150 | 16-150 | NA | M | 2015 | Red Hill Chichester | Roebourne | -21.14635 | 116.21632 | John Angus & Sean Garretson, DBCA |
| Dh_16.152 | 16-152 | NA | M | 2015 | Red Hill Chichester | Roebourne | -21.14582 | 116.21691 | John Angus & Sean Garretson, DBCA |
| Dh_16.155 | 16-155 | NA | M | 2015 | Red Hill Chichester | Roebourne | -21.14934 | 116.21376 | John Angus & Sean Garretson, DBCA |
| Dh_16.156 | 16-156 | NA | M | 2015 | Red Hill Chichester | Roebourne | -21.14934 | 116.21376 | John Angus & Sean Garretson, DBCA |
| Dh_16.882 | 16-882 | NA | F | 2016 | Karijini NP | Hamersley | -22.47574101 | 118.562164 | Judy Dunlop, DBCA |
| Dh_16.883 | 16-883 | NA | F | 2016 | Karijini NP | Hamersley | -22.48075599 | 118.564248 | Judy Dunlop, DBCA |
| Dh_16.881 | 16-881 | NA | M | 2016 | Karijini NP | Hamersley | -22.47732804 | 118.562863 | Judy Dunlop, DBCA |
| Dh_16.910 | 16-910 | NA | M | 2016 | Karlamilyi East | Rudall | -22.46521 | 122.26054 | Judy Dunlop, DBCA |
| Dh_17.052 | 17-052 | NA | F | 2016 | Pannawonica Yarraloola | Hamersley | -21.73446816 | 116.1772623 | John Trainor, Astron Environmental |
| Dh_17.057 | 17-057 | NA | F | 2016 | Pannawonica Yarraloola | Hamersley | -21.72046906 | 116.1744125 | John Trainor, Astron Environmental |
| Dh_16.905 | 16-905 | NA | F | 2016 | Red Hill Hammersley Mid | Hamersley | -22.18097697 | 116.273209 | Judy Dunlop, DBCA |
| Dh_16.906 | 16-906 | NA | M | 2016 | Red Hill Hammersley Mid | Hamersley | -22.18259803 | 116.275602 | Judy Dunlop, DBCA |
| Dh_16.908 | 16-908 | NA | M | 2016 | Red Hill Hammersley Mid | Hamersley | -22.18082098 | 116.268086 | Judy Dunlop, DBCA |
| Dh_16.898 | 16-898 | NA | F | 2016 | Yarrie Station | Chichester | -20.88540397 | 120.11976 | NA |
| Dh_17.078 | 17-078 | NA | F | 2017 | Abydos Turner Yandee | Chichester | -21.18864538 | 119.10515 | Astrid Heidrich, EcoScape |
| Dh_17.088_MT | 17-088_MT | NA | F | 2017 | Abydos Turner Yandee | Chichester | -21.27231872 | 119.0538477 | Astrid Heidrich, EcoScape |
| Dh_17.079 | 17-079 | NA | M | 2017 | Abydos Turner Yandee | Chichester | -21.28712192 | 119.0574259 | NA |
| Dh_17.093 | 17-093 | NA | M | 2017 | Abydos Turner Yandee | Chichester | -21.25498924 | 119.0537614 | Astrid Heidrich, EcoScape |

**Appendix S6. Investigating IBB using different genetic clustering approaches**

We used the *tess3r* algorithm to perform genetic clustering, as it is spatially explicit, assumes a continuous distribution of ancestry proportions over space and performs well with low genetic divergence (Caye et al. 2016). We thus felt *tess3r* best represented northern quoll population structure given their large dispersal capacity and promiscuous mating system (Chan et al. 2020), though two alternative methods were run for comparison (below). We performed 100 independent runs for K= 1-7 (based on PCoA groupings) using default parameters, and withheld 10% of the data for cross validation. The optimal value for K was determined by the cross-entropy criterion, and the run with the lowest root mean squared error was presented.

We compared our *tess3R* (Caye & Francois 2016; Caye et al. 2016) result across an additional two genetic clustering methods implemented through R (R Core Team 2020). We used the ‘snmf’ function in *LEA* (Frichot & François 2015) and the ‘dapc’ function in *adegenet* (Jombart 2008; Jombard et al. 2010; Jombart & Ahmed 2011).We chose these methods, as all are model-free and do not make assumptions about HWE. We confirmed the detection of de novo structure across methods, as each uses a slightly different approach to detecting genetic clusters. sNMF uses sparse non-negative matrix factorization algorithms to estimate individual admixture coefficients, with estimates comparable to ADMIXTURE, STRUCTURE and fastSTRUCTURE (Frichot & François 2015). *tess3r* also uses the sNMF algorithm, but is spatially explicit, assuming continuously distributed ancestry proportions over geographic space (and is equivalent to TESS3; Caye et al. 2016). Finally, Discriminant Analysis of Principal Components (DAPC) is a multivariate method that identifies clusters of genetically similar individuals by transforming the data using PCA and then using sequential K-means to identify groups of individuals. The most informative PC axes are retained for Discriminant Analysis, to determine between-group variability. DAPC generally performs better than STRUCTURE at characterising population subdivision (Jombard et al. 2010).

We tested K = 1 to 7 across all methods, and set the maximum number of iterations to 1000, ran 100 repetitions, and kept the default values for the remaining parameters for both *tess3r* and *LEA* (sNMF). For DAPC, we retained all PC axes during K means clustering (using the function ‘find.clusters’, with 1000 iterations). We then performed cross-validation to determine the optimal number of PC axes to retain for discriminant analysis (60), and retained one discriminant function. All three methods identified K = 2 as the best value for K, with varying degrees of admixture (DAPC did not detect any admixture). Results are presented below.


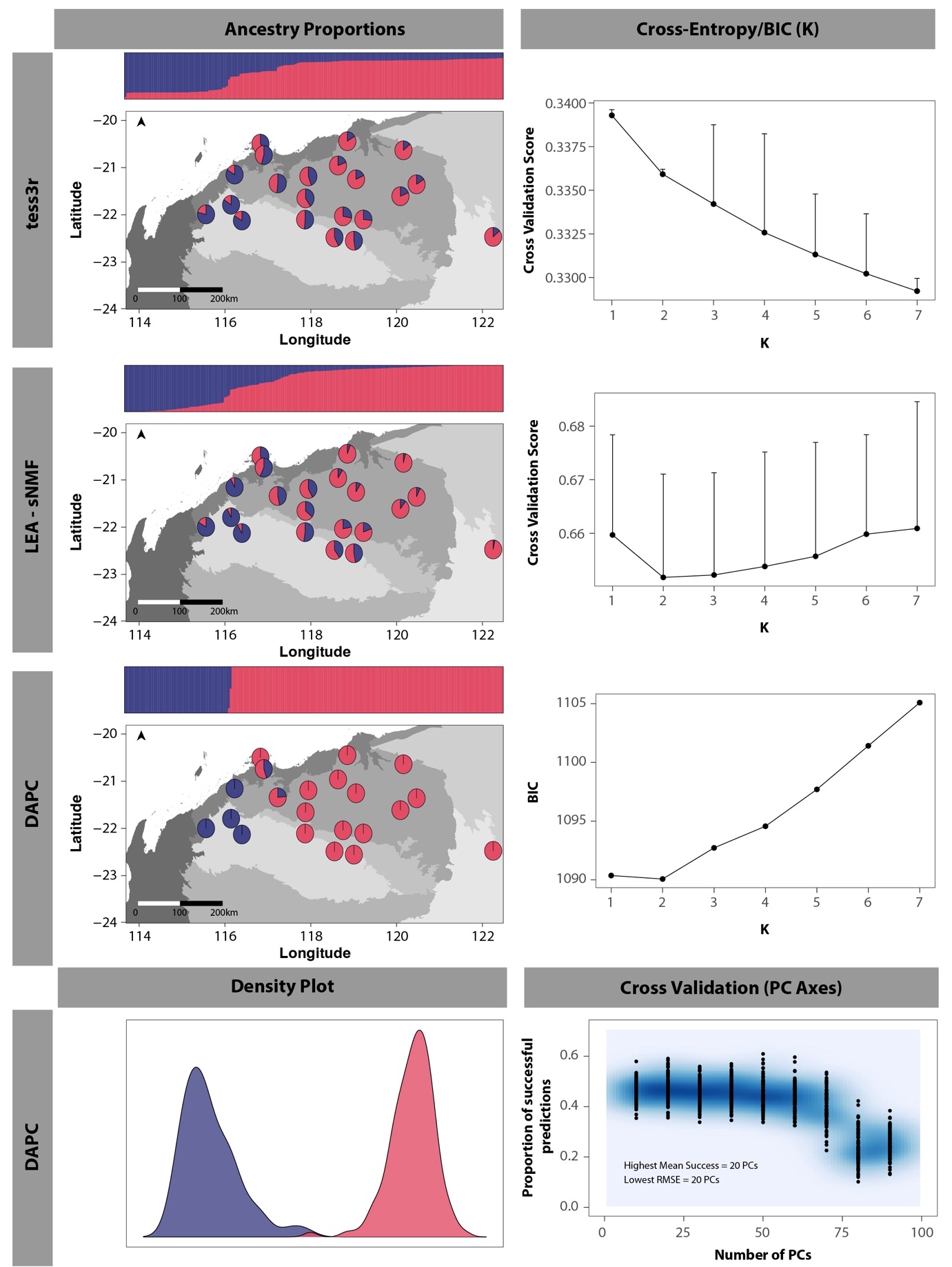


**Appendix S7. ResistanceGA resistance surface optimisation**

We used the R package *ResistanceGA* (Peterman 2018) following methods outlined in Peterman et al. (2014), to optimise resistance surfaces using a genetic algorithm to search parameter space for optimal resistance values. Briefly, categorical and continuous rasters undergo statistical transformation (described in Peterman et al. 2014; Ruiz-Lopez et al. 2016). After transformation, pairwise effective distances are calculated among individuals using ‘commute distance’ (equivalent to ﻿CIRCUITSCAPE resistance distance; Klein & Randić 1993; McRae et al. 2008) and an MLPE model is fit. This process is carried out multiple times, optimising the objective function (using the default; log-likelihood) until a final optimised surface is achieved.

To avoid including correlated variables in the IBR analysis, we performed initial, single-surface optimisations on each raster and chose the top-ranked variable (using the sample size corrected Akaike’s Information Criterion (AICc); Akaike 1974) within each set (Spearman’s |r_s_|>0.7). This process identified that terrain ruggedness was best represented by VRM, and substrate was best represented by silt (compared to other correlated variables). The variables used in the final analysis were distance to water, VRM, fire frequency, forest cover, spinifex, silt and WII.

**Appendix S8. MaxEnt SDM with occurrence records.**

The following map shows the predicted probability of northern quoll occurrence. White triangles represent occurrence record locations.


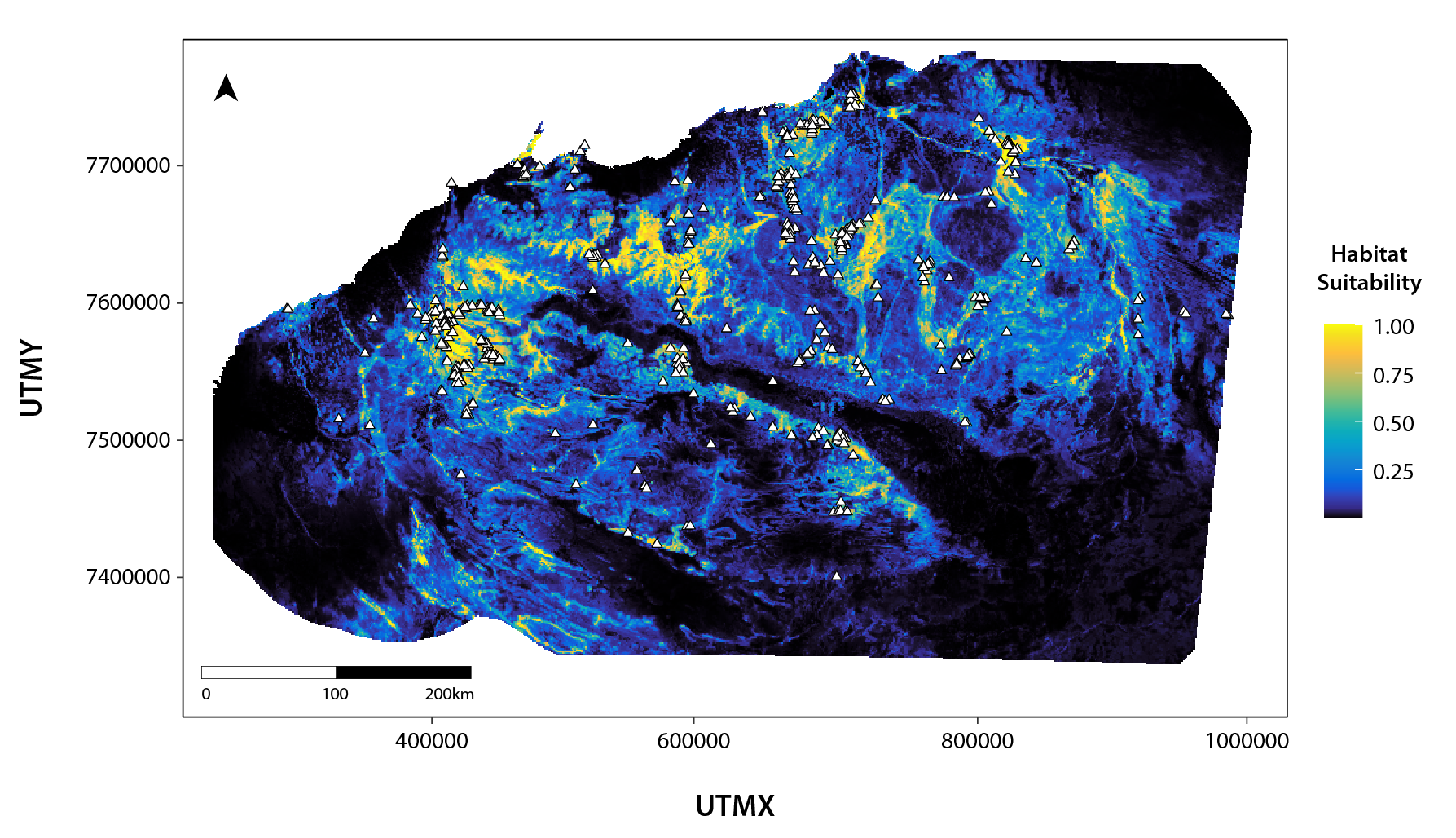


**Appendix S9. MaxEnt ROC plot and variable contributions**

a) ROC plot for final, optimised MaxEnt model, displaying the AUC for the training and testing datasets; b) Variable contributions to the final MaxEnt model based on permutation importance.

**a)**

**
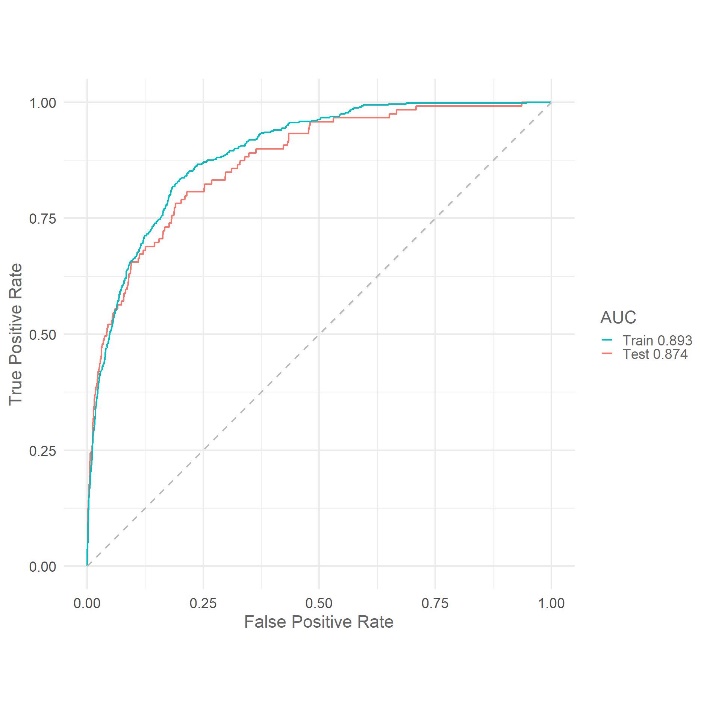
**

**b)**

**
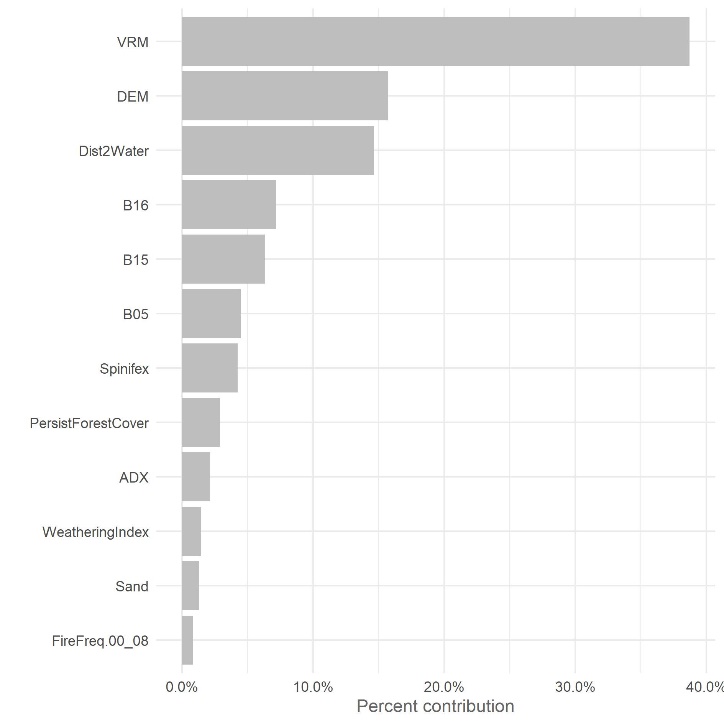
**

**Appendix S10. MaxEnt species response curves**

Northern quoll marginal response curves are shown for each of the 12 variables included in the final SDM. The red line represents the mean across cross validation folds with associated error. The dashed blue line represents the final model trained with 80% of the data (i.e. after cross validation folds were merged, and the final model was re-trained using 80% of the data).

**
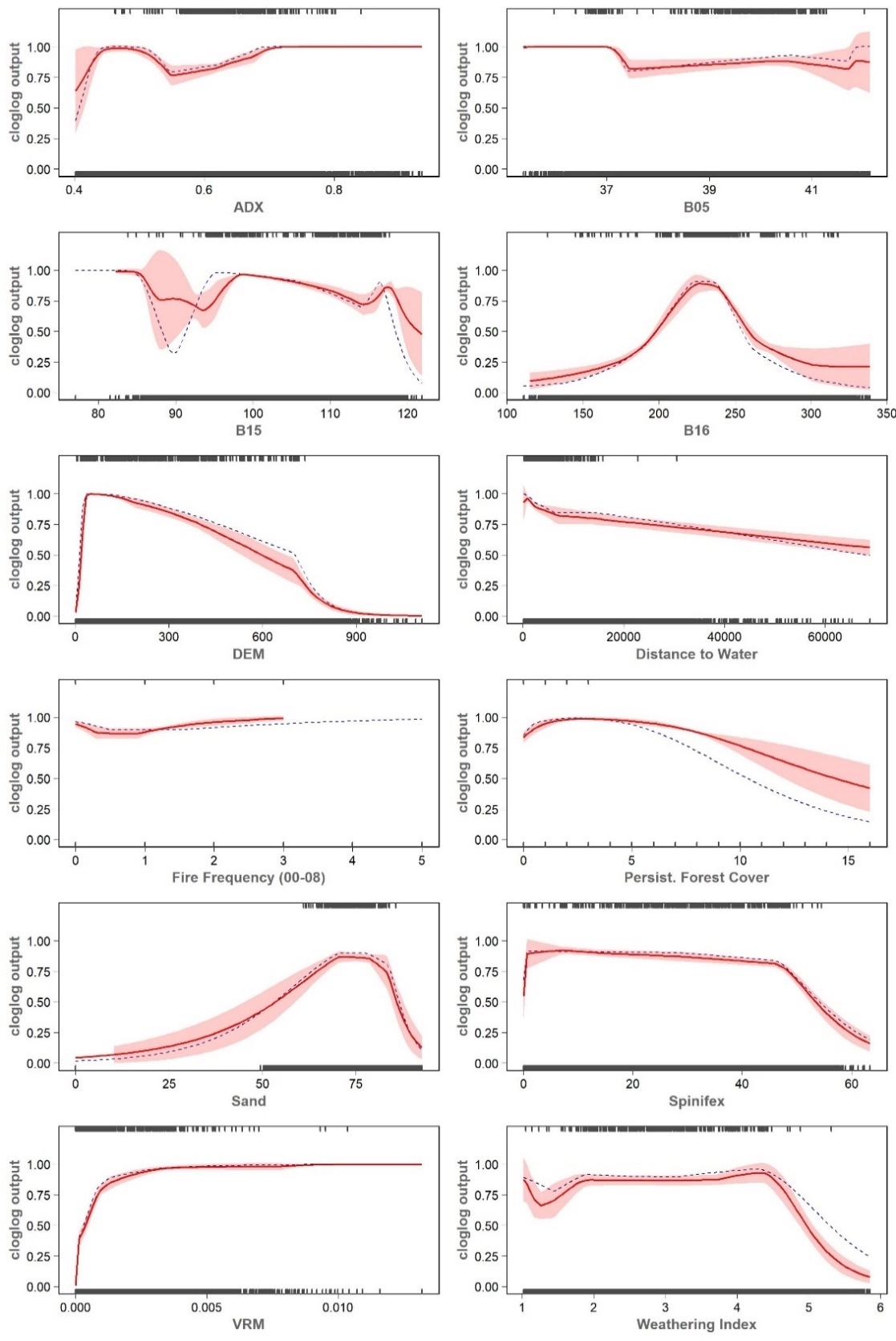
**

**Appendix S11. Population genetic summary statistics for the two genetic clusters detected**

Mean population genetic summary statistics (±SE), including N_Ind_ (number of individuals), N_Loci_ (number of loci), %P (percentage of polymorphic loci), Na (number of alleles), Ho (observed heterozygosity), He (expected heterozygosity), F (fixation index), PHt (proportion of heterozygous loci), F_IS_ and F_ST_, for K = 2 ancestral populations in the Pilbara.

|  | **Cluster 1** | **Cluster 2** | **Total** |
| --- | --- | --- | --- |
| **N_Ind_** | 53 | 115 | 168 |
| **N_loci_** | 3635 | 3635 | 3635 |
| **%P** | 94.64% | 98.65% | 96.64% ± 2.01% |
| **Na** | 1.946 ± 0.004 | 1.987 ± 0.002 | 1.966 ± 0.002 |
| **Ho** | 0.242 ± 0.003 | 0.244 ± 0.003 | 0.243 ± 0.002 |
| **He** | 0.254 ± 0.003 | 0.257 ± 0.003 | 0.256 ± 0.002 |
| **F** | 0.042 ± 0.003 | 0.045 ± 0.002 | 0.043 ± 0.002 |
| **F_IS_** | - | - | 0.044 ± 0.002 |
| **F_ST_** | - | - | 0.02 ± 4.309E-04 |

**Appendix S12. Mantel**

Mantel plots showing the correlation between geographic (km) and genetic distance (Cluster 1: *rxy* = 0.125, P = 0.068; Cluster 2: *rxy* = 0.261, P = 0.002).


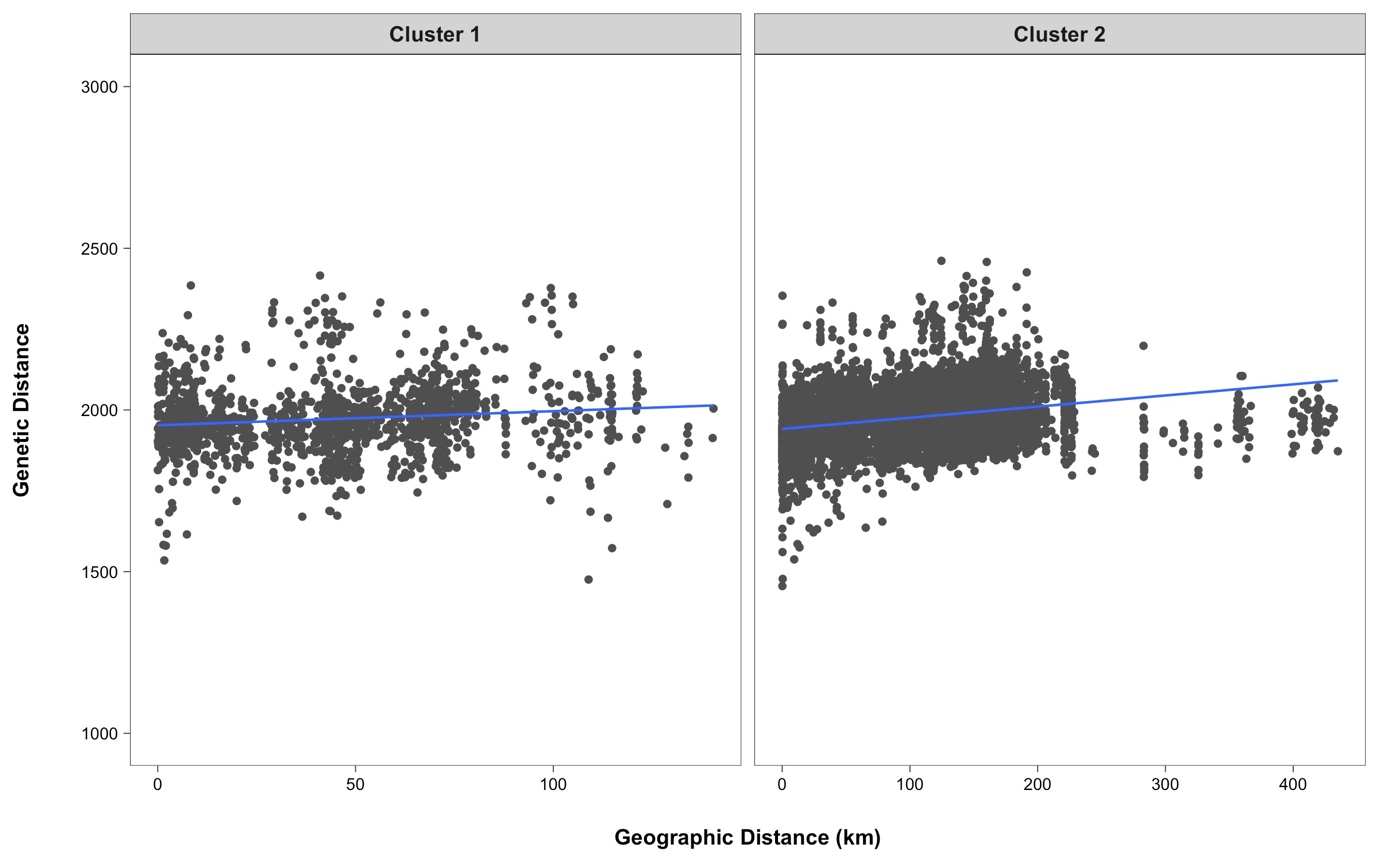


**Appendix S13. *ResistanceGA* model selection**

Model selection results for single- and multi-surface optimised layers, including the Objective function (LL; log-likelihood), number of parameters (k), Akaike Information Criteria (AIC), sample size corrected AIC (AICc), marginal and conditional R^2^ (R^2^m, R^2^c), log-likelihood (LL), the difference in AICc compared to the top-ranked model (∆AICc) and the AICc weight.

| **Surface** | **Obj.func LL** | **k** | **AIC** | **AICc** | **R^2^m** | **R^2^c** | **LL** | **∆AICc** | **Weight** |
| --- | --- | --- | --- | --- | --- | --- | --- | --- | --- |
| Distance to Water * Silt | -1907.97 | 7 | 3829.94 | 3831.47 | 0.54 | 0.83 | -1907.97 | 0 | 0.97 |
| Distance to Water * Silt * Weathering | -1908.35 | 10 | 3836.71 | 3839.85 | 0.54 | 0.83 | -1908.35 | 8.38 | 0.01 |
| Distance to Water * Silt * Spinifex | -1908.68 | 10 | 3837.36 | 3840.51 | 0.54 | 0.83 | -1908.68 | 9.04 | 0.01 |
| Distance to Water * Silt * VRM | -1911.88 | 10 | 3843.76 | 3846.9 | 0.54 | 0.83 | -1911.88 | 15.43 | 0 |
| Distance to Water * Persistent Forest Cover * Silt | -1912.23 | 10 | 3844.45 | 3847.59 | 0.54 | 0.83 | -1912.23 | 16.12 | 0 |
| Distance to Water * Fire Frequency * Silt | -1914.09 | 10 | 3848.18 | 3851.33 | 0.54 | 0.83 | -1914.09 | 19.86 | 0 |
| Distance to Water * Persistent Forest Cover * Silt * VRM | -1912 | 13 | 3849.99 | 3855.43 | 0.55 | 0.83 | -1912 | 23.95 | 0 |
| Distance to Water * Persistent Forest Cover * Silt * Spinifex | -1913.47 | 13 | 3852.93 | 3858.37 | 0.54 | 0.83 | -1913.47 | 26.9 | 0 |
| Distance to Water * Fire Frequency * Silt * Weathering | -1914.14 | 13 | 3854.28 | 3859.71 | 0.54 | 0.83 | -1914.14 | 28.24 | 0 |
| Distance to Water * Fire Frequency * Persistent Forest Cover * Silt | -1917.75 | 13 | 3861.49 | 3866.92 | 0.54 | 0.83 | -1917.75 | 35.45 | 0 |
| Distance to Water * Silt * Spinifex * Weathering | -1917.8 | 13 | 3861.6 | 3867.03 | 0.54 | 0.83 | -1917.8 | 35.56 | 0 |
| Distance to Water * Silt * Spinifex * VRM | -1922.41 | 13 | 3870.83 | 3876.26 | 0.55 | 0.82 | -1922.41 | 44.79 | 0 |
| Distance to Water * Persistent Forest Cover * Silt * Weathering | -1923.12 | 13 | 3872.23 | 3877.66 | 0.54 | 0.82 | -1923.12 | 46.19 | 0 |
| Distance to Water * Fire Frequency * Silt * Spinifex | -1924.52 | 13 | 3875.05 | 3880.48 | 0.54 | 0.82 | -1924.52 | 49.01 | 0 |
| Distance to Water * Fire Frequency * VRM | -1929.89 | 10 | 3879.79 | 3882.93 | 0.57 | 0.85 | -1929.89 | 51.46 | 0 |
| Distance to Water * Spinifex * VRM | -1930.79 | 10 | 3881.57 | 3884.71 | 0.56 | 0.84 | -1930.79 | 53.24 | 0 |
| Distance to Water * Spinifex * VRM * Weathering | -1927.27 | 13 | 3880.53 | 3885.97 | 0.57 | 0.85 | -1927.27 | 54.49 | 0 |
| Distance to Water * Fire Frequency * VRM * Weathering | -1929.31 | 13 | 3884.61 | 3890.04 | 0.57 | 0.84 | -1929.31 | 58.57 | 0 |
| Distance to Water * Fire Frequency * Silt * VRM | -1930.55 | 13 | 3887.1 | 3892.53 | 0.58 | 0.85 | -1930.55 | 61.06 | 0 |
| Distance to Water * Persistent Forest Cover * VRM | -1935.71 | 10 | 3891.42 | 3894.56 | 0.54 | 0.82 | -1935.71 | 63.09 | 0 |
| Distance to Water * Fire Frequency * Spinifex * VRM | -1935.27 | 13 | 3896.54 | 3901.97 | 0.58 | 0.86 | -1935.27 | 70.5 | 0 |
| Distance to Water * VRM | -1943.93 | 7 | 3901.86 | 3903.39 | 0.55 | 0.84 | -1943.93 | 71.92 | 0 |
| Distance to Water * VRM * Weathering | -1942.43 | 10 | 3904.86 | 3908.01 | 0.54 | 0.83 | -1942.43 | 76.54 | 0 |
| Fire Frequency * Spinifex * VRM | -1943.32 | 10 | 3906.65 | 3909.79 | 0.58 | 0.86 | -1943.32 | 78.32 | 0 |
| Distance to Water * Persistent Forest Cover * VRM * Weathering | -1939.97 | 13 | 3905.94 | 3911.37 | 0.55 | 0.83 | -1939.97 | 79.9 | 0 |
| Distance to Water * Fire Frequency * Persistent Forest Cover * VRM | -1940.83 | 13 | 3907.65 | 3913.09 | 0.55 | 0.83 | -1940.83 | 81.61 | 0 |
| Distance to Water * Persistent Forest Cover * Spinifex * VRM | -1941.73 | 13 | 3909.47 | 3914.9 | 0.55 | 0.82 | -1941.73 | 83.43 | 0 |
| Fire Frequency * Silt * Spinifex * VRM | -1942.65 | 13 | 3911.31 | 3916.74 | 0.59 | 0.87 | -1942.65 | 85.27 | 0 |
| Distance to Water | -1954.4 | 4 | 3916.8 | 3917.32 | 0.54 | 0.84 | -1954.4 | 85.85 | 0 |
| Silt * Spinifex * VRM | -1947.19 | 10 | 3914.38 | 3917.52 | 0.59 | 0.86 | -1947.19 | 86.05 | 0 |
| Fire Frequency * Persistent Forest Cover * Spinifex * VRM | -1943.98 | 13 | 3913.97 | 3919.4 | 0.6 | 0.88 | -1943.98 | 87.93 | 0 |
| Persistent Forest Cover * Silt * Spinifex * VRM | -1944.01 | 13 | 3914.01 | 3919.45 | 0.6 | 0.87 | -1944.01 | 87.97 | 0 |
| Spinifex * VRM * Weathering | -1948.28 | 10 | 3916.56 | 3919.7 | 0.6 | 0.87 | -1948.28 | 88.23 | 0 |
| Distance to Water * Silt * VRM * Weathering | -1944.19 | 13 | 3914.37 | 3919.8 | 0.54 | 0.82 | -1944.19 | 88.33 | 0 |
| Fire Frequency * Spinifex * VRM * Weathering | -1944.28 | 13 | 3914.55 | 3919.99 | 0.59 | 0.87 | -1944.28 | 88.52 | 0 |
| Spinifex * VRM | -1952.4 | 7 | 3918.81 | 3920.34 | 0.6 | 0.87 | -1952.4 | 88.87 | 0 |
| Persistent Forest Cover * Spinifex * VRM * Weathering | -1946.9 | 13 | 3919.8 | 3925.23 | 0.59 | 0.86 | -1946.9 | 93.76 | 0 |
| Distance to Water * Spinifex | -1954.98 | 7 | 3923.97 | 3925.5 | 0.54 | 0.84 | -1954.98 | 94.03 | 0 |
| Distance to Water * Persistent Forest Cover | -1955.16 | 7 | 3924.31 | 3925.85 | 0.53 | 0.84 | -1955.16 | 94.38 | 0 |
| Distance to Water * Fire Frequency | -1955.36 | 7 | 3924.72 | 3926.25 | 0.54 | 0.84 | -1955.36 | 94.78 | 0 |
| Silt * Spinifex * Weathering | -1953.27 | 10 | 3926.54 | 3929.68 | 0.56 | 0.84 | -1953.27 | 98.21 | 0 |
| Distance to Water * Spinifex * Weathering | -1953.79 | 10 | 3927.59 | 3930.73 | 0.54 | 0.83 | -1953.79 | 99.26 | 0 |
| Persistent Forest Cover * Spinifex * VRM | -1953.83 | 10 | 3927.66 | 3930.81 | 0.6 | 0.87 | -1953.83 | 99.34 | 0 |
| Silt * Spinifex * VRM * Weathering | -1949.85 | 13 | 3925.71 | 3931.14 | 0.59 | 0.85 | -1949.85 | 99.67 | 0 |
| Distance to Water * Persistent Forest Cover * Weathering | -1954.77 | 10 | 3929.55 | 3932.69 | 0.54 | 0.83 | -1954.77 | 101.22 | 0 |
| Distance to Water * Persistent Forest Cover * Spinifex | -1955.68 | 10 | 3931.37 | 3934.51 | 0.54 | 0.84 | -1955.68 | 103.04 | 0 |
| Distance to Water * Fire Frequency * Spinifex | -1955.87 | 10 | 3931.74 | 3934.88 | 0.54 | 0.84 | -1955.87 | 103.41 | 0 |
| Distance to Water * Fire Frequency * Weathering | -1955.87 | 10 | 3931.74 | 3934.88 | 0.54 | 0.84 | -1955.87 | 103.41 | 0 |
| Distance to Water * Fire Frequency * Persistent Forest Cover | -1956.05 | 10 | 3932.11 | 3935.25 | 0.53 | 0.84 | -1956.05 | 103.78 | 0 |
| Distance to Water * Fire Frequency * Persistent Forest Cover * Weathering | -1953.26 | 13 | 3932.52 | 3937.95 | 0.56 | 0.84 | -1953.26 | 106.48 | 0 |
| Fire Frequency * Persistent Forest Cover * VRM | -1957.42 | 10 | 3934.84 | 3937.99 | 0.57 | 0.84 | -1957.42 | 106.51 | 0 |
| Fire Frequency * Persistent Forest Cover * Silt * VRM | -1954.18 | 13 | 3934.37 | 3939.8 | 0.57 | 0.84 | -1954.18 | 108.33 | 0 |
| Fire Frequency * Silt * Spinifex | -1958.97 | 10 | 3937.95 | 3941.09 | 0.58 | 0.86 | -1958.97 | 109.62 | 0 |
| Silt * Spinifex | -1963.12 | 7 | 3940.24 | 3941.78 | 0.58 | 0.85 | -1963.12 | 110.31 | 0 |
| Persistent Forest Cover * Silt * Spinifex * Weathering | -1955.28 | 13 | 3936.57 | 3942 | 0.58 | 0.86 | -1955.28 | 110.53 | 0 |
| Fire Frequency * Silt * VRM | -1959.52 | 10 | 3939.04 | 3942.18 | 0.56 | 0.83 | -1959.52 | 110.71 | 0 |
| Persistent Forest Cover * Silt * Spinifex | -1959.96 | 10 | 3939.93 | 3943.07 | 0.58 | 0.86 | -1959.96 | 111.6 | 0 |
| Silt * Weathering | -1964.23 | 7 | 3942.45 | 3943.99 | 0.53 | 0.81 | -1964.23 | 112.52 | 0 |
| Distance to Water * Fire Frequency * Spinifex * Weathering | -1956.59 | 13 | 3939.18 | 3944.62 | 0.53 | 0.83 | -1956.59 | 113.15 | 0 |
| Distance to Water * Fire Frequency * Persistent Forest Cover * Spinifex | -1958.54 | 13 | 3943.08 | 3948.52 | 0.53 | 0.83 | -1958.54 | 117.04 | 0 |
| Fire Frequency * Silt * Spinifex * Weathering | -1958.87 | 13 | 3943.73 | 3949.16 | 0.58 | 0.85 | -1958.87 | 117.69 | 0 |
| Fire Frequency * Persistent Forest Cover * Silt * Spinifex | -1958.96 | 13 | 3943.91 | 3949.34 | 0.58 | 0.85 | -1958.96 | 117.87 | 0 |
| Silt * VRM | -1967.25 | 7 | 3948.5 | 3950.04 | 0.57 | 0.82 | -1967.25 | 118.57 | 0 |
| Fire Frequency * Silt * VRM * Weathering | -1959.74 | 13 | 3945.48 | 3950.91 | 0.56 | 0.83 | -1959.74 | 119.44 | 0 |
| Persistent Forest Cover * Silt * Weathering | -1963.89 | 10 | 3947.77 | 3950.92 | 0.54 | 0.81 | -1963.89 | 119.44 | 0 |
| Fire Frequency * VRM | -1967.96 | 7 | 3949.92 | 3951.46 | 0.57 | 0.83 | -1967.96 | 119.99 | 0 |
| Fire Frequency * Silt | -1969.69 | 7 | 3953.39 | 3954.92 | 0.55 | 0.83 | -1969.69 | 123.45 | 0 |
| Persistent Forest Cover * Silt | -1969.98 | 7 | 3953.96 | 3955.5 | 0.54 | 0.82 | -1969.98 | 124.02 | 0 |
| Silt | -1973.86 | 4 | 3955.71 | 3956.24 | 0.55 | 0.82 | -1973.86 | 124.76 | 0 |
| Silt * VRM * Weathering | -1967.18 | 10 | 3954.36 | 3957.5 | 0.58 | 0.82 | -1967.18 | 126.03 | 0 |
| Persistent Forest Cover * Silt * VRM * Weathering | -1963.15 | 13 | 3952.3 | 3957.73 | 0.55 | 0.82 | -1963.15 | 126.26 | 0 |
| Distance to Water * Weathering | -1971.13 | 7 | 3956.25 | 3957.79 | 0.51 | 0.81 | -1971.13 | 126.31 | 0 |
| Fire Frequency * VRM * Weathering | -1968.41 | 10 | 3956.83 | 3959.97 | 0.56 | 0.83 | -1968.41 | 128.5 | 0 |
| Persistent Forest Cover * Silt * VRM | -1969.1 | 10 | 3958.2 | 3961.34 | 0.55 | 0.81 | -1969.1 | 129.87 | 0 |
| Fire Frequency * Persistent Forest Cover * Silt * Weathering | -1965.11 | 13 | 3956.21 | 3961.65 | 0.55 | 0.83 | -1965.11 | 130.18 | 0 |
| Fire Frequency * Silt * Weathering | -1969.68 | 10 | 3959.35 | 3962.49 | 0.55 | 0.83 | -1969.68 | 131.02 | 0 |
| Fire Frequency * Persistent Forest Cover * Silt | -1970.02 | 10 | 3960.03 | 3963.18 | 0.55 | 0.83 | -1970.02 | 131.7 | 0 |
| Persistent Forest Cover * VRM | -1976.22 | 7 | 3966.45 | 3967.98 | 0.54 | 0.81 | -1976.22 | 136.51 | 0 |
| Fire Frequency * Persistent Forest Cover * VRM * Weathering | -1968.42 | 13 | 3962.85 | 3968.28 | 0.56 | 0.83 | -1968.42 | 136.81 | 0 |
| Spinifex * Weathering | -1977.54 | 7 | 3969.08 | 3970.61 | 0.59 | 0.86 | -1977.54 | 139.14 | 0 |
| VRM * Weathering | -1977.63 | 7 | 3969.27 | 3970.8 | 0.53 | 0.81 | -1977.63 | 139.33 | 0 |
| Persistent Forest Cover * VRM * Weathering | -1975.39 | 10 | 3970.78 | 3973.93 | 0.57 | 0.82 | -1975.39 | 142.46 | 0 |
| Fire Frequency * Spinifex * Weathering | -1976.17 | 10 | 3972.35 | 3975.49 | 0.59 | 0.86 | -1976.17 | 144.02 | 0 |
| Persistent Forest Cover * Spinifex * Weathering | -1976.41 | 10 | 3972.83 | 3975.97 | 0.58 | 0.86 | -1976.41 | 144.5 | 0 |
| Spinifex | -1986.07 | 4 | 3980.14 | 3980.67 | 0.56 | 0.84 | -1986.07 | 149.2 | 0 |
| Fire Frequency * Persistent Forest Cover * Weathering | -1980.17 | 10 | 3980.35 | 3983.49 | 0.57 | 0.84 | -1980.17 | 152.02 | 0 |
| Fire Frequency * Persistent Forest Cover * Spinifex * Weathering | -1976.11 | 13 | 3978.23 | 3983.66 | 0.59 | 0.87 | -1976.11 | 152.19 | 0 |
| Fire Frequency * Weathering | -1984.41 | 7 | 3982.82 | 3984.36 | 0.57 | 0.85 | -1984.41 | 152.89 | 0 |
| Distance to Water * Persistent Forest Cover * Spinifex * Weathering | -1976.62 | 13 | 3979.23 | 3984.66 | 0.55 | 0.82 | -1976.62 | 153.19 | 0 |
| Fire Frequency * Spinifex | -1984.69 | 7 | 3983.38 | 3984.92 | 0.56 | 0.85 | -1984.69 | 153.45 | 0 |
| Persistent Forest Cover * Spinifex | -1985.05 | 7 | 3984.1 | 3985.63 | 0.56 | 0.84 | -1985.05 | 154.16 | 0 |
| Fire Frequency * Persistent Forest Cover * Spinifex | -1982.36 | 10 | 3984.73 | 3987.87 | 0.57 | 0.86 | -1982.36 | 156.4 | 0 |
| Fire Frequency * Persistent Forest Cover | -1987.02 | 7 | 3988.04 | 3989.57 | 0.55 | 0.83 | -1987.02 | 158.1 | 0 |
| Fire Frequency | -1993.57 | 4 | 3995.14 | 3995.67 | 0.53 | 0.82 | -1993.57 | 164.2 | 0 |
| Persistent Forest Cover * Weathering | -1992.77 | 7 | 3999.54 | 4001.08 | 0.54 | 0.81 | -1992.77 | 169.61 | 0 |
| Weathering | -1997.01 | 4 | 4002.03 | 4002.55 | 0.55 | 0.82 | -1997.01 | 171.08 | 0 |
| Persistent Forest Cover | -1997.23 | 4 | 4002.45 | 4002.98 | 0.53 | 0.82 | -1997.23 | 171.51 | 0 |
| Distance | -2003.87 | 2 | 4011.75 | 4011.9 | 0.51 | 0.79 | -2003.87 | 180.43 | 0 |
| VRM | -2003.87 | 4 | 4015.75 | 4016.27 | 0.51 | 0.79 | -2003.87 | 184.8 | 0 |
| Null | -3701.47 | 1 | 7404.94 | 7404.99 | 0 | 0.31 | -3701.47 | 3573.52 | 0 |

**Appendix S14. *ResistanceGA* bootstrap analysis**

Bootstrap results for single- and multi-surface optimised layers, including the mean across bootstrap iterations for: log-likelihood (LL), root mean squared error (RMSE), Akaike Information Criteria (AIC), sample size corrected AIC (AICc), marginal R^2^ (R^2^m), rank, and AICc weight. The number of iterations (n) and percentage of iterations (% Top Model) where the layer of interest was identified as the top-ranked model are also shown.

| **Surface** | **Mean LL** | **Mean RMSE** | **Mean AIC** | **Mean AICc** | **Mean R^2^m** | **n** | **% Top Model** | **Mean Rank** | **Mean Weight** |
| --- | --- | --- | --- | --- | --- | --- | --- | --- | --- |
| Distance to Water * Silt | -1066.29 | 0.4 | 2146.57 | 2148.73 | 0.54 | 956 | 95.6 | 1.15 | 0.92 |
| Distance to Water * Silt * Weathering | -1066.39 | 0.4 | 2152.79 | 2157.28 | 0.54 | 0 | 0 | 2.88 | 0.02 |
| Distance to Water * Silt * Spinifex | -1066.59 | 0.4 | 2153.19 | 2157.68 | 0.54 | 0 | 0 | 3.27 | 0.01 |
| Distance to Water * Silt * VRM | -1068.06 | 0.4 | 2156.12 | 2160.61 | 0.54 | 1 | 0.1 | 4.63 | 0.01 |
| Distance to Water * Persistent Forest Cover * Silt | -1068.46 | 0.4 | 2156.93 | 2161.42 | 0.54 | 0 | 0 | 5.6 | 0 |
| Distance to Water * Fire Frequency * Silt | -1069.47 | 0.4 | 2158.93 | 2163.42 | 0.54 | 0 | 0 | 6.27 | 0 |
| Distance to Water * Persistent Forest Cover * Silt * VRM | -1068.17 | 0.4 | 2162.33 | 2170.25 | 0.55 | 3 | 0.3 | 8.59 | 0 |
| Distance to Water * Persistent Forest Cover * Silt * Spinifex | -1069.07 | 0.4 | 2164.14 | 2172.05 | 0.54 | 0 | 0 | 9.45 | 0 |
| Distance to Water * Fire Frequency * Silt * Weathering | -1069.42 | 0.4 | 2164.83 | 2172.74 | 0.54 | 0 | 0 | 9.8 | 0 |
| Distance to Water * Silt * Spinifex * Weathering | -1071.22 | 0.4 | 2168.44 | 2176.35 | 0.54 | 0 | 0 | 12.47 | 0 |
| Distance to Water * Fire Frequency * Persistent Forest Cover * Silt | -1071.36 | 0.4 | 2168.73 | 2176.64 | 0.54 | 0 | 0 | 12.92 | 0 |
| Distance to Water * Silt * Spinifex * VRM | -1073.33 | 0.4 | 2172.67 | 2180.58 | 0.55 | 0 | 0 | 13.84 | 0 |
| Distance to Water * Persistent Forest Cover * Silt * Weathering | -1073.42 | 0.4 | 2172.85 | 2180.76 | 0.54 | 0 | 0 | 15.74 | 0 |
| Distance to Water * Fire Frequency * VRM | -1078.56 | 0.4 | 2177.12 | 2181.61 | 0.57 | 24 | 2.4 | 15.58 | 0.02 |
| Distance to Water * Spinifex * VRM | -1078.8 | 0.4 | 2177.59 | 2182.08 | 0.56 | 3 | 0.3 | 16.05 | 0 |
| Distance to Water * Fire Frequency * Silt * Spinifex | -1074.7 | 0.4 | 2175.4 | 2183.31 | 0.54 | 0 | 0 | 16.32 | 0 |
| Distance to Water * Persistent Forest Cover * VRM | -1081 | 0.41 | 2181.99 | 2186.48 | 0.54 | 2 | 0.2 | 19.75 | 0 |
| Distance to Water * VRM | -1086.03 | 0.41 | 2186.06 | 2188.21 | 0.55 | 0 | 0 | 24.45 | 0 |
| Distance to Water * Spinifex * VRM * Weathering | -1077.31 | 0.4 | 2180.62 | 2188.53 | 0.57 | 8 | 0.8 | 18.95 | 0 |
| Distance to Water * Fire Frequency * VRM * Weathering | -1078.12 | 0.4 | 2182.24 | 2190.15 | 0.57 | 0 | 0 | 20.12 | 0 |
| Distance to Water * Fire Frequency * Silt * VRM | -1078.92 | 0.4 | 2183.83 | 2191.75 | 0.57 | 0 | 0 | 22.42 | 0 |
| Distance to Water | -1092.56 | 0.41 | 2193.11 | 2193.84 | 0.54 | 2 | 0.2 | 31.83 | 0 |
| Distance to Water * VRM * Weathering | -1084.79 | 0.41 | 2189.58 | 2194.07 | 0.54 | 0 | 0 | 29.26 | 0 |
| Fire Frequency * Spinifex * VRM | -1086.14 | 0.41 | 2192.27 | 2196.76 | 0.58 | 0 | 0 | 31.39 | 0 |
| Distance to Water * Fire Frequency * Spinifex * VRM | -1081.98 | 0.4 | 2189.96 | 2197.87 | 0.58 | 0 | 0 | 29.32 | 0 |
| Spinifex * VRM | -1091.48 | 0.41 | 2196.96 | 2199.11 | 0.6 | 0 | 0 | 37.86 | 0 |
| Distance to Water * Persistent Forest Cover * VRM * Weathering | -1083.55 | 0.41 | 2193.1 | 2201.01 | 0.55 | 0 | 0 | 32.7 | 0 |
| Distance to Water * Persistent Forest Cover | -1092.55 | 0.41 | 2199.11 | 2201.26 | 0.53 | 0 | 0 | 39.65 | 0 |
| Distance to Water * Spinifex | -1092.68 | 0.41 | 2199.36 | 2201.51 | 0.54 | 0 | 0 | 39.95 | 0 |
| Distance to Water * Persistent Forest Cover * Spinifex * VRM | -1083.81 | 0.41 | 2193.61 | 2201.53 | 0.55 | 0 | 0 | 33.07 | 0 |
| Silt * Spinifex * VRM | -1088.58 | 0.41 | 2197.16 | 2201.65 | 0.59 | 0 | 0 | 37.86 | 0 |
| Distance to Water * Fire Frequency | -1092.88 | 0.41 | 2199.76 | 2201.91 | 0.54 | 0 | 0 | 41.47 | 0 |
| Distance to Water * Fire Frequency * Persistent Forest Cover * VRM | -1084.13 | 0.41 | 2194.25 | 2202.17 | 0.55 | 0 | 0 | 34.22 | 0 |
| Spinifex * VRM * Weathering | -1089.49 | 0.41 | 2198.99 | 2203.48 | 0.6 | 0 | 0 | 42.1 | 0 |
| Distance to Water * Silt * VRM * Weathering | -1085.19 | 0.41 | 2196.37 | 2204.28 | 0.54 | 0 | 0 | 37.64 | 0 |
| Silt * Spinifex * Weathering | -1090.45 | 0.41 | 2200.89 | 2205.38 | 0.56 | 0 | 0 | 41.54 | 0 |
| Silt * Weathering | -1095.13 | 0.41 | 2204.26 | 2206.42 | 0.53 | 0 | 0 | 47 | 0 |
| Distance to Water * Spinifex * Weathering | -1091.47 | 0.41 | 2202.95 | 2207.43 | 0.54 | 0 | 0 | 46.05 | 0 |
| Fire Frequency * Silt * Spinifex * VRM | -1086.8 | 0.41 | 2199.6 | 2207.52 | 0.59 | 0 | 0 | 43.24 | 0 |
| Fire Frequency * Spinifex * VRM * Weathering | -1087.3 | 0.41 | 2200.6 | 2208.52 | 0.59 | 0 | 0 | 44.43 | 0 |
| Distance to Water * Persistent Forest Cover * Weathering | -1092.07 | 0.41 | 2204.13 | 2208.62 | 0.54 | 0 | 0 | 48.89 | 0 |
| Persistent Forest Cover * Silt * Spinifex * VRM | -1087.4 | 0.41 | 2200.81 | 2208.72 | 0.6 | 0 | 0 | 44.86 | 0 |
| Silt * Spinifex | -1096.63 | 0.41 | 2207.25 | 2209.41 | 0.58 | 0 | 0 | 51.44 | 0 |
| Silt * VRM | -1096.76 | 0.41 | 2207.53 | 2209.68 | 0.57 | 0 | 0 | 52.32 | 0 |
| Persistent Forest Cover * Spinifex * VRM | -1092.68 | 0.41 | 2205.37 | 2209.86 | 0.6 | 0 | 0 | 52.51 | 0 |
| Fire Frequency * Persistent Forest Cover * Spinifex * VRM | -1088.16 | 0.4 | 2202.31 | 2210.23 | 0.6 | 0 | 0 | 47.88 | 0 |
| Distance to Water * Persistent Forest Cover * Spinifex | -1092.91 | 0.41 | 2205.82 | 2210.31 | 0.53 | 0 | 0 | 50.58 | 0 |
| Distance to Water * Fire Frequency * Spinifex | -1093.01 | 0.41 | 2206.02 | 2210.51 | 0.53 | 0 | 0 | 52.27 | 0 |
| Distance to Water * Fire Frequency * Weathering | -1093.01 | 0.41 | 2206.02 | 2210.51 | 0.53 | 0 | 0 | 52.42 | 0 |
| Silt | -1100.94 | 0.41 | 2209.88 | 2210.6 | 0.55 | 0 | 0 | 55.44 | 0 |
| Distance to Water * Fire Frequency * Persistent Forest Cover | -1093.11 | 0.41 | 2206.22 | 2210.71 | 0.53 | 0 | 0 | 54.09 | 0 |
| Fire Frequency * Persistent Forest Cover * VRM | -1093.17 | 0.41 | 2206.33 | 2210.82 | 0.57 | 0 | 0 | 50.17 | 0 |
| Persistent Forest Cover * Spinifex * VRM * Weathering | -1088.68 | 0.41 | 2203.36 | 2211.27 | 0.59 | 0 | 0 | 49.09 | 0 |
| Fire Frequency * Silt * VRM | -1093.69 | 0.41 | 2207.38 | 2211.87 | 0.55 | 0 | 0 | 52.11 | 0 |
| Silt * Spinifex * VRM * Weathering | -1089.25 | 0.41 | 2204.5 | 2212.42 | 0.58 | 0 | 0 | 49.96 | 0 |
| Fire Frequency * Silt * Spinifex | -1094.48 | 0.41 | 2208.95 | 2213.44 | 0.58 | 0 | 0 | 55.11 | 0 |
| Fire Frequency * VRM | -1098.71 | 0.41 | 2211.43 | 2213.58 | 0.57 | 0 | 0 | 58.69 | 0 |
| Persistent Forest Cover * Silt | -1098.87 | 0.41 | 2211.75 | 2213.9 | 0.54 | 0 | 0 | 58.76 | 0 |
| Persistent Forest Cover * Silt * Spinifex | -1095.34 | 0.41 | 2210.68 | 2215.17 | 0.58 | 0 | 0 | 57.26 | 0 |
| Persistent Forest Cover * Silt * Weathering | -1095.43 | 0.41 | 2210.86 | 2215.35 | 0.54 | 0 | 0 | 57.72 | 0 |
| Fire Frequency * Silt | -1099.62 | 0.41 | 2213.23 | 2215.39 | 0.55 | 0 | 0 | 60.09 | 0 |
| Distance to Water * Weathering | -1099.97 | 0.41 | 2213.93 | 2216.08 | 0.51 | 0 | 0 | 62.58 | 0 |
| Fire Frequency * Persistent Forest Cover * Silt * VRM | -1091.36 | 0.41 | 2208.71 | 2216.62 | 0.57 | 0 | 0 | 54.66 | 0 |
| Distance to Water * Fire Frequency * Persistent Forest Cover * Weathering | -1091.37 | 0.41 | 2208.73 | 2216.65 | 0.56 | 0 | 0 | 55.23 | 0 |
| Silt * VRM * Weathering | -1096.69 | 0.41 | 2213.37 | 2217.86 | 0.58 | 0 | 0 | 62.93 | 0 |
| Distance to Water * Fire Frequency * Spinifex * Weathering | -1092.68 | 0.41 | 2211.37 | 2219.28 | 0.53 | 0 | 0 | 60.93 | 0 |
| Persistent Forest Cover * Silt * Spinifex * Weathering | -1092.8 | 0.41 | 2211.6 | 2219.51 | 0.58 | 0 | 0 | 59.45 | 0 |
| Persistent Forest Cover * Silt * VRM | -1097.83 | 0.41 | 2215.66 | 2220.15 | 0.55 | 0 | 0 | 67.02 | 0 |
| Persistent Forest Cover * VRM | -1102.36 | 0.41 | 2218.72 | 2220.88 | 0.53 | 0 | 0 | 70.31 | 0 |
| Fire Frequency * Silt * VRM * Weathering | -1093.84 | 0.41 | 2213.68 | 2221.59 | 0.56 | 0 | 0 | 63.94 | 0 |
| VRM * Weathering | -1102.97 | 0.41 | 2219.94 | 2222.09 | 0.53 | 0 | 0 | 72.56 | 0 |
| Distance to Water * Fire Frequency * Persistent Forest Cover * Spinifex | -1094.12 | 0.41 | 2214.24 | 2222.15 | 0.53 | 0 | 0 | 64.64 | 0 |
| Fire Frequency * VRM * Weathering | -1098.91 | 0.41 | 2217.83 | 2222.32 | 0.56 | 0 | 0 | 69.58 | 0 |
| Fire Frequency * Persistent Forest Cover * Silt * Spinifex | -1094.21 | 0.41 | 2214.42 | 2222.33 | 0.58 | 0 | 0 | 64.5 | 0 |
| Fire Frequency * Silt * Spinifex * Weathering | -1094.27 | 0.41 | 2214.54 | 2222.45 | 0.58 | 0 | 0 | 64.81 | 0 |
| Fire Frequency * Silt * Weathering | -1099.58 | 0.41 | 2219.16 | 2223.65 | 0.55 | 0 | 0 | 70.64 | 0 |
| Fire Frequency * Persistent Forest Cover * Silt | -1099.72 | 0.41 | 2219.43 | 2223.92 | 0.55 | 0 | 0 | 71.4 | 0 |
| Persistent Forest Cover * Silt * VRM * Weathering | -1095.16 | 0.41 | 2216.31 | 2224.23 | 0.55 | 0 | 0 | 68.96 | 0 |
| Spinifex | -1108.44 | 0.41 | 2224.88 | 2225.61 | 0.56 | 1 | 0.1 | 74.23 | 0 |
| Spinifex * Weathering | -1105.19 | 0.41 | 2224.38 | 2226.54 | 0.59 | 0 | 0 | 75.43 | 0 |
| Fire Frequency * Persistent Forest Cover * Silt * Weathering | -1096.71 | 0.41 | 2219.43 | 2227.34 | 0.54 | 0 | 0 | 71.6 | 0 |
| Persistent Forest Cover * VRM * Weathering | -1101.74 | 0.41 | 2223.49 | 2227.98 | 0.56 | 0 | 0 | 78.2 | 0 |
| Fire Frequency * Persistent Forest Cover * VRM * Weathering | -1099 | 0.41 | 2224 | 2231.91 | 0.56 | 0 | 0 | 78.44 | 0 |
| Fire Frequency * Spinifex | -1108.18 | 0.41 | 2230.35 | 2232.5 | 0.56 | 0 | 0 | 81.67 | 0 |
| Persistent Forest Cover * Spinifex | -1108.24 | 0.41 | 2230.49 | 2232.64 | 0.56 | 0 | 0 | 81.85 | 0 |
| Fire Frequency * Weathering | -1108.42 | 0.41 | 2230.85 | 2233 | 0.57 | 0 | 0 | 83.95 | 0 |
| Fire Frequency * Spinifex * Weathering | -1104.31 | 0.41 | 2228.63 | 2233.12 | 0.59 | 0 | 0 | 81.28 | 0 |
| Fire Frequency | -1112.27 | 0.41 | 2232.54 | 2233.27 | 0.53 | 0 | 0 | 83.97 | 0 |
| Persistent Forest Cover * Spinifex * Weathering | -1104.55 | 0.41 | 2229.11 | 2233.6 | 0.58 | 0 | 0 | 82.06 | 0 |
| Fire Frequency * Persistent Forest Cover | -1109.15 | 0.41 | 2232.31 | 2234.46 | 0.55 | 0 | 0 | 84.1 | 0 |
| Weathering | -1113.78 | 0.41 | 2235.57 | 2236.3 | 0.54 | 0 | 0 | 89.42 | 0 |
| Fire Frequency * Persistent Forest Cover * Weathering | -1106.03 | 0.41 | 2232.05 | 2236.54 | 0.57 | 0 | 0 | 85.46 | 0 |
| Distance | -1116.42 | 0.42 | 2236.83 | 2237.04 | 0.51 | 0 | 0 | 87.94 | 0 |
| Persistent Forest Cover | -1114.69 | 0.41 | 2237.37 | 2238.1 | 0.53 | 0 | 0 | 88.59 | 0 |
| Persistent Forest Cover * Weathering | -1111.31 | 0.41 | 2236.61 | 2238.77 | 0.54 | 0 | 0 | 90.69 | 0 |
| Distance to Water * Persistent Forest Cover * Spinifex * Weathering | -1102.79 | 0.41 | 2231.58 | 2239.5 | 0.55 | 0 | 0 | 85.49 | 0 |
| Fire Frequency * Persistent Forest Cover * Spinifex | -1107.91 | 0.41 | 2235.82 | 2240.31 | 0.57 | 0 | 0 | 88.13 | 0 |
| VRM | -1116.42 | 0.42 | 2240.83 | 2241.56 | 0.51 | 0 | 0 | 92.19 | 0 |
| Fire Frequency * Persistent Forest Cover * Spinifex * Weathering | -1104.81 | 0.41 | 2235.61 | 2243.53 | 0.59 | 0 | 0 | 88.81 | 0 |

**Appendix S15. *ResistanceGA* MLPE diagnostic plots and layer transformations**

*Diagnostic plots for MLPE residuals in the top-ranked model:*

The top-ranked linear mixed effects model fit with the optimised composite surface (distance to water and silt) as the predictor variable and the Euclidean genetic distance as the response variable (with a population level random effect to account for pairwise comparisons). Although residuals are slightly heavy tailed (c,d), resistance distance increases linearly with increasing genetic distance (a), and there is no obvious systematic bias in the distribution of residuals (b). We therefore chose to proceed with this model, as the tails are symmetrical and would likely lead to more conservative estimates for the fixed effect (Pinheiro & Bates 2000). Furthermore, we compared this model with two alternatives: a model refit with heavy-tailed errors, using the Student’s t distribution (Pinheiro et al. 2001) in the R package *heavy* (Osorio 2019) and a model where outliers were removed (standardized residuals greater than 2.5 standard deviations; Tremblay & Ransijn 2020). In all cases, results were consistent.


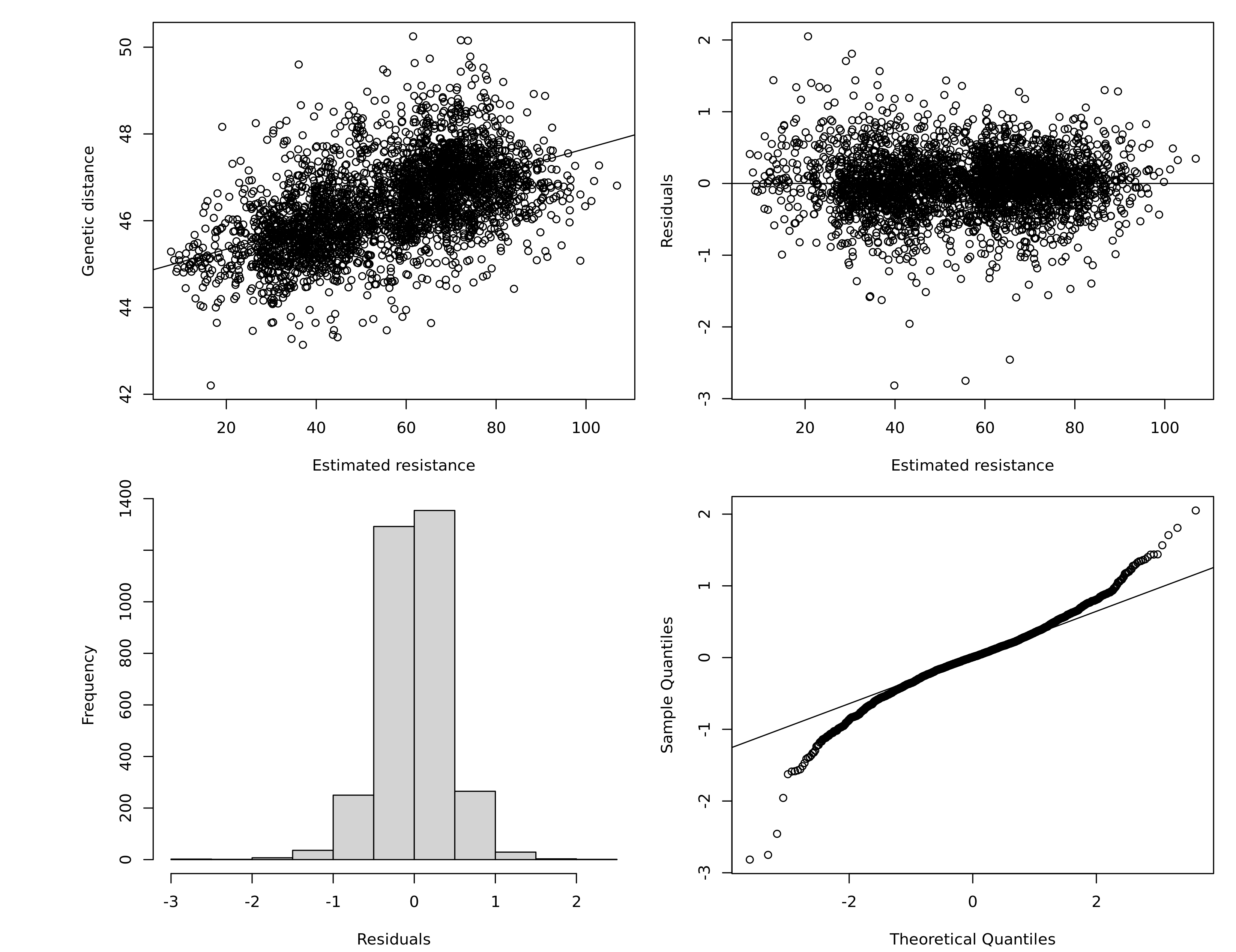


**a)**

**c)**

**b)**

**d)**

*Transformations for the optimised surfaces used to create the final composite layer:*

Optimisation resulted in both layers undergoing an inverse-reverse monomolecular transformation, with a shape parameter of 6.71 and 0.59, and a maximum value of 1621.32 and 2352.32 (for distance to water and silt, respectively).

| **Distance to water**  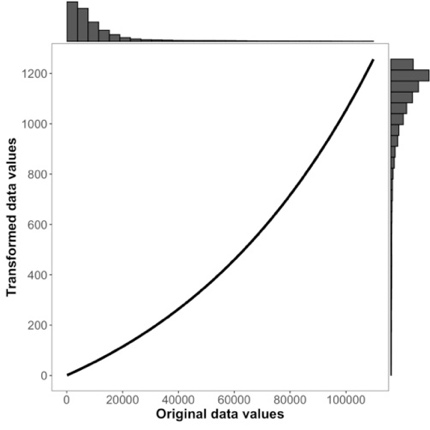 | **Silt**  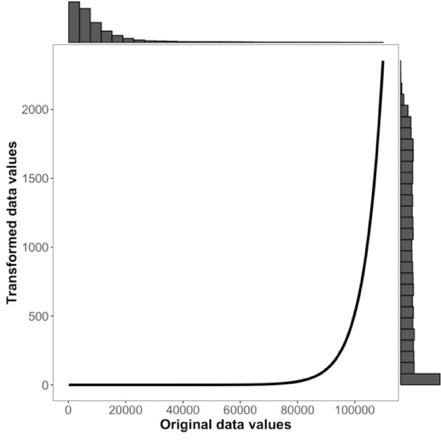 |
| --- | --- |

**Appendix S16. *ResistanceGA* MLPE model summary**

a) MLPE model summary including model estimates and confidence intervals (CI) computed using parametric bootstrapping; and b) the contribution of the single surfaces to the final composite layer.

| *a) MLPE model summary* | |  |  |  |  |
| --- | --- | --- | --- | --- | --- |
|  | **Estimate** | **Std. Error** | **t value** | **2.5% CI** | **97.5% CI** |
| Intercept | 46.389 | 0.122 | 381.566 | 46.256 | 46.518 |
| Distance to Water*Silt | 0.743 | 0.009 | 81.899 | 0.708 | 0.778 |
| *b) Layer contributions* | |  |  |  |  |
| **surface** | **Mean (%)** |  |  |  |  |
| Distance to Water | 86.75 |  |  |  |  |
| Silt | 13.25 |  |  |  |  |

**References**

Akaike H. 1974. A New Look at the Statistical Model Identification. IEEE Transactions on Automatic Control **19**:716–723.

Anderson RP, Raza A. 2010. The effect of the extent of the study region on GIS models of species geographic distributions and estimates of niche evolution: preliminary tests with montane rodents (genus *Nephelomys*) in Venezuela. Journal of Biogeography **37**:1378–1393.

Begg RJ. 1981. The small mammals of Little Nourlangie Rock, N.T III. Ecology of *Dasyurus hallucatus*, the northern quoll (marsupialia: Dasyuridae). Wildlife Research **8**:73–85.

Bradley AJ, Kemper CM, Kitchener DJ, Humphreys WF, Howb RA. 1987. Small mammals of the mitchell plateau region, kimberley, Western Australia. Wildlife Research **14**:397–413.

Braithwaite RW, Griffiths AD. 1994. Demographic variation and range contraction in the northern quoll, *Dasyurus hallucatus* (Marsupialia: Dasyuridae). Wildlife Research **21**:203–217.

Burnett S. 1997. Colonizing Cane Toads cause population declines in native predators: reliable anecdotal information and management implications. Pacific Conservation Biology **3**:65–72.

Caye K, Deist TM, Martins H, Michel O, François O. 2016. TESS3: Fast inference of spatial population structure and genome scans for selection. Molecular Ecology Resources **16**:540–548.

Caye K, Francois O. 2016. tess3r: Inference of spatial population genetic structure. R package version 1.1.0.

Chan R, Dunlop J, Spencer PBS. 2020. Highly promiscuous paternity in mainland and island populations of the endangered Northern Quoll. Journal of Zoology **310**:210–220.

Clemente CJ, Dick TJM, Wheatley R, Gaschk J, Nasir AFAA, Cameron SF, Wilson RS. 2019. Moving in complex environments: A biomechanical analysis of locomotion on inclined and narrow substrates. Journal of Experimental Biology **222**:jeb189654.

Cooper CE, Withers PC. 2010. Comparative physiology of Australian quolls (*Dasyurus*; Marsupialia). Journal of Comparative Physiology B **180**:857–868.

Cowan MA, Dunlop JA, Turner JM, Moore HA, Nimmo DG. 2020. Artificial refuges to combat habitat loss for an endangered marsupial predator: How do they measure up? Conservation Science and Practice **2**:e204.

Cremona T, Crowther MS, Webb JK. 2017. High mortality and small population size prevent population recovery of a reintroduced mesopredator. Animal Conservation **20**:555–563.

DBCA. 2019. NatureMap: Mapping Western Australia’s biodiversity V 1.8.3.4. Available from https://naturemap.dbca.wa.gov.au/.

Department of Agriculture and Food WA. 2003. Characteristic Soils of Western Australia. Available from https://researchlibrary.agric.wa.gov.au/gis_maps/15/.

Diete RL, Meek PD, Dickman CR, Lisle A, Leung LK-P. 2017. Diel activity patterns of northern Australian small mammals: variation, fixity, and plasticity. Journal of Mammalogy **98**:848–857.

Dunlop JA, Rayner K, Doherty TS. 2017. Dietary flexibility in small carnivores: A case study on the endangered northern quoll, *Dasyurus hallucatus*. Journal of Mammalogy **98**:858–866.

ESRI. 2018. ArcGIS Desktop: Release 10.6. Redlands, CA: Environmental Systems Research Institute.

Evans J. 2020. spatialEco. Available from https://github.com/jeffreyevans/spatialEc.

Frichot E, François O. 2015. LEA: An R package for landscape and ecological association studies. Methods in Ecology and Evolution **6**:925–929.

Friend GR, Taylor JA. 1985. Habitat preferences of small mammals in tropical open‐forest of the Northern Territory. Australian Journal of Ecology **10**:173–185.

Furby S. 2018. Woody Vegetation Extent and Change 1972-2018, Western Australia – Whole State Product, 2018 Update of the Land Monitor II Project.

Furby S, Wallace J, Caccetta P. 2007. Monitoring sparse perennial vegetation cover over Australia using sequences of Landsat imagery. Page International Conference on Environmental Informatics. Bangkok, Thailand.

Gallant J, Wilson N, Dowling T, Read A, Inskeep C. 2011. SRTM-derived 1 Second Digital Elevation Models Version 1.0. Record 1. Geoscience Australia, Canberra. Available from https://ecat.ga.gov.au/geonetwork/srv/eng/catalog.search#/metadata/72759.

Geoscience Australia. 1997. Australia’s River Basins 1997. Available from http://pid.geoscience.gov.au/dataset/ga/42343.

Gibson LA, McKenzie NL. 2012. Occurrence of non-volant mammals on islands along the Kimberley coast of Western Australia. Pages 15–40 Records of the Western Australian Museum. NA.

Griffiths AD, Brook BW. 2015. Fire impacts recruitment more than survival of small-mammals in a tropical savanna. Ecosphere **6**:1–22.

Griffiths AD, Garnett ST, Brook BW. 2015. Fire frequency matters more than fire size: Testing the pyrodiversity-biodiversity paradigm for at-risk small mammals in an Australian tropical savanna. Biological Conservation **186**:337–346. Elsevier Ltd, NA. Available from http://dx.doi.org/10.1016/j.biocon.2015.03.021.

Griffiths AD, Rankmore B, Brennan K, Woinarski JCZ. 2017. Demographic evaluation of translocating the threatened northern quoll to two Australian islands. Wildlife Research **44**:238–247.

Harwood TD. 2019. 9s climatology for continental Australia 1976-2005: BIOCLIM variable suite. CSIRO Data Collection v1.

Harwood TD, Donohue R, Harman I, McVicar T, Ota N, Perry J, Williams K. 2016. 9s climatology for continental Australia 1976-2005: Summary variables with elevation and radiative adjustment. CSIRO Data Collection v3.

Hastie T, Tibshirani R, Friedman JH. 2009. The elements of statistical learning: data mining, inference, and prediction, 2nd edition. Springer series in statistics New York, New York.

Heiniger J, Cameron SF, Madsen T, Niehaus AC, Wilson RS. 2020. Demography and spatial requirements of the endangered northern quoll on Groote Eylandt. Wildlife Research **47**:224–238. NA.

Henderson M. 2015. The effects of mining infrastructure on northern quoll movement and habitat. Edith Cowan University.

Hernandez-Santin L, Goldizen AW, Fisher DO. 2016. Introduced predators and habitat structure influence range contraction of an endangered native predator, the northern quoll. Biological Conservation **203**:160–167.

Hijmans RJ. 2020. raster: Geographic Data Analysis and Modeling. Available from https://cran.r-project.org/package=raster.

Hohnen R, Tuft KD, Legge SM, Hillyer M, Spencer PBS, Radford IJ, Johnson CN, Burridge CP. 2016. Rainfall and topography predict gene flow among populations of the declining northern quoll (*Dasyurus hallucatus*). Conservation Genetics **17**:1213–1228.

Holmes K, Griffin T, Odgers N. 2014. Soil and Landscape Grid Digital Soil Property Maps for Western Australia (3" resolution). v4. CSIRO. Data Collection.

Ibbett M, Woinarski JCZ, Oakwood M. 2018. Declines in the mammal assemblage of a rugged sandstone environment in Kakadu National Park, Northern Territory, Australia. Australian Mammalogy **40**:181–187.

Jolly CJ, Kelly E, Gillespie GR, Phillips B, Webb JK. 2018. Out of the frying pan: Reintroduction of toad-smart northern quolls to southern Kakadu National Park. Austral Ecology **43**:139–149.

Jombard T, Devillard S, Balloux F. 2010. Discriminant analysis of principal components: a new method for the analysis of genetically structured populations. BMC Genetics **11**:1–15.

Jombart T. 2008. Adegenet: A R package for the multivariate analysis of genetic markers. Bioinformatics **24**:1403–1405.

Jombart T, Ahmed I. 2011. adegenet 1.3-1: New tools for the analysis of genome-wide SNP data. Bioinformatics **27**:3070–3071.

Kelly E, Phillips BL. 2017. Get smart: native mammal develops toad-smart behavior in response to a toxic invader. Behavioral Ecology **28**:854–858. NA.

Klein DJ, Randić M. 1993. Resistance distance. Journal of Mathematical Chemistry **12**:81–95.

Landgate. 2012. Medium-Scale Topographic database (GIS dataset - inland flat and coastal flat polygon features). Available from https://www0.landgate.wa.gov.au/business-and-government/land-data/topographic-data.

Landgate. 2017. Medium-Scale Topographic database (GIS dataset - linear and polygon features). Available from https://www0.landgate.wa.gov.au/business-and-government/land-data/topographic-data.

Landgate. 2019. Medium-Scale Topographic database (GIS dataset - point features). Available from https://www0.landgate.wa.gov.au/business-and-government/land-data/topographic-data.

Leutner B, Horning N, Schwalb-Willmann J. 2019. RStoolbox: Tools for Remote Sensing Data Analysis. Available from https://cran.r-project.org/package=RStoolbox.

Li F, Jupp DLB, Thankappan M, Lymburner L, Mueller N, Lewis A, Held A. 2012. A physics-based atmospheric and BRDF correction for Landsat data over mountainous terrain. Remote Sensing of Environment **124**:756–770. Elsevier B.V. Available from http://dx.doi.org/10.1016/j.rse.2012.06.018.

McRae BH, Dickson BG, Keitt TH, Shah VB. 2008. Using circuit theory to model connectivity in ecology, evolution, and conservation. Ecology **89**:2712–2724.

Molloy SW, Davis RA, Dunlop JA, van Etten EJB. 2017. Applying surrogate species presences to correct sample bias in species distribution models: A case study using the Pilbara population of the Northern Quoll. Nature Conservation **18**:27–46.

Moore HA, Dunlop JA, Valentine LE, Woinarski JCZ, Ritchie EG, Watson DM, Nimmo DG. 2019. Topographic ruggedness and rainfall mediate geographic range contraction of a threatened marsupial predator. Diversity and Distributions **25**:1818–1831.

Moro D, Dunlop J, Williams MR. 2019. Northern quoll persistence is most sensitive to survivorship of juveniles. Wildlife Research **46**:165–175.

Murchison EP et al. 2012. Genome sequencing and analysis of the Tasmanian devil and its transmissible cancer. Cell **148**:780–791. Elsevier Inc. Available from http://dx.doi.org/10.1016/j.cell.2011.11.065.

NAFI. 2019. Modis annual fire scar images (2000 - 2018). Available from https://www.firenorth.org.au/nafi3/.

Newman P, Raymond B, VanDerWal J, Belbin L. 2019. ALA4R: Atlas of Living Australia (ALA) data and resources in R. R package version 1.7.0.

O’Brien L. 2020. slga: Data Access Tools for the Soil and Landscape Grid of Australia. Available from https://cran.r-project.org/package=slga.

O’Donnell S, Webb JK, Shine R. 2010. Conditioned taste aversion enhances the survival of an endangered predator imperilled by a toxic invader. Journal of Applied Ecology **47**:558–565.

Oakwood M. 2000. Reproduction and demography of the northern quoll, *Dasyurus hallucatus*, in the lowland savanna of northern Australia. Australian Journal of Zoology **48**:519–539.

Oakwood M. 2002. Spatial and social organization of a carnivorous marsupial *Dasyurus hallucatus* (Marsupialia: Dasyuridae). Journal of Zoology **257**:237–248.

Oakwood M, Spratt DM. 2000. Parasites of the northern quoll, *Dasyurus hallucatus* (Marsupialia: Dasyuridae) in tropical savanna, Northern Territory. Australian Journal of Zoology **48**:79–90.

Old JM, Stannard HJ. 2020. Conservation of quolls (*Dasyurus* spp.) in captivity - a review. Australian Mammalogy **55**:383–389.

Olds LGM, Myers C, Cook H, Schembri B, Jackson C, Evans N, Charles B, Waina R, Breed WG, Taggart DA. 2017. The occurrence and relative abundance of small terrestrial mammals on Theda Station in the Northern Kimberley, Western Australia. Australian Mammalogy **39**:78–91. NA.

Olds LGM, Myers C, Reside J, Madani G, Dudley A, Potter S, Martin R, Boona E, Waina T, Taggart DA. 2016. Small terrestrial mammals on Doongan Station, in the Northern Kimberley bioregion, Western Australia. Australian Mammalogy **38**:164–176.

Osorio F. 2019. heavy: Robust estimation using heavy-tailed distributions. R package version 0.38.196. Available from https://cran.r-project.org/package=heavy.

Palmer R et al. 2016. A survey for Wijingadda (northern quoll *Dasyurus hallucatus*) and other fauna on islands in Dambimangari country in Talbot Bay (Kimberley, Western Australia). Conservation Science Western Australia **10**:1–12. NA.

Peacock D, Abbott I. 2014. When the “native cat” would “plague”: historical hyperabundance in the quoll (Marsupialia: Dasyuridae) and an assessment of the role of disease, cats and foxes in its curtailment. Australian Journal of Zoology **62**:294–344.

Peterman WE. 2018. ResistanceGA: An R package for the optimization of resistance surfaces using genetic algorithms. Methods in Ecology and Evolution **9**:1638–1647.

Peterman WE, Connette GM, Semlitsch RD, Eggert LS. 2014. Ecological resistance surfaces predict fine-scale genetic differentiation in a terrestrial woodland salamander. Molecular Ecology **23**:2402–2413.

Phillips SJ, Dudík M. 2008. Modeling of species distributions with Maxent: New extensions and a comprehensive evaluation. Ecography **31**:161–175.

Phillips SJ, Dudík M, Elith J, Graham CH, Lehmann A, Leathwick J, Ferrier S. 2009. Sample selection bias and presence-only distribution models: implications for background and pseudo-absence data. Ecological Applications **19**:181–197.

Pinheiro JC, Bates DM. 2000. Theory and Computational Methods for Linear Mixed-Effects Models. Pages 57–96 Mixed-Effects Models in S and S-PLUS. Springer-Verlag, New York. Available from http://link.springer.com/10.1007/0-387-22747-4_2.

Pinheiro JC, Liu C, Wu YN. 2001. Efficient algorithms for robust estimation in linear mixed-effects models using the multivariate t distribution. Journal of Computational and Graphical Statistics **10**:249–276.

Pollock AB. 1999. Notes on status, distribution and diet of Northern Quoll Dasyurus hallucatus in the Mackay-Bowen area, mideastern Queensland. Australian Zoologist **31**:388–395. NA.

Price O, Rankmore B, Milne D, Brock C, Tynan C, Kean L, Roeger L. 2005. Regional patterns of mammal abundance and their relationship to landscape variables in eucalypt woodlands near Darwin, northern Australia. Wildlife Research **32**:435–446.

QGIS Development Team. 2021. QGIS Geographic Information System v2.18.16. Open Source Geospatial Foundation Project. Available from http://qgis.osgeo.org.

R Core Team. 2020. R: A language and environment for statistical computing. R Foundation for Statistical Computing, Vienna, Austria. Available from https://www.r-project.org/.

Radford IJ. 2012. Threatened mammals become more predatory after small-scale prescribed fires in a high-rainfall rocky savanna. Austral Ecology **37**:926–935.

Radford IJ, Dickman CR, Start AN, Palmer C, Carnes K, Everitt C, Fairman R, Graham G, Partridge T, Thomson A. 2014. Mammals of Australia’s tropical savannas: a conceptual model of assemblage structure and regulatory factors in the Kimberley region. PLoS ONE **9**:e92341.

Radford IJ, Woolley L, Corey B, Vigilante T, Wunambal Gaambera Aboriginal Corporation ., Hatherley E, Fairman R, Carnes K, Start AN. 2020. Prescribed burning benefits threatened mammals in northern Australia. Biodiversity and Conservation **29**:2985–3007. NA.

Radosavljevic A, Anderson RP. 2014. Making better Maxent models of species distributions: Complexity, overfitting and evaluation. Journal of Biogeography **41**:629–643.

Rampant P, Zdunic K, Burrows N. 2019. UAS and Landsat imagery to determine fuel condition for fire behaviour prediction on spinifex hummock grasslands of arid Australia. International Journal of Remote Sensing **40**:9126–9139. Taylor & Francis. Available from https://doi.org/10.1080/01431161.2019.1651950.

Rew-Duffy M, Cameron SF, Freeman NJ, Wheatley R, Latimer JM, Wilson RS. 2020. Greater agility increases probability of survival in the endangered northern quoll. Journal of Experimental Biology **223**:jeb218503. NA.

Ruiz-Lopez MJ, Barelli C, Rovero F, Hodges K, Roos C, Peterman WE, Ting N. 2016. A novel landscape genetic approach demonstrates the effects of human disturbance on the Udzungwa red colobus monkey (*Procolobus gordonorum*). Heredity **116**:167–176. Nature Publishing Group.

Sappington JM, Longshore KM, Thompson DB. 2007. Quantifying Landscape Ruggedness for Animal Habitat Analysis: A Case Study Using Bighorn Sheep in the Mojave Desert. Journal of Wildlife Management **71**:1419–1426.

Schmitt LH, Bradley AJ, Kemper CM, Kitchener DJ, Humphreys WF, How RA. 1989. Ecology and physiology of the northern quoll, *Dasyurus hallucatus* (Marsupialia, Dasyuridae), at Mitchell Plateau, Kimberley, Western Australia. Journal of Zoology **217**:539–558.

Tremblay A, Ransijn J. 2020. LMERConvenienceFunctions: model selection and post-hoc analysis for (G)LMER models. R package version 3.0.

Turpin J. 2015. North Kimberley Mammals - on the fringe of the high-rainfall zone. Australian Mammalogy **37**:132–145. NA.

Turpin JM, Bamford MJ. 2015. A new population of the northern quoll (*Dasyurus hallucatus*) on the edge of the Little Sandy Desert, Western Australia. Australian Mammalogy **37**:86–91.

Ujvari B, Oakwood M, Madsen T. 2013. Queensland northern quolls are not immune to cane toad toxin. Wildlife Research **40**:228–231.

Venables WN, Ripley BD. 2002. Modern Applied Statistics with SFourth. Springer, New York. Available from http://www.stats.ox.ac.uk/pub/MASS4.

Vignali S, Lörcher F, Hegglin D, Arlettaz R, Braunisch V. 2021. Modelling the habitat selection of the bearded vulture to predict areas of potential conflict with wind energy development in the Swiss Alps. Global Ecology and Conservation **25**.

von Takach B, Scheele BC, Moore H, Murphy BP, Banks SC. 2020. Patterns of niche contraction identify vital refuge areas for declining mammals. Diversity and Distributions **26**:1467–1482.

Wilford J. 2012. A weathering intensity index for the Australian continent using airborne gamma-ray spectrometry and digital terrain analysis. Geoderma **183**–**184**:124–142. Elsevier B.V. Available from http://dx.doi.org/10.1016/j.geoderma.2010.12.022.

Wilford J, Roberts D. 2019. Weathering Intensity Model of Australia. Geoscience Australia, Canberra.

Woinarski JCZ et al. 2010. Monitoring indicates rapid and severe decline of native small mammals in Kakadu National Park, northern Australia. Wildlife Research **37**:116–126.

Xu T, Hutchinson M. 2011. ANUClim Version 6.1 User Guide. The Australian National University, Fenner School of Environment and Society. , Canberra.
